# Charting the Atomic C Interaction with Transition Metal Surfaces

**DOI:** 10.1021/acscatal.2c01562

**Published:** 2022-07-15

**Authors:** Oriol Piqué, Iskra Z. Koleva, Albert Bruix, Francesc Viñes, Hristiyan A. Aleksandrov, Georgi N. Vayssilov, Francesc Illas

**Affiliations:** † Departament de Ciència de Materials i Química Física & Institut de Química Teòrica i Computacional (IQTCUB), Universitat de Barcelona, c/ Martí i Franquès 1, 08028 Barcelona, Spain; ‡ Faculty of Chemistry and Pharmacy, University of Sofia, 1126 Sofia, Bulgaria

**Keywords:** carbon atoms, transition metal surfaces, adsorption, absorption, diffusion, descriptors, machine learning, phase diagrams

## Abstract

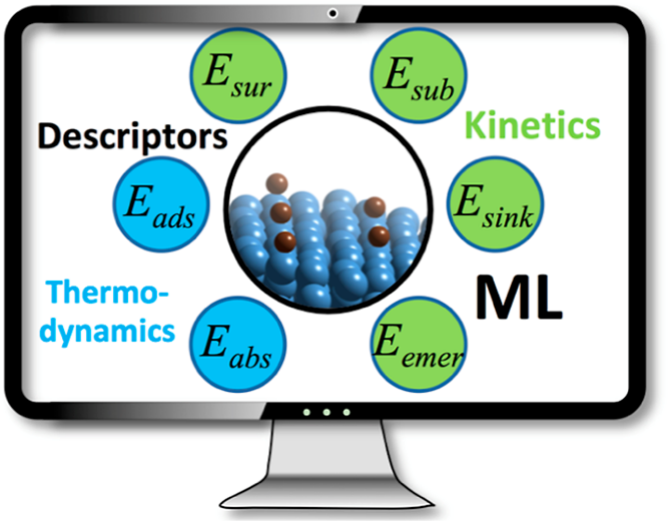

Carbon interaction with transition metal (TM) surfaces is a relevant topic in heterogeneous catalysis, either for its poisoning capability, for the recently attributed promoter role when incorporated in the subsurface, or for the formation of early TM carbides, which are increasingly used in catalysis. Herein, we present a high-throughput systematic study, adjoining thermodynamic *plus* kinetic evidence obtained by extensive density functional calculations on surface models (324 diffusion barriers located on 81 TM surfaces in total), which provides a navigation map of these interactions in a holistic fashion. Correlation between previously proposed electronic descriptors and ad/absorption energies has been tested, with the *d*-band center being found the most suitable one, although machine learning protocols also underscore the importance of the surface energy and the site coordination number. Descriptors have also been tested for diffusion barriers, with ad/absorption energies and the difference in energy between minima being the most appropriate ones. Furthermore, multivariable, polynomial, and random forest regressions show that both thermodynamic and kinetic data are better described when using a combination of different descriptors. Therefore, looking for a single perfect descriptor may not be the best quest, while combining different ones may be a better path to follow.

## 1 Introduction

Nowadays, transition metals (TMs) are ubiquitous in many areas of science and technology, from Solid State Physics through Applied Chemistry up to Materials Science, with relevance in diverse industrial fields, such as Nanotechnology and Heterogeneous Catalysis. In fact, late TMs, including noble coinage (Ni, Cu, Ag, and Au) and Pt-group (Pt, Pd, Rh, Ir, Re, and Os) metals, are in widespread use as heterogeneous catalysts^1^ for a large diversity of reactions of industrial interest, for example, ammonia synthesis, exhaust gas treatments, or the Fischer–Tropsch reaction, to name just a few.^2,3^ However, early TMs are far too active for such purposes according to *Le Sabatier* principle,^4^ adsorbing chemical moieties too strongly. Their heterogeneous catalysis interest though lies in the TM carbide (TMC) formation because the carburization of these metals diminishes their chemical activity, to the point of making TMCs viable replacements of the abovementioned late TMs in catalysis,^5^ featuring improved catalytic performance, selectivity, or poison resistance compared to Pt-group TMs.^6,7^

The relative simplicity of TM systems and the industrial importance of their applications have prompted state-of-the-art research aimed at unveiling their catalytic activity improvement and desirably coupled with materials cost reduction, as the one achieved when using Earth-abundant TMCs. To this end, nanostructuration, alloy usage, and even the design of nanoalloys have been contemplated as plausible strategies.^8,9^ The rational design of novel metal and alloy catalysts, backed up by precise *ab initio* quantum chemistry calculations on proper catalyst models, has meant a great leap forward in the quest for new TM catalysts with improved activity.^9,10^

Still, however, one main drawback of such catalysts is that, in the course of the catalyzed reaction, these get gradually deactivated over time and use, with the concomitant loss in efficiency and economic profit. The origin of this loss is the presence of poisoning agents, where carbon excels among others.^11,12^ Thus, the interaction of C with TMs is, indeed, a fundamental field of study in Heterogeneous Catalysis. Historically, from the catalytic deactivation process point of view, it is quite usual that carbon entities, going from C atoms to small C_*n*_ aggregates, are formed on the TM catalyst surface due to secondary reactions of the ongoing surface catalyzed process, generally involving organic molecules. These carbon entities can spread through the surface and eventually agglomerate forming diverse types of carbon deposits, from graphene through graphite, up to amorphous C aggregates. These surface carbon structures can cover the catalyst, restricting the access of reactants to the TM catalytic surface active sites, effectively poisoning it.

Despite this, recent experiments and computational simulations have changed the paradigm view of the low C content from a poisoner to a promoter role. For instance, subsurface C into Pd catalysts appears to favor the selective alkyne hydrogenation to olefins,^13^ and its presence, observed through scanning tunneling microscopy experiments,^14,15^ and explained by simulations based on density functional theory (DFT),^16^ shows how such subsurface C can be easily present at the surfaces of late TM surfaces and nanoparticles (NPs).^17−20^ Moreover, subsurface C plays an important role in the synthesis of graphene and carbon nanotubes (CNTs), where segregation of C atoms diluted in premolten or molten TMs appears when cooling down the system, which induces the growth of graphene layers, even CNTs.^21,22^

Furthermore, subsurface C has been found to bias the appearance of other substitutional or interstitial carbon residues in Pd,^23^ which may display even higher reactivity toward surface O and H adatoms than surface C,^24^ and to act as a gate opener for H absorption.^25^ Such subsurface moiety-mediated chemistry is nonexclusive, neither to C nor Pd. Indeed, subsurface C has been proposed to be a key player in the electrochemical conversion of CO_2_ on Cu surfaces,^26^ and interstitial C in Au NPs has been found to affect the metal electronic structure, being ultimately responsible for the three-time increment of the measure turnover frequency in the chemoselective hydrogenation of 3-nitrostyrene.^27^ Apart from subsurface C, it is worth mentioning that subsurface O can also affect the ongoing surface activity, see, for example, the recent key role of subsurface O on Cu(111) surface in CO_2_ capture and activation applications, with critical implications in environmental chemistry.^28^ Subsurface chemistry has often been ignored but is now attracting growing attention within the scientific community, seen as a change of paradigm in what surface chemical activity is concerned.^29^

Motivated by these results, the primary aim of this work is to deliver, for the first time, a broad, detailed, and holistic atomistic view of the interaction of C with TM surfaces. This is achieved by studying, by *ab initio* DFT calculations on proper surface slab models, the stability of C atoms on the Miller surfaces with an index order of 1 for all those TMs featuring a face-centered cubic (fcc), body-centered cubic (bcc), or hexagonal close-packed (hcp) bulk crystallographic structure (CS)—see Figures S1 and S2 of Section S1 of the Supporting Information. For such surfaces, the most stable surface and subsurface sites are identified and their bond strengths seized, so as to gain thermodynamic pictures and stability phase diagrams, as done earlier for fcc TM (111) surfaces.^18^ Furthermore, all sorts of C diffusion energy barriers, *E*_b_, are explored, including surface, subsurface, sinking, and emerging diffusion energy barriers for each metal surface, denoted as *E*_sur_, *E*_sub_, *E*_sink_, and *E*_emer_, respectively—see Figure S3 in Section S1 of the Supporting Information for a depiction of the different barrier types. This systematic study will provide a navigation chart of the tendency of C to poison surface active sites, to aggregate into C_*n*_ moieties, and will also provide insights on the possible formation of TMCs. Finally, the acquired amount of data allows for further analysis based on artificial intelligence (AI) and machine-learning (ML) regression algorithms, addressed at defining subgroup types of similar behavior concerning C bond strength and diffusivity, the main physicochemical descriptors defining these, as well as regressions of adsorptive and diffusive properties as a function of physicochemical descriptors.

## 2 Computational Details

Present DFT calculations have been performed using the Vienna *ab initio* simulation package (VASP) code,^30^ imposing periodic symmetry. Relaxed geometries and total energies were acquired using the Perdew–Burke–Ernzerhof (PBE) exchange-correlation functional,^31^ known to accurately describe TMs bulks and surfaces and also the interaction of C with them.^19,32^ Moreover, previous works show that relative stabilities and diffusion energy barriers, *E*_b_, are rather unbiased by the used exchange–correlation functional, with small variations between 1 and 5 kJ·mol^–1^ depending on the used functional.^19^ The valence electrons density was expanded in a plane-wave basis set with a 415 eV cutoff for the kinetic energy, while the projector augmented wave method was used to describe the interactions between core and valence electrons.^33^ Calculations were carried out with no spin polarization except for magnetic Ni, Co, and Fe TMs. A vacuum of 10 Å was found to be enough to get converged results, except for magnetic TMs, which required larger vacuums of 30 Å to avoid magnetic coupling among translationally repeated slabs.^32^ Geometry optimizations were performed until all forces acting on relaxed atoms became lower than 0.03 eV·Å^–1^, and the electronic convergence threshold was set to 10^–6^ eV.

The TMs surfaces with the Miller index maximum order of 1 have been modeled using slabs, generally including the most stable surfaces, with no step defects, thus deliberately leaving apart surfaces with a Miller index maximum order of 2 or more. These are the (001), (011), and (111) Miller indices surfaces of fcc and bcc TMs, and the (0001), (101̅0), and (112̅0) of hcp TMs, following Miller-Bravais index notation for hcp metals, being a total number of 81 modeled surfaces.^32^ The simulation of extended surfaces has been performed using supercell slab models constructed from previously PBE optimized bulks,^34,35^ see Figure S2 in Section S1 of the Supporting Information for a depiction of the explored adsorption/absorption sites. The supercell size depends on the specific surface termination being modeled; (3×3) supercells composed of 54 atoms were used for fcc (111), hcp (101̅0), bcc (001), and bcc (111) surfaces, while (2×2) supercells composed of 48 atoms were used for fcc (001), fcc (011), hcp (0001), hcp (112̅0), and bcc (011) surfaces. All surface slab models contain six atomic layers, found to be enough to guarantee convergence with slab width of both energetic and physiochemical properties.^32^ The slabs contain nine atoms per layer for (3×3) slabs or eight atoms per layer for (2×2) slabs. Consequently, the adsorption/absorption of one C atom implies a similar coverage of ^1^/_9_ or ^1^/_8_ monolayers (MLs), respectively, defined as the number of C atoms with respect to the number of surface metal atoms on one side of the slab.

After optimization of the pristine surfaces, one C atom was adsorbed/absorbed with the three bottom layers of the slab fixed, while the other three upper layers were allowed to relax during the geometry optimization together with the interacting adsorbed/absorbed C atom, a procedure known as 3+3 approximation. The reciprocal space was sampled with a 3×3×1 **Γ**-centered Monkhorst Pack^36^**k**-points grid and calculations were performed using a Gaussian smearing of 0.2 eV energy width to speed up convergence, yet final energies were extrapolated to 0 K (no smearing).

The adsorption/absorption energies, *E*_ads/abs_ have been calculated as
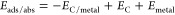
1where *E*_C/metal_ is the total energy of the metal slab with the C atom either adsorbed or absorbed, *E*_C_ is the energy of the isolated carbon atom in a vacuum, and *E*_metal_ is the energy of the optimized clean TM substrate. Within this definition, stable adsorption/absorption correspond to positive *E*_ads/abs_ values. Zero-point energies imply negligible variations between the stability of the different sampled sites, below 0.1 kJ·mol^–1^ according to test calculations, and so have been disregarded in the final analysis.

The surface, subsurface, sinking, and emerging *E*_b_, these are, the *E*_sur_, *E*_sub_, *E*_sink_, and *E*_emer_, respectively, were determined by using the climbing-image nudged elastic band (CI-NEB) procedure, using four images in between initial and final states.^37^ The approximate transition state (TS) geometries were posteriorly refined using a quasi-Newton optimization algorithm until forces acting on atoms were under 0.03 eV·Å^–1^. All TS were characterized by vibrational frequency analysis performed *via* construction and diagonalization of the corresponding block of the Hessian matrix, with elements estimated by analytical gradients from finite displacements of 0.03 Å length, certifying their saddle point nature with only one imaginary frequency. The *E*_b_ values were calculated by subtracting the TS energy from the initial diffusive energy state.

As far as the AI algorithms used in the analysis are concerned, the group analysis was carried out using the *k*-means (KM) approach, as implemented in the *sklearn* python library.^38^ The number of clusters for each case was determined using the elbow method,^39^ consisting in the evaluation of the cluster inertia (or distortion) curve shape—defined as the sum of squared distances of samples to their closest cluster center plotted against the number of clusters—and selecting the elbow of the curve, that is the minimum number of clusters that already yields the sought accuracy, and also by the silhouette scores method,^40^ accounting for the difference in between mean distances within a cluster and with the closest neighboring cluster. The optimal number of clusters resulting from both methods is then used in the cluster group descriptions, see more details in Section S2 of the Supporting Information. KM clustering was used to define subgroup types of TMs with similar activity with respect to C atoms, or similar diffusions. KM was also used to correlate these subgroups with the main descriptors defining these.

Concerning the tested ML regression algorithms, those were also implemented using the *sklearn* python library. Aside, *E*_ads/abs_ and *E*_b_ magnitudes were correlated with a list of TM features or physicochemical descriptors, commented in the forthcoming sections, using three different ML regression models: multivariable linear regression (MLR), decision tree regression (DTR), and random forest regression (RFR). Models were refined by removing unnecessary features using the leave-one-out procedure, and hyperparameters were tuned by conducting a grid search involving different parameter combinations and selecting the best-performing ones.

## 3 Results and Discussion

### 3.1 Thermodynamic Analysis

#### 3.1.1 Energy Landscape

**Figure 1:**
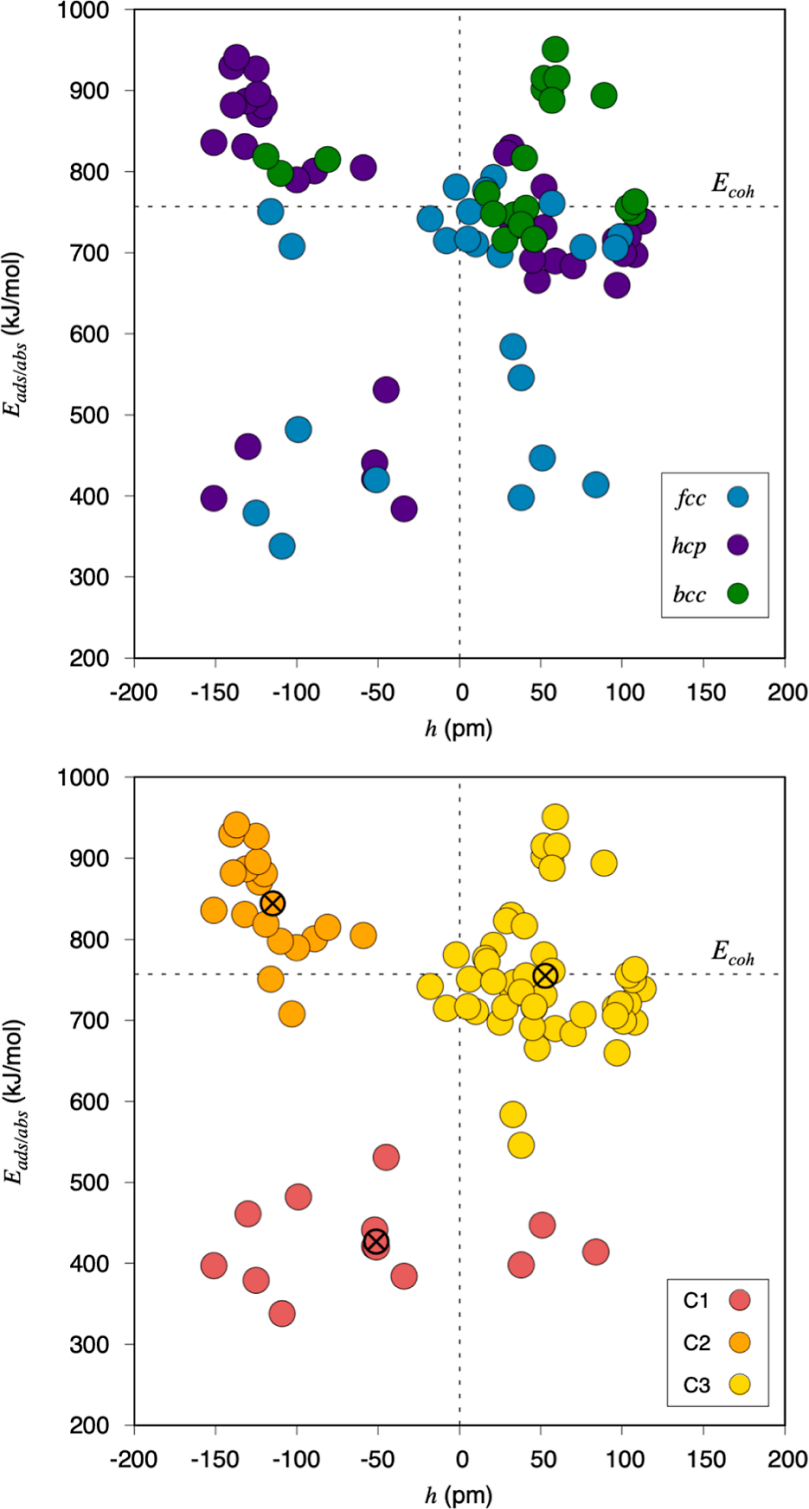
Most stable *E*_ads/abs_ situations on every TM surface with respect to *h*. In the top panel, values in blue correspond to fcc TMs, green to bcc TMs, and purple to hcp TMs. In the bottom panel, three different colors are used to mark the three different clusters determined through a KM analysis, with centers marked crossed.

Let us first start with an analysis of the thorough study of C interaction with the explored 81 TM surface models, so as to provide a general view of the C interaction with TMs. A full list of adsorption and absorption energies of the gained minima is present in Tables S1–S3 of Section S3 in the Supporting Information. For better readability, the top panel of Figure 1 shows a summarized overview of the results displaying the most stable position adsorption, *E*_ads_, or absorption, *E*_abs_, energy values for each surface termination of each metal—thus presenting three different values per metal— *versus* the C height, *h*, defined as the vertical distance between the C atom and the TM surface plane in each particular position, compiled in Tables S4–S6 of Section S3 of the Supporting Information. To have clear defined references, an in-plane situation is shown at zero *h*, along with the graphite cohesive energy, *E*_coh_, of 757 kJ·mol^–1^, obtained from the literature and obtained through equivalent optimizations as the ones here presented in terms of employed DFT exchange-correlation functional, plane-wave cutoff, **k**-points density, etc.^41^ As can be seen in Figure 1, the convenient utilization of *h* and *E*_coh_ references defines four different quadrants, which imply four different behaviors of C atoms when interacting with TM surfaces, depending on whether an adsorption or absorption situation is preferred, and whether the interaction of C with the TM is stronger or weaker compared to that in graphite.

A first analysis can be carried out with the data color-coded according to the TM CS; this is, fcc, hcp, or bcc. At first glimpse, one notices that nearly all bcc values are found to be above the surface level , implying preferential adsorption, except for three cases—V, Nb, and Ta(011) surfaces, all being in group V of the Periodic Table—where carbon prefers to lie subsurface. The *E*_ads/abs_ larger than *E*_coh_ implies that C has more affinity for bcc TMs than for other C atoms, a feature that happens in many cases, and in the above-commented subsurface cases, demonstrating a clear thermodynamic tendency for C to penetrate these metal surfaces. These results are indeed in perfect agreement with these metals propensity to form TMCs.^5^ However, because the majority of points imply surface positions, the carburization of bcc TMs, implying the interstitial placement of C, appears to be a nontrivial process. Notice that bcc CSs are perfect templates to place C in their octahedral interstitials, featuring the final rock salt CS of TMCs. Therefore, crystal reconstruction appears not to be a restricting aspect in many of these cases exhibiting rock salt TMCs, such as VC, NbC, TaC, CrC, δ-MoC, and FeC;^5^ although, for other more stable polymorphs, when necessary, the C incorporation is likely to involve a crystallographic phase transition. Previous studies on Zn oxidation and other TMs hydrogenation reflect a preferential subsurface stabilization of O and H atoms, including superficial crystallographic reconstructions,^42^ featured at higher atomic coverages, and so, such type of mechanisms could well explain the eventual subsurface incorporation of C to form TMCs, even when implying a crystal transformation.

Going beyond bcc TMs, fcc TM values are found in their majority in the lower part of the panel, that is adsorption or absorption energies weaker than in graphite, meaning that C has more clustering affinity than for these metals. This is in fair agreement with the known fact of fcc TMs being poisoned due to the formation of graphene/graphite layers on them, blocking their surface active sites. Nevertheless, this preference to form graphene layers may be interesting in the context of nanotechnology, for example in graphene synthesis from chemical vapor deposition or segregation.^21^ There are a couple of cases—Ni and Rh(001)—where the C interaction is above the graphite limit, which presents them as the least favorable for graphene synthesis, and more favorable concerning carbide formation, although their preference is on the surface, or in-plane. Note, however, that such strongly attached C can block still active sites or perturb the very chemical nature of nearby metal surface atoms. Finally, there are six fcc values that are clearly in the subsurface region: The two upper-most data points correspond to Ni and Pd(111), in agreement with previous calculations and experiments that certify the existence of carbide phases of these TMs.^19^ Moreover, the four lowest values correspond to Cu(111), Ag(111), Ag(011), and Au(011), indicating, as recently shown in the literature, that C has a tendency to penetrate subsurface on such noble metals.^18^ Notice that the ad/absorption strength and the surface/subsurface preference can be modulated in lower-coordinated sites, as found in fcc TMs NP edge regions,^18^ or even steps in vicinal surfaces, as a result of a higher malleability of the metal atoms, a feature probably present as well in other TMs with different CS.^17^

Last but not least, regarding hcp TMs, their values are scattered as is their position in the Periodic Table. Still, grouping is observed in the subsurface, highly attached C region, where C appears to present a tendency to penetrate such TMs. In fact, these values correspond to Sc, Y, Ti, Zr, Hf, Zn, and Cd. The first five belong to groups III and IV of the Periodic Table, known to display a very high chemical activity and a propensity to form rock salt TMCs. Thus, the high absorption energy could well contribute to compensating the energy demands for an eventual phase transition toward a rock salt structure. On the other hand, Zn and Cd are *d*^10^ elements, with an *a priori* low chemical activity according to *d*-band center arguments, see below. However, the strong interaction calculated here is in line with the existence of such carbides, as reported in previous studies.^43^

The data in Figure 1 has been used to carry out a KM clustering to better understand how this data can be grouped within the *E*_ads/abs_ and *h* two-dimensional space. Note that classification of different behavior in between the above-defined four quadrants is not sought by KM, but rather patterns of similar behavior in between TM surfaces. Thus, the found clusters do not necessarily have to be defined within the four-quadrant constraints. As shown in the bottom panel of Figure 1, the existence of three different clusters is best detected once the elbow or silhouette methods are applied, see Figures S4 and S5 of Section S4 of the Supporting Information. Data points in the first cluster, C1, have in common a rather weak interaction with the TM surfaces compared to the graphite *E*_coh_, with the group center or centroid—marked by a fictional crossed point in Figure 1—located at an *E*_abs_ of 424 kJ·mol^–1^, and with an *h* of −52 pm below the surface level. For these systems, C atoms would thermodynamically tend to merge into graphitic C aggregates on the surface, eventually poisoning the catalyst surface by site blocking. However, at low C concentrations, there could be C adatoms or interstitial C atoms, affecting the electronic structure of the surrounding metal atoms, particularly, when their mobility and eventual aggregation would be hindered, see below.

The second cluster, C2, is, on the contrary, characterized by a very strong interaction between C and the TM surfaces, and a general clear preference for the subsurface region, reflected in the centroid being located at an *E*_abs_ of 843 kJ·mol^–1^, and an *h* of −117 pm, implying that these TMs and surfaces are suitable for their carburization. Actually, C2 could be regarded as cases concentrated in the quadrant delimited by *E*_abs_ > 757 kJ·mol^–1^ and *h* < 0 pm, with the sole exception of Ni and Pd(111) surfaces, which feature an *E*_abs_ smaller than *E*_coh_, and so, similarly to C1 members, would imply an eventual formation of surface aggregated carbonaceous structures, particularly when kinetically allowed, in perfect agreement with the reported graphene growth by segregation reported on both surfaces.^44,45^

Finally, the third cluster, C3, groups most of the data points, where C atoms display an interaction with TM surfaces of similar strength to the cohesive energy of graphite, and a general clear preference for staying at the surface. This is reflected with the group center located at *E*_ads_ of 753 kJ·mol^–1^, very close to the *E*_coh_ of graphite of 757 kJ·mol^–1^, and a height as well of 53 pm. It is in such situations where the subtle imbalance of interaction strengths and kinetic movement inhibition may finally determine whether such C isolated moieties exist as such at low C concentrations or they eventually merge forming graphitic layers on the catalyst surface, and such imbalance could be potentially affected by the DFT uncertainty of *ca.* 20 kJ·mol^–1^. Even if this is a general trend, there are situations where C atoms have a significant affinity for TM surfaces, but now generally prefer to stay on the surface, thus occupying active sites that would no longer be available for any other reactants, acting as poisons by site blocking, exemplified by the subset of points with an *E*_ads_ larger than 850 kJ·mol^–1^. The exposed KM clustering allows defining certain general behaviors and could be tagged as cases with *h* > 0 pm, but this is not exempt from singularities; a couple of outliers can be caught from a visual inspection with an *E*_ads_ below a suited value of 600 kJ·mol^–1^, these are Cu(001) and Cu(011), whose behavior would be close to C1, although the inertia calculation assigns them to C3. Finally, three TM surfaces are placed with an *h* value slightly below 0, which are the Pd(001) and (011) surfaces, and the Pt(011) case, which would slightly enter the que quadrant of *E*_coh_ > 757 kJ·mol^–1^ and *h* < 0 where C1 cluster is mostly placed but with a significantly smaller subsurface height. Still, overall, one can generally claim that C1 lies below an *E*_ads/abs_ < 600 kJ·mol^–1^ limit, and C2 is inside the quadrant delimited by *E*_abs_ > 757 kJ·mol^–1^ and *h* < 0, while C3 features values of *h* > 0 and *E*_ads_ > 600 kJ·mol^–1^.

**Figure 2:**
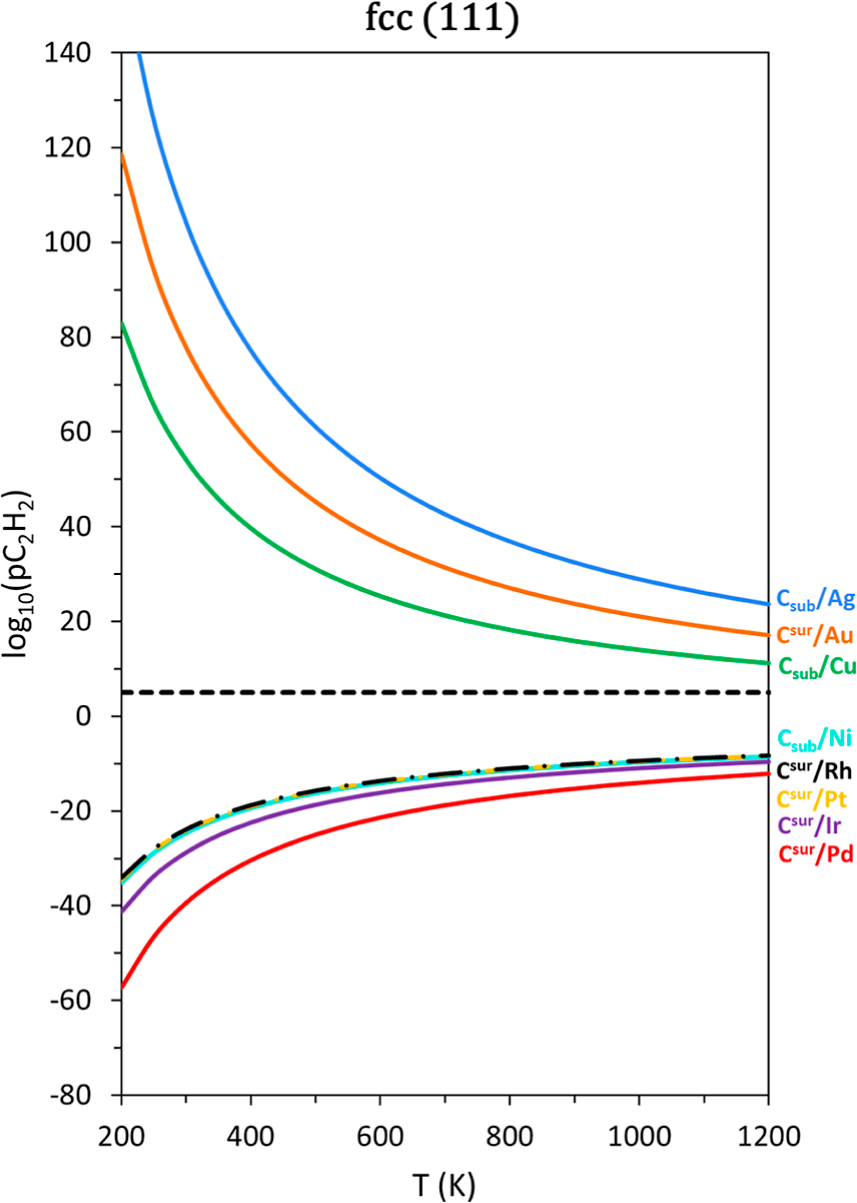
Phase diagrams for fcc TM (111) surfaces as a function of the acetylene partial pressure, *p*_C_2_H_2__, and temperature, *T*. Diagrams are obtained for a constant partial pressure of H_2_, *p*_H_2__ = 10^–7^ Pa. Regions above or below each curve indicate conditions at which the C-containing or pristine surfaces, respectively, are thermodynamically preferred.

Aside from the previous analysis, we inspected suitable reaction conditions at which such C moieties can be present on the inspected TM surfaces. To this end, thermodynamic phase diagrams have been built for the different studied TM surfaces, considering pressure and temperature working conditions that would turn pristine TM surfaces into early C-containing surfaces, either having C on the surface or subsurface. This has been carried out using acetylene (C_2_H_2_) as the carbon source, although other molecular sources could have been chosen. The reason for this is the first employment of such computational phase diagrams to evaluate the subsurface C presence during the acetylene hydrogenation on Pd catalysts,^45^ in order to correlate with experimental observations,^13^ and the use thereafter as a comparative playground.^18^ In these DFT-derived thermodynamic phase diagrams, the TM surface and molecular chemical potentials are equaled, see completed details on the phase diagram construction procedure found in the literature.^18,46,47^Figure 2 shows the exemplary phase diagrams corresponding to all fcc TM (111) surfaces because such TM surfaces are most relevant to Heterogeneous Catalysis, yet all other phase diagrams for fcc, hcp, and bcc surfaces can be found in Figures S6–S8 of Section S5 of the Supporting Information. Focusing on the cases revealed in Figure 2, the shown lines define, for each metal, working conditions of temperatures, *T*, and C_2_H_2_ partial pressures, *p*_C_2_H_2__, where C atoms adsorbed or absorbed would be thermodynamically equally stable to a pristine TM surface situation, see further details in the literature.^18^ Any *T* and *p*_C_2_H_2__ conditions above the shown curves imply a preference for having C adsorbed, C^sur^, or absorbed, C_sub_, while conditions below the curve imply a preferential TM pristine surface situation. In this particular case, C^sur^ moieties are expected for Pd, Ir, Rh, and Pt(111) surfaces, and C_sub_ for Ni(111) at, for example, a standard working *p*_C_2_H_2__ of 10^5^ Pa, and a regular catalytic working temperature of 600 K. Only Cu, Ag, and Au display their nobility in this aspect, Ag being the most C-resisting one, known and explained by the Ag deeper *d*-band center,^48^ joined to a weaker C–Ag coupling, which prevents antibonding states being above the Fermi level, eventually destabilizing the C interaction toward Ag.^49^ In any case, Cu and Ag(111) surfaces would prefer to incorporate such C moieties, while surface C would be observed on Au(111) surfaces.

The Group XI—Cu, Ag, and Au—nobility is also shown in (001) and (011) TM surfaces, see Figure S6 of Section S5 of the Supporting Information, revealing an enhancement of the surface chemical activity for (011) and (001) surfaces, the latter being the most chemically active, to the point of Cu(001) surfaces being C-poisoned at standard conditions of pressure and working temperatures above *ca.* 750 K. The rest of fcc TMs behave similarly among them, featuring systematically C^sur^ situations on (001) surfaces and most of (011) surfaces, with the exception of Ag, Au, and Pd(011) surfaces, where C_sub_ would be preferred.

When addressing hcp TMs, see Figure S7 of Section S5 of the Supporting Information, three different behaviors are found: Late TMs with *d*^10^ electronic configuration, these are, Group XII Zn and Cd, behave like noble Group XI elements, thus not being C-poisoned at standard working conditions. Other late TMs, like Group VII (Re and Tc), Group VIII (Ru and Os), and Group IX Co display a chemical affinity more similar to fcc Pt-group TMs, while early TMs such as Group III (Sc and Y) and Group IV (Ti, Zr, and Hf) show a high affinity toward C. As can be seen in Figure S6, regardless of the exposed surface, C_sub_ situations are preferred for the very early Groups III and IV TMs (Sc, Y, Ti, Zr, and Hf), and very late XII TMs (Zn, Cd), whereas C^sur^ situations are preferred on middle and late TMs of Groups VII (Re and Tc), VIII (Ru and Os), and IX (Co). The sole exceptions are Ti and Hf (101̅0) surfaces, where C^sur^ moieties are more stable. Finally, bcc TMs, belonging to early Groups V (V, Nb, and Ta), VI (Cr, Mo, and W), and VIII (Fe) reveal a high affinity toward C, see Figure S8 of Section S5 of the Supporting Information, going for a C-moiety presence in any working conditions, and only avoiding them at ultrahigh vacuum conditions and high temperatures. Such C moieties are systematically C^sur^ for (001) and (111) surfaces, and as well for (011) surfaces, with the exception of Group V TMs (V, Nb, and Ta), where C_sub_ is more stable, going for the C incorporation within the TM.

#### 3.1.2 Descriptors of the C Behavior at TM Surfaces

The abovementioned trends seem to point out that the chemical activity is somewhat influenced by the position along the *d* series of the Periodic Table. Thus, an important aspect resides in the search for descriptors of the ad/absorption energies accounting for the observed trends. Here, different descriptors proposed in the literature, either energetic or electronic, are evaluated so as to linearly correlate ad/absorption energies with them. In particular, surface energy, γ,^50^ work function, ϕ,^51^*d*-band center, ε_d_,^52^ corrected *d*-band center, ε_d_^W^,^53^ and the highest Hilbert transform *d*-band peak, ε_u_,^54^ are considered—details on their correct calculation can be found in the literature.^32,47^ Briefly, the description of their independent evaluations—see Figures S9–S13 in Section S6 of the Supporting Information for all the analyses details—reveals that the usually sought linear adjustment adequacy decreases in the order ε_d_ > ε_d_^W^ > ε_*u*_ > γ > ϕ. In the case of *d*-band center-based descriptors, it is clear that any attempt at improvement on ε_d_ is detrimental; still, the expected trend is captured, in the sense that the higher energy ε_d_, ε_d_^W^, or ε_u_, the stronger the C attachment. Likewise, the larger the surface energy, γ, the stronger the attachment energy of C; although the correlation on this energy-based descriptor is poor when compared to those based on the electronic structure. Finally, the work function, ϕ, is a very bad descriptor; not only because of the very small regression coefficient, *R*, of 0.17, but also because one would expect that the smaller the work function, the stronger the bonding, as a result of a TM → C charge transfer, observed in late TMs, and expected for earlier and more reducing TMs. However, the observed trend in Figure S13 of Section S6 of the Supporting Information is just the opposite.

**Figure 3:**
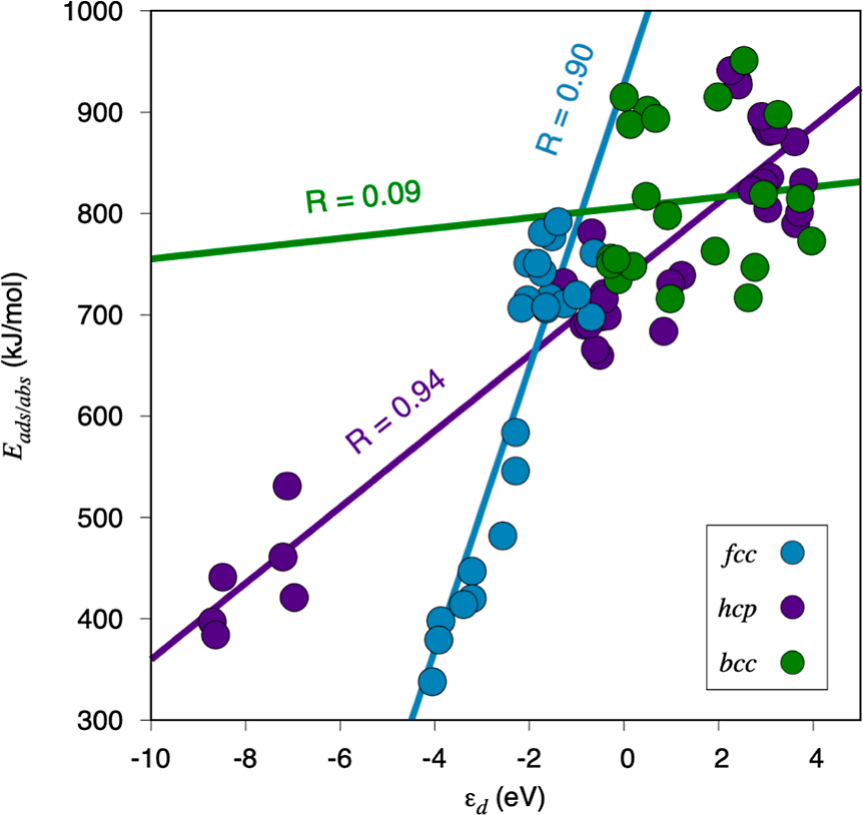
Most stable *E*_ads/abs_ situations with respect to ε_d_, and the corresponding linear correlations. Values in blue correspond to fcc TMs, green to bcc TMs, and purple to hcp TMs.

Given the abovementioned analysis, ε_d_ could be regarded as the most successful descriptor, at least, when describing the exhibited thermodynamic data. The most stable *E*_ads/abs_ versus ε_d_ results are shown in Figure 3 grouping results as per the different featured CSs. Notice that an evaluation with one regression line for each type of surface termination, shown in Figure S14 of Section S6 of the Supporting Information, reveals that the trends for the three surfaces of each crystallographic have similar slopes, and such slopes are very different for the different examined crystal structures. These results reinforce the consideration of crystal packing as a determining aspect concerning the C interaction with TM surfaces. Focusing on Figure 3, however, one readily notices that linear trends can only be valid for hcp and fcc structures, which present regression coefficients, *R*, of 0.94 and 0.90 respectively. On the other hand, the bcc TMs trend line is not representative, presenting a poor regression coefficient of solely 0.09; showing that there is no correlation in these cases. This puts the accent in that the ε_d_ descriptor, typically tested on coinage and Pt-group TMs,^51^ all featuring fcc and hcp close-packed situations, does not account for a possible crystallographic effect that can translate into changes in the TM surface chemical activity. Indeed, hcp and fcc TMs differ in the stacking of their close-packed layers, but their trends seem to be independent, see Figure 3, implying a certain stacking effect. Another possible source of dispersion of the bcc points is that DFT accuracy is known to be lower when describing virtual, unoccupied orbitals. This affects the early TMs more substantially, which is reflected in larger errors for the bcc TMs, mostly located in Groups V (V, Nb, and Ta) and VI (Cr, Mo, and W) of the Periodic Table, and to a lesser extent to hcp TMs because includes TMs from Groups III (Sc and Y) and IV (Ti, Zr, and Hf), whose larger dispersion is observable for points with ε_d_ > 0 eV in Figure 5. Actually, discarding such points in hcp TMs only improves the fit, as seen in Figure S15 of Section S6 of the Supporting Information. Finally, note that bcc TMs are known to feature a different density of states compared to fcc or hcp TMs, with a bimodal shape with a dip near the Fermi level,^55,56^ as a consequence of a major role of *s* and *p* orbitals in the metal bonding.^57^ Thus, the bcc TMs shape is significantly different from fcc and hcp ones, a feature not captured with the *d*-band center, making the application of the d-band model on bcc not as suited as on fcc and hcp TMs and probably requiring higher d-band moment descriptors.

**Figure 4:**
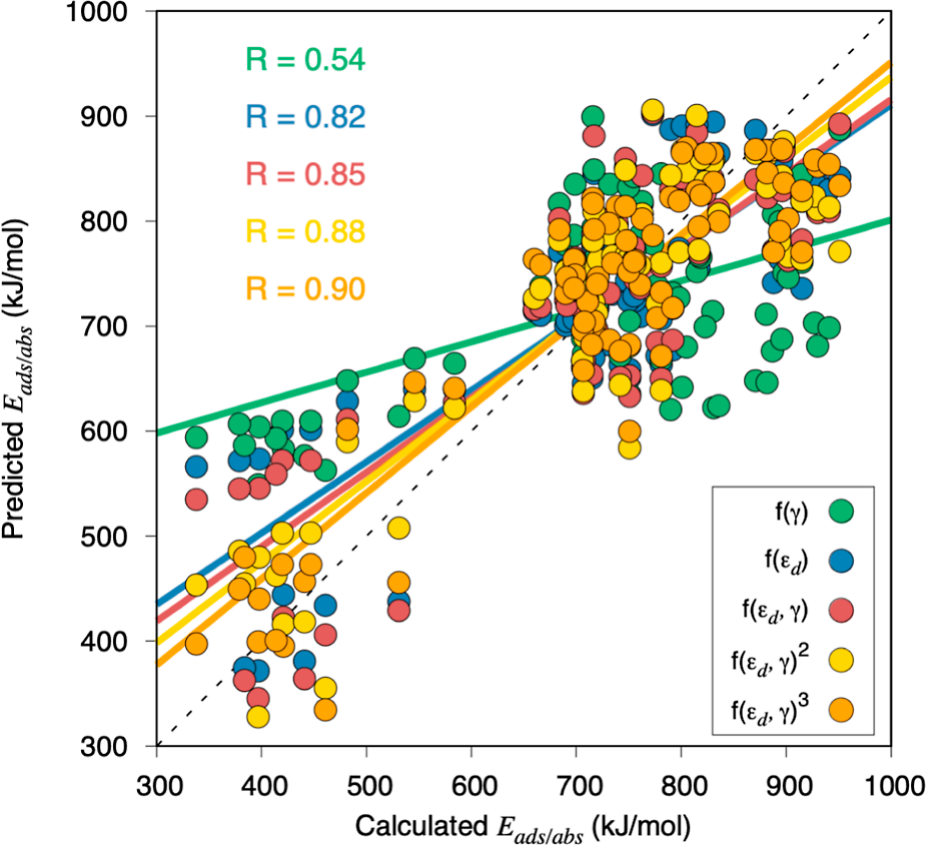
Most stable *E*_ads/abs_ situations vs the predicted *E*_ads/abs_ from different linear, multivariable, and polynomial regressions involving γ, ε_d_, or a combination of them. The black dashed line represents a perfect correlation.

Still, the trends based on energetic and electronic structure descriptors, for example on ε_d_ and γ, pave the way to inspect whether there exists combined effects of them, something that could be revealed by using the sure independence screening and sparsifying operator^58^ but tackled here by evaluating them through multivariable regressions, still maintaining a linear response for each influencing descriptor yet increasing the model complexity. As shown in Figure 4, combining ε_d_ with γ leads to a better correlation than using them alone. Furthermore, one could make combinations of a larger degree, for example, having γ·ε_d_ terms or even squared values for each descriptor, which would be second-degree combinations or even third-degree combinations including, for example, γ^2^·ε_d_ or ε_d_^3^ terms. By considering these, one observes a gradual improvement in the multivariable adjustment, as observed by an increase of the *R* value up to 0.90, and the approach of the adjustment to the ideal one. These observations strongly suggest that, when looking for adsorption and/or absorption energy descriptors, one should not restrain from finding the perfect one, as it may well not exist, as the interaction is simultaneously influenced by different surface properties. Thus, one should look for combinations of descriptors, each of them bringing a different piece of information about the metal surface one works with.

The abovementioned analysis indeed laid the foundations for a proper and deeper multi-variable analysis, feasible by applying different AI machine learning (ML) regression methodologies. To this end, the *E*_ads_ or *E*_abs_ values of most stable positions are expressed as a function of the above-introduced descriptors (ε_d_, ε_d_^W^, ε_*u*_, γ, and ϕ), but regarding as well the CS of the TM case, given the different behaviors of fcc, hcp, and bcc TMs and including as well the surface coordination number (CN) retrieved from the literature,^59^ to differentiate the different studied surfaces for the same metal, plus the bulk shortest interatomic distance, δ,^34,35^ as a geometry measure distinct for every TM, even when having the same CS and CN. Aside, the number of TM d electrons, *n*_d_, are accounted for, as they represent a natural trend across the d series. Finally, to represent the different adsorption or absorption sites, the number of TM atoms neighboring the C atom is defined, CN_site_, allowing for site specificity. This accounts for a total of ten descriptors related to the TM surface. Note that, because the same adsorbing or absorbing moiety is regarded always, that is, C atoms, no descriptors from the ad/absorbed species are necessary.^60^

Within this set of descriptors and features, we evaluated three different ML algorithms, including MLR, DTR, and RFR, see details of them in Section S7 of the Supporting Information, and explicative images of the DTR procedure in Figures S16 and S17 in Section S7 of the Supporting Information. Briefly, MLR is analyzed to evaluate the adequacy of the combination of descriptor linear responses, while DTR and RFR are utilized to move out from linear effects, but, more importantly, to find out the main, key descriptors, and their weight in estimating ad/absorption energies. A first analysis, using all the aforementioned features and default parameters of the algorithms—for example, 100 trees in RTR, and a maximal number of allowed features to be used in each tree to be equal to the total number of considered features—provided by the *sklearn* python library was performed using a shuffle split cross validation (CV) employing 20 splits for the set of the 81 surfaces with C in its most stable position. For the CV, for each size of the training set, a number of random data points are taken which represent 80% of the data set. The remaining 20% are random points also taken to be used for the test set.

The analysis results, in terms of mean absolute error (MAE) ± standard deviation, yielded test set values of 66.7 ± 12.5, 49.1 ± 8.4, and 43.5 ± 8.4 kJ·mol^–1^ for MLR, DTR, and RFR, respectively, for the largest training set size. Thus, focusing on RFR, the regression algorithm that delivered the smaller error, one can successively remove those features that were less relevant in terms of minimizing the test set MAE. To this end, we used the leave-one-out procedure, which consists in removing one feature at a time and assessing the impact on the test set MAE to decide whether it is worth including or not. In this case, three parameters emerge as most relevant from the analysis; not surprisingly, ε_d_ and γ, as outlined above, but also the number of metal atoms neighboring the adsorbed or absorbed C, that is the site coordination, CN_site_, which brings site-specificity to the analysis.

**Figure 5:**
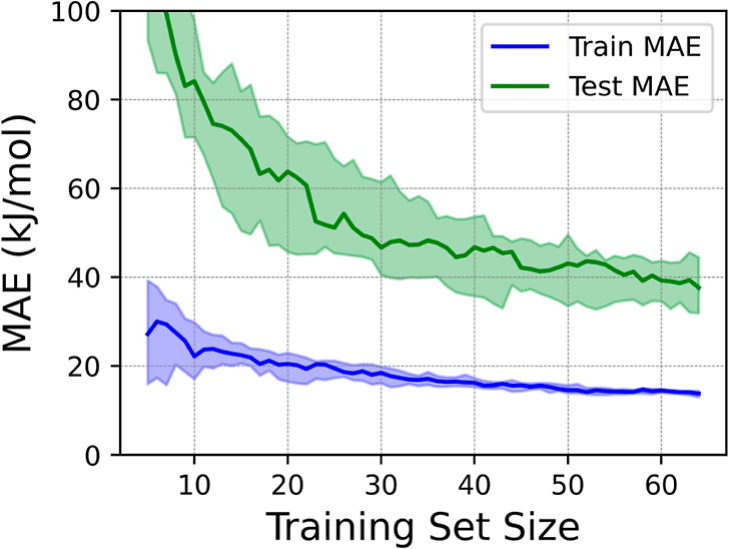
MAE evolution for training (blue) and the test (green) sets as a function of the number of samples contained in the training set for the prediction of *E*_ads/abs_ using an RFR algorithm. Colored areas around the lines account for the error dispersion resulting from the CV using 20 runs.

Once the candidate descriptors are shortlisted according to the leave-one-out approach, the RFR algorithm hyperparameters were tuned by performing CV evaluations on different combinations of parameters and selecting those that minimized the MAE, exemplified in Figure 5 by the learning curve of RFR, displaying the training and test errors when increasing the number of samples in the training set. Results showed that, by using ε_d_, γ, and CN_site_ only as input features, an RFR with 50 decision trees considering two features for each split already provided a very good training set MAE of 13.7 ± 0.9 kJ·mol^–1^, although the test set MAE accuracy is of 39.1 ± 8.9 kJ·mol^–1^, clearly better than previous approaches combining genetic algorithms descriptor selection with MLR, reaching accuracies of the order of ∼100 kJ·mol^–1^.^61^ Notice that curves are not fully converged, and lower MAEs could be expected by widening and diversifying the number of cases, using, for example, vicinal surfaces or sites at NP models, or even when differentiating adsorption from absorption situations. The built and improvable RFR tool model is freely accessible on GitHub.^62^

Notice that the obtained final accuracy for the test set is still far from using such an ML model to carry out forecasts in a quantitative fashion, where, desirably, one would require MAEs below the DFT accuracy, estimated to be *ca.* 20 kJ·mol^–1^. However, still, it can be useful for a rapid evaluation and qualitative assessment. Further than that, the most interesting factor is that, from the RFR algorithm, one can seize the importance of each key feature. In this case, the important factors are 0.6, 0.3, and 0.1 for ε_d_, γ, and CN_site_, respectively, quite in line with the abovementioned discussion referring to ε_d_ as the main descriptor, but weighting the importance as well of γ, as already above-detected in the descriptor analysis, where combinations of them were found to improve the regression quality, see Figure 4. Still, their definition is somewhat modulated by the site coordination, a feature not identified in the previous analysis. A note of caution must be driven, though, when trying to use such a combination of descriptors for stepped, vicinal surfaces, as different and inequivalent surface metal atoms are present, which translate into different ε_d_ and CN_site_ values; even if a single γ value can be estimated for such vicinal surfaces, its splitting into different facet contributions plus step edge energies could be better suited for descriptor management,^63^ yielding to more accurate ad/absorption energy estimates. The use of NP models may also incorporate inherent intricacies as well, such as NP and site features.^64^

**Figure 6:**
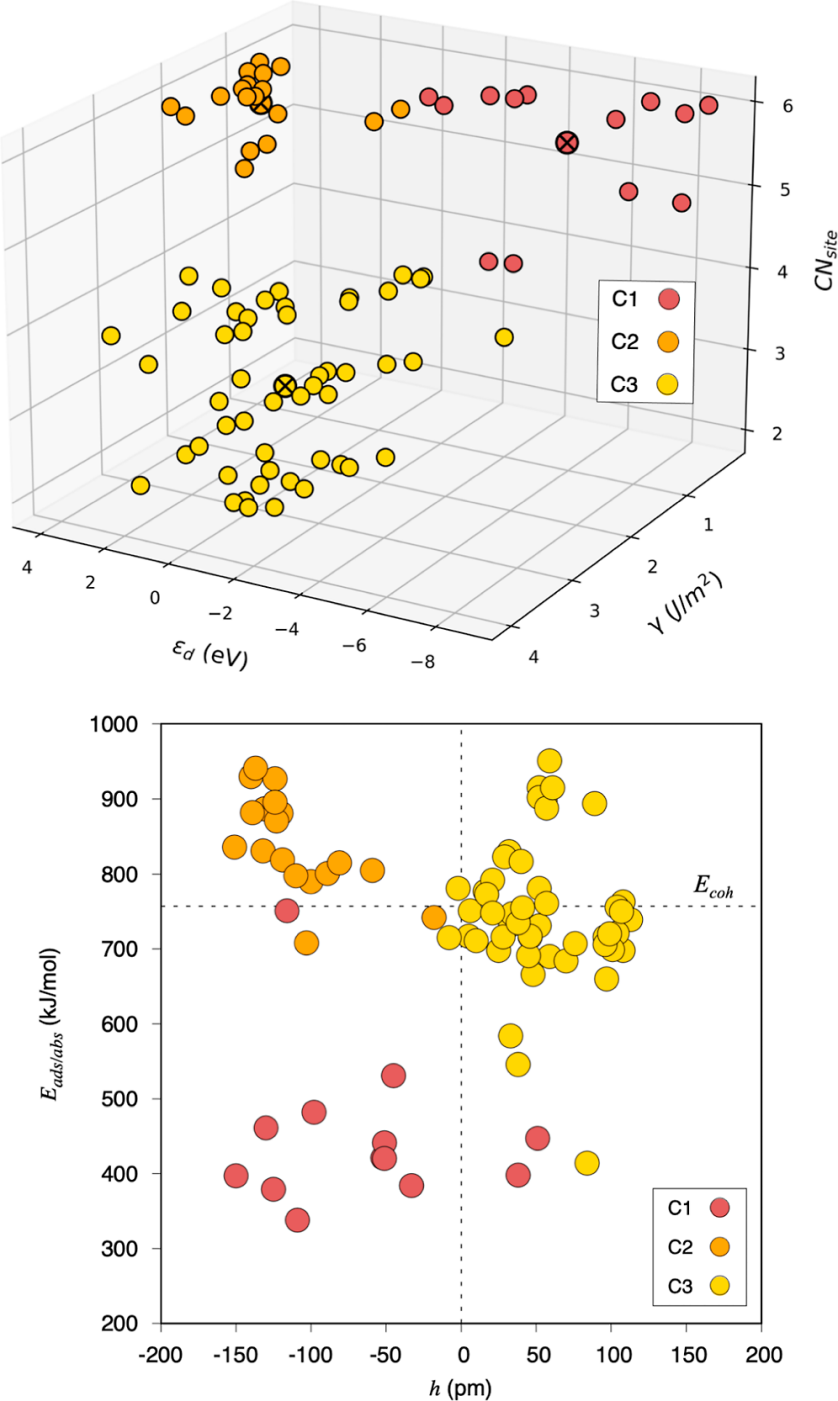
Top panel: The three-dimensional KM clustering of the most important features extracted from the RFR, namely ε_d_, γ, and CN_site_. Bottom panel: A quadrant plot as in Figure 1, but showing the clusters from the top panel projected in the *E*_ads/abs_/*h* space.

After having isolated the main physicochemical descriptors through the RFR method, one could well carry out a KM analysis as done in Figure 1, but now identifying similarities in the descriptor space instead of on the target *E*_ads/abs_ and *h* properties. This three-dimensional grouping is shown in Figure 6 (top panel). The components of these three clusters have been projected into the *E*_ads/abs_/*h* space in Figure 6 (bottom panel). Surprisingly, one observes that clusters in *E*_ads/abs_/*h* space, shown in Figure 1, mostly coincide with gained clusters in descriptors space, justifying the grouping displayed in Figure 1, with only a few exceptions. Pd(111) surface of C1, appearing in C2; the Pd(011) surface of C2, appearing in C3; and the Au(111) surface of C3, appearing in C1. In any case, the resulting grouping underscores the fact that groups of systems with similar *E*_ads/abs_ and *h* also exhibit similar descriptor values. From the shown values in Table 1, one notes that, even when accounting for the standard deviation, the feature average values mostly do not overlap with each other for the different clusters, indicating that their representation is mostly unique, which reinforces that such descriptor values are key in defining the features groups shown in Figure 1.

**Table 1:** Average Values for ε_d_, γ, and CN, for Each of the Clusters Shown in Figure 6 and Their Standard Deviation

	ε_d_/eV	γ/J·m^–2^	CN_site_
C1	–0.1 ± 1.7	2.6 ± 0.7	3.0 ± 0.9
C2	–5.4 ± 2.5	0.7 ± 0.3	5.5 ± 0.8
C3	2.5 ± 1.7	1.6 ± 0.5	5.8 ± 0.4

### 3.2 Influence of Kinetics

#### 3.2.1 Energy Barriers Landscape

The abovementioned analysis dealt only with the energetic stability and, therefore, to reach a holistic overview one would require analyzing the C moieties diffusion kinetics on all the studied TMs, as, for example, the subsurface presence may be kinetically hindered when the sinking energy barrier, *E*_sink_, is too large, and the same applies to the possible C emergence for *E*_emer_, hindering an eventual surface coke formation from subsurface C atoms. Aside, coke formation could be as well hindered by surface diffusion, governed by the *E*_sur_ kinetic energy barrier, *E*_b_. Finally, we evaluate here the possible diffusion at the subsurface level, defined by *E*_sub_, and question whether lateral diffusion preferentially happens on the surface. Notice that such barrier information has been found valuable in a catalytic context, for example, it serves to obtain the mean lifetimes of such species, and so, in which time frame they can affect the surface ongoing catalyzed process, as recently demonstrated on fcc (111) surfaces by kinetic Monte Carlo simulations.^20^

The four different types of diffusion barriers, illustrated in Figure S3 of Section S1 of the Supporting Information, have been obtained by the CI-NEB algorithm and properly characterized by vibrational analysis. For each TM type of surface, different diffusive paths have been investigated, including nontrivial ones for certain complicated diffusions. The explored paths are listed in Tables S7–S9 of Section S8 of the Supporting Information. For each case, the lowest *E*_b_ values have been collected, accounting for a total of 324 diffusion energy barriers, summarized along their diffusion path in Table S10 of Section S8 of the Supporting Information. Notice that the mean values, including standard deviation, reveal, as expected, *E*_sur_ diffusion energy barriers of 86.7 ± 55.8 kJ·mol^–1^, being slightly lower than *E*_sub_, of 94.5 ± 63.1 kJ·mol^–1^. Still, the difference is not excessively large, and already at this stage, one could envisage that surface and subsurface diffusions are similarly possible. This striking finding can be easily explained, as, on one hand, surface diffusion TSs get stabilized thanks to the more freedom of flexibility of surface metal atoms; however, subsurface diffusion TSs get stabilized thanks to the higher metal coordination, see Figures S18–S20 of Section S8 of the Supporting Information. Other than that, sinking energy barriers, *E*_sink_, have a sensibly larger value of 117.1 ± 79.0 kJ·mol^–1^, while emerging energy barriers, *E*_emer_, are noticeably smaller, of the order of 57.9 ± 56.3 kJ·mol^–1^, succinctly implying that it is more difficult for C adatoms to dissolve in the metal matrix than to segregate toward the surface.

Still, the large standard deviation of the points expresses a great variety of situations. For instance, the largest *E*_sur_ of 263.2 kJ·mol^–1^ corresponds to the Ta(001) surface, where such C atoms would be rather immobile, at variance with Cu(111), where, with an *E*_sur_ of 8.4 kJ·mol^–1^, C atoms would be rather mobile; a point that favors the observed graphene synthesis by deposition on such surfaces.^21,65^ Even if subsurface diffusion is less favored and quite inhibited, for example on W(111) with an *E*_sub_ of 303.7 kJ·mol^–1^, it is rather easy on the Zn(101̅0) surface, with an *E*_sub_ of 2.4 kJ·mol^–1^ only. Similarly, one can think that C sinking into the subsurface region is quite difficult, and it is indeed on W(001), with an *E*_sink_ of 326.6 kJ·mol^–1^, while C sinking through the Zn(112̅0) surface is essentially barrierless, with an *E*_sink_ of 0.8 kJ·mol^–1^. Finally, C emergence to the surface is rather easy on the Co(101̅0) surface, with an *E*_emer_ of 0.8 kJ·mol^–1^, while it can be quite difficult on Ta(011), where C segregation would be rather impeded with an *E*_emer_ of 229.0 kJ·mol^–1^.

**Figure 7:**
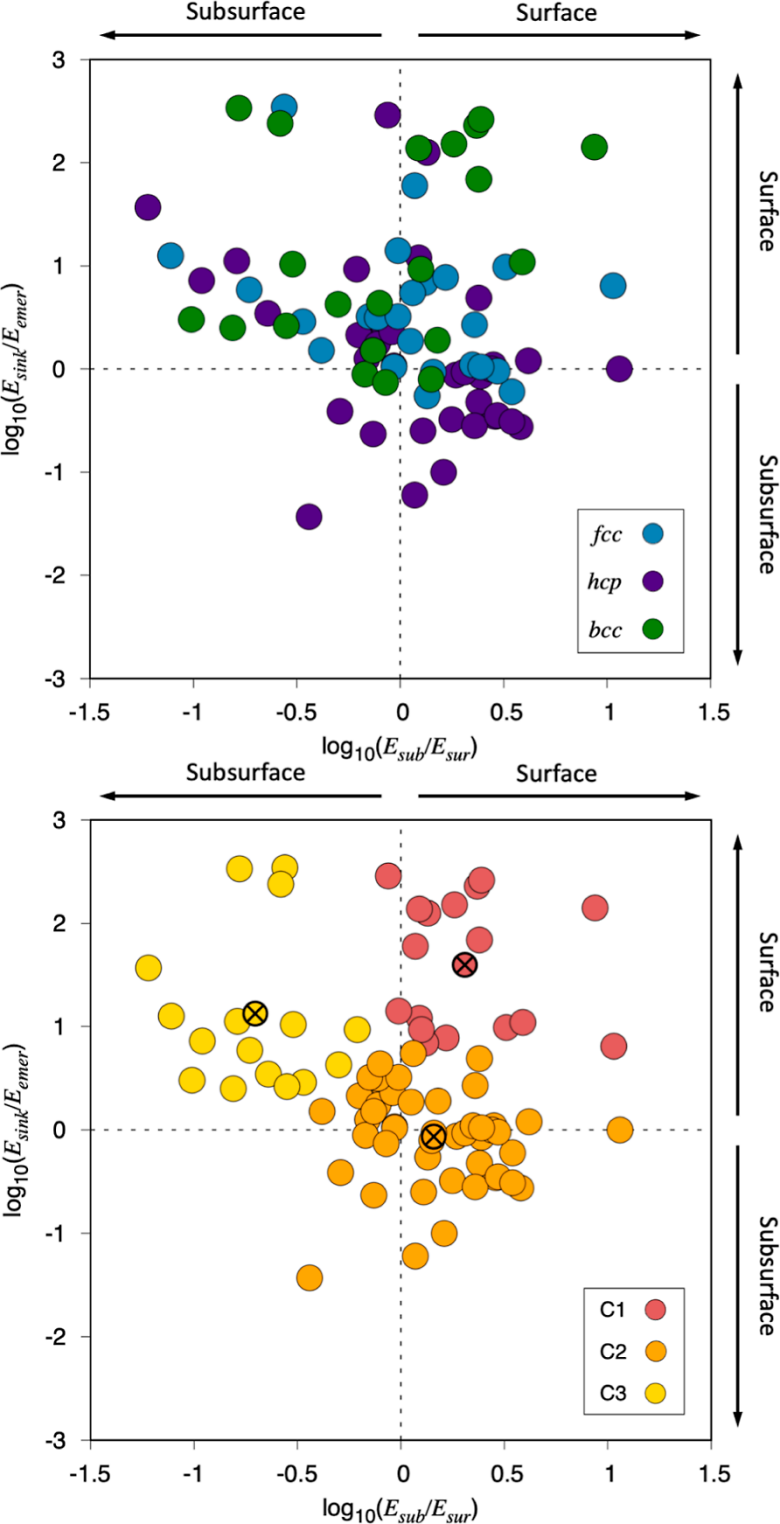
The base 10 logarithm of *E*_sub_/*E*_sur_ with respect to the base 10 logarithm of *E*_sink_/*E*_emer_. In the top panel, values in blue correspond to fcc TMs, green to bcc TMs, and purple to hcp TMs. In the bottom panel, three different colors are used to mark off the three different clusters determined through a KM analysis.

This large set of data, which can be quite different, can also be gathered and analyzed in a four-quadrant plot, in a similar fashion as done for the thermodynamics in Figure 1. To do so, Figure 7 shows the log_10_(*E*_sub_/*E*_sur_) versus log_10_(*E*_sink_/*E*_emer_), having thus 81 points corresponding to the same amount of explored TM surfaces. Thus, zero value for log_10_(*E*_sub_/*E*_sur_) implies that *E*_sub_ = *E*_sur_, and so, surface and subsurface diffusion are kinetically equally feasible. Likewise, zero value for log_10_(*E*_sink_/*E*_emer_) implies that surface C sinking diffusion is kinetically as likely as the subsurface C emerging. These two zero values delimit the four quadrants in Figure 7. Values larger than zero for log_10_(*E*_sub_/*E*_sur_) imply that *E*_sub_ values are higher than *E*_sur_, and hence, for these cases, C atoms would diffuse more easily along the surface than through the slab subsurface region, points located on the right side of the zero limit. The opposite behavior is expected for log_10_(*E*_sub_/*E*_sur_) smaller than zero, located on the left side. When it concerns log_10_(*E*_sink_/*E*_emer_) values, data above the zero limit imply that emerging is preferred over sinking, which is a preferential location on the surface. On the contrary, values below zero imply a preference for C penetrating the subsurface region.

The top panel of Figure 7 features data colored depending on the CS of the TM, revealing that fcc and bcc TMs data points behave similarly, being dispersed on the top half of the plot, implying that for such metals *E*_sink_ is often larger than *E*_emer_, and going for a kinetically allowed surface presence of C adatoms. Still, a few cases are the early bottom part of the plot. These belong to fcc (111) Ni, Pd, Pt, Cu, Ag, and Au(111) surface models, where the abovementioned situation reverses, implying a more difficult segregation toward the surface of dissolved C atoms in the bulk metal matrix. Notice that the (111) surface termination is the most stable and abundant one on such TM metals,^32,55^ and so such subsurface C effect should not be discarded on any ongoing surface catalyzed process. Aside, neither fcc nor bcc TMs show a clear preference between *E*_sur_ and *E*_sub_, with every metal being somehow unique in this regard. Finally, hcp TMs show sizable dispersion but are mainly found in the bottom right part of the quadrant plot. This quadrant gathers situations with an *E*_sink_ barrier lower than *E*_emer_, and *E*_sub_ being larger than *E*_sur_. Hence, one observes that most hcp TMs feature kinetically favorable penetration of isolated C atoms toward the subsurface region, but with restricted diffusion along the subsurface, as the diffusion across the surface is preferred.

Furthermore, the bottom panel shows the KM analysis again using three clusters as derived from Figure 1. There, it is clear that C1 fits values of the upper-right quadrant, implying *E*_emer_ barriers smaller than *E*_sink_, and *E*_sur_ is smaller than *E*_sub_. Altogether, these features imply a certain preference for C atoms to be located on the surface, and diffusing over it, as shown by the cluster center, where *E*_sub_ is two times larger than *E*_sur_, and *E*_sink_ is 39.8 times larger than *E*_emer_. Beyond that, C3 also nicely fits one quadrant, now implying an easier C emergence compared to the sinking, but, more importantly, featuring *E*_sub_ values sensibly smaller than *E*_sur_ ones, as shown by the cluster center, located at a point where *E*_sur_ values are five times larger than *E*_sub_, and *E*_sink_ values are 12.8 times larger than *E*_emer_. This quadrant points out certain TM surfaces where C diffuses more easily going through the subsurface region, probably due to the aforementioned stabilization of the diffusion TS states by higher coordination. In this sense, C moieties would move around in the subsurface region and only emerge from time to time, as if they were the targets of a whack-a-mole game. Finally, C2 contains the most numerous group, displaying, in general, well-balanced *E*_sub_/*E*_sur_ and *E*_sink_/*E*_emer_ ratios near unity, as shown by the center with the ratios of 1.4 and 0.9, respectively. Only a certain preference is found for a surface diffusion over the subsurface one, but, aside from that, the general behavior would be that all diffusion processes should be regarded feasible, implying somewhat free mobility on the surface, through the subsurface region, and with the exchange of C atoms in between surface and subsurface sites.

#### 3.2.2 Descriptors Controlling the Energy Barriers

Similar to the analysis carried out for the adsorption and absorption energies, the attention is now placed on finding descriptors of the diffusion energy barriers. The ones used for the thermodynamic minima are ε_d_, ε_d_^W^, ε_*u*_, γ, and ϕ, which are listed in Figures S21–S25 of Section S9 of the Supporting Information. There, even when using different regressions for each barrier type, the obtained results were unsatisfactory, reaching, at most, a regression coefficient *R* of 0.69 for *E*_sink_ versus γ. Still, the limited applicability of such descriptors can be thought of as natural, as such descriptors were developed and applied to seize the interaction strength, not the heights of energy barriers. Still, trends are regularly observed, that is, the larger the ε_d_, ε_d_^W^, or ε_u_ the larger the *E*_b_, and so it applies for γ and ϕ, although these inverse trends are observed for *E*_emer_ and *E*_sub_, respectively.

**Figure 8:**
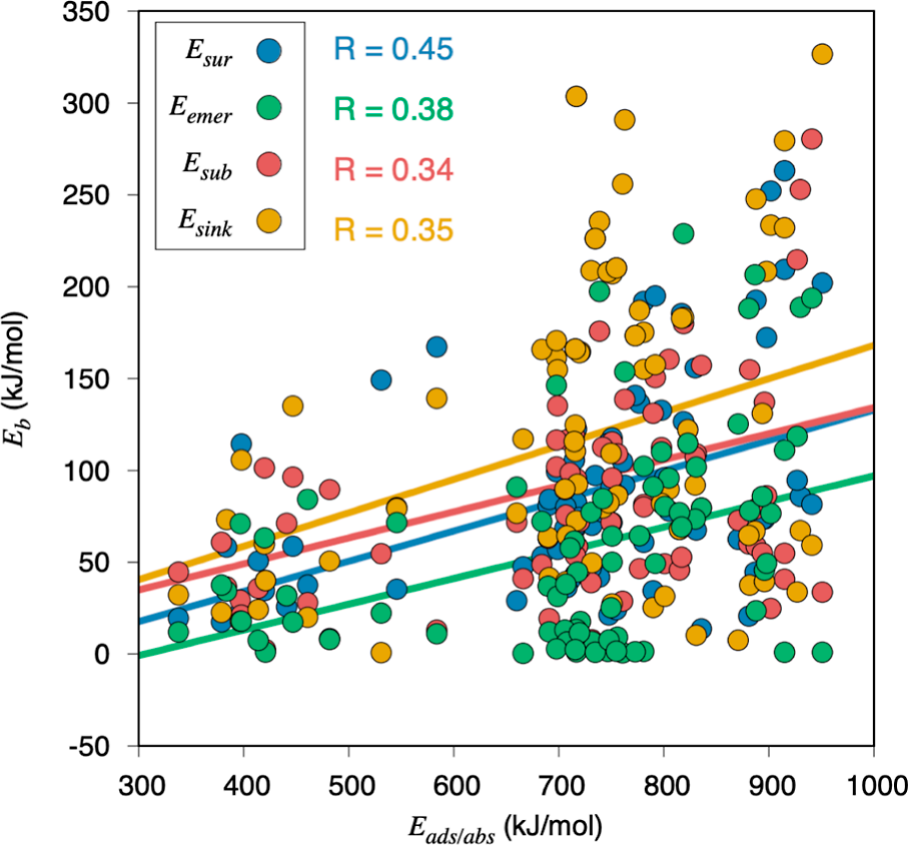
Linear evolutions of the different *E*_b_ diffusion energy barriers—*E*_sur_, *E*_sub_, *E*_sink_, and *E*_emer_—with respect to the most stable initial position *E*_ads_ or *E*_abs_.

In any case, the previous discussion underscores the point of similar trends achieved for adsorption or absorption energies and diffusion energy barriers. Within this context, one has to remark on the work of Nilekar et al.,^66^ who showed in their seminal work that the diffusion energy barrier depends on the adsorption strength of the adsorbed moiety, being twelve percent of the latter, with an *R* of 0.85. Figure 8 shows this trend for the four different diffusion processes here studied, revealing that, indeed, the stronger the adsorption or absorption energy, the larger the diffusion energy barrier, and in all the cases with similar slopes of *ca.* 0.16. However, the regression coefficients, *R*, are still quite modest, being at most 0.45 for *E*_sur_ energy barriers.

**Figure 9:**
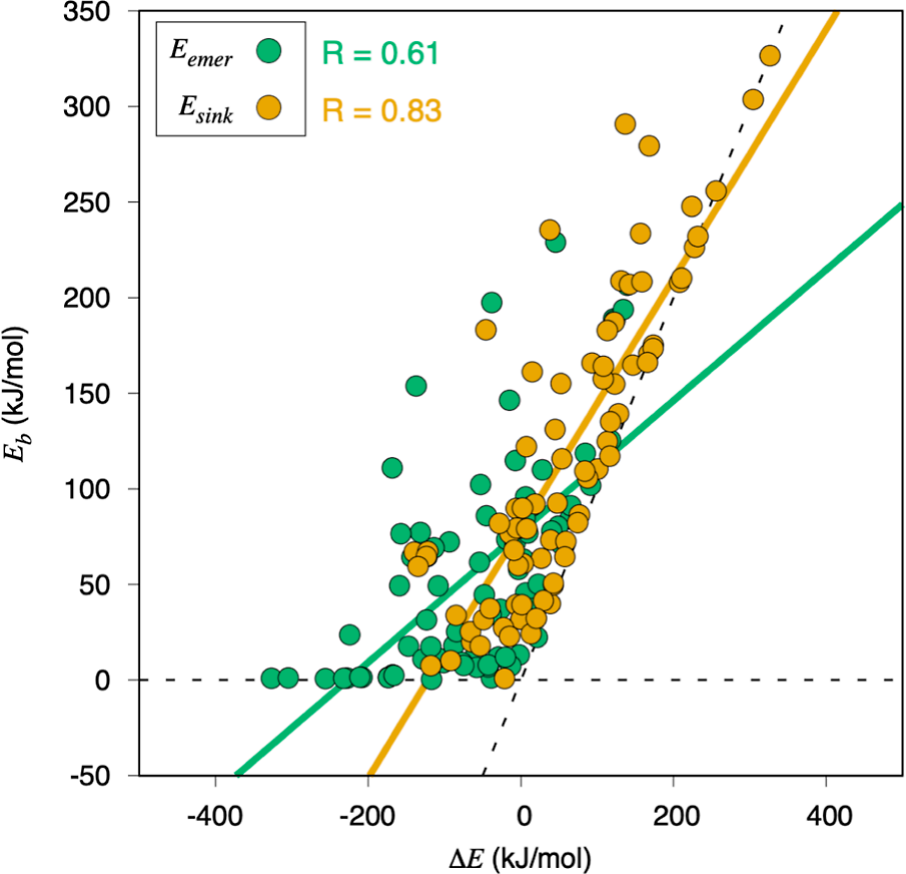
BEP linear evolutions of *E*_b_ with respect to Δ*E*, and the corresponding linear correlations for each explored barrier type. Dashed lines define the latest TS limit, where *E*_b_ = Δ*E*, or the earliest TS limit, where *E*_b_ = 0 regardless of Δ*E*.

Another extended linear correlation used for energy barriers is Brønsted–Evans–Polanyi (BEP) relations, linearly expressing a reaction step energy barrier with the step energy variation, Δ*E*.^67−69^ In this work, we do not deal with reaction steps, as we are focusing on diffusion processes, but the same principle applies. To this end, the BEP relations were analyzed, yet only for C sinking and emerging processes, as surface and subsurface diffusions feature a Δ*E* of zero. Figure 9 shows their BEP analysis with a fairly good correlation for *E*_sink_, with a regression coefficient *R* of 0.83; however, the regression for *E*_emer_ shows more dispersion, with a lower R of 0.63. Notice that for the sinking process, the linear regression is close to the limit of a very late TS, where *E*_b_ = Δ*E*, signaled in Figure 9 with a dashed black line. Another regression constrain is the earliest TS situation, where *E*_b_ equals zero regardless of the value of Δ*E*. Still, there is also a number of cases located in between, so no evident trend can be claimed. At variance with the description of *E*_ads/abs_ with respect to ε_d_, the BEP correlation coefficients when splitting the data into different crystallographic groups do not substantially improve the outcome, indicating this time that the crystal structure is not a determining factor in the BEP correlations for either *E*_sink_ or *E*_emer_; see Figures S26 and S27 in Section S9 of the Supporting Information.

**Figure 10:**
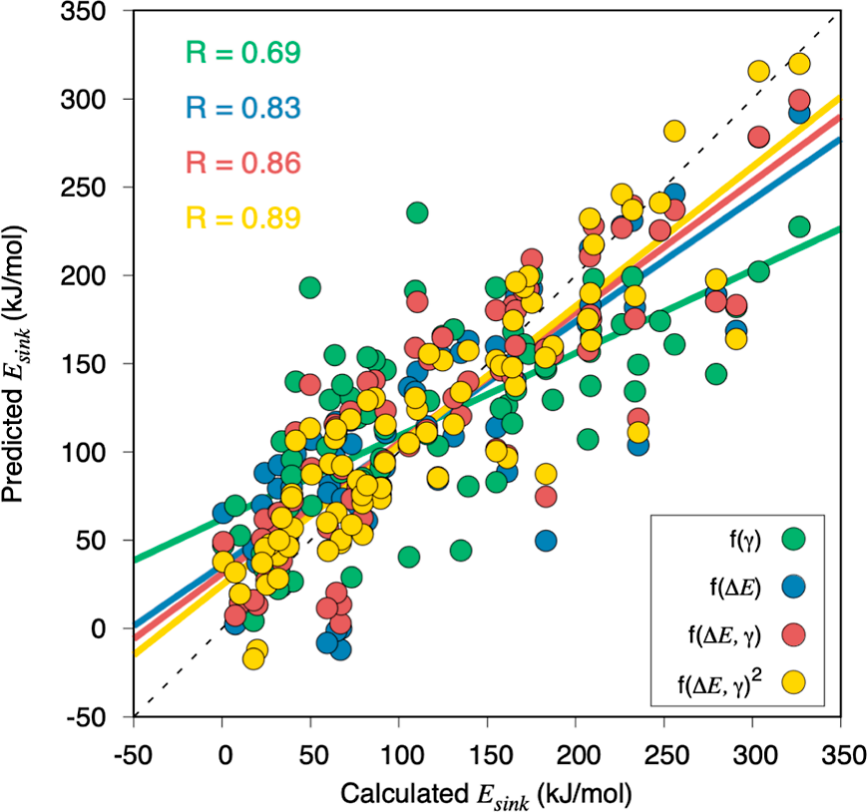
Calculated *E*_sink_ vs the predicted *E*_sink_ from different linear, multivariable, and polynomial regressions involving γ, Δ*E*, or different degrees of combinations of them. The black dotted line represents the ideal correlation.

Mimicking the abovementioned analysis of *E*_ads_ or *E*_abs_ dependence on ε_d_ and γ, and given the best correlations observed for *E*_sink_ as described by γ and Δ*E*, we carried out a multivariable regression involving combinations of Δ*E* and γ descriptors up to a second-order degree—because third-order degree yielded no improvement—shown in Figure 10. There, as happened in the thermodynamic evaluation in Figure 4, one observes that (i) the combination of both factors is beneficial, with an improved *R* of 0.86, while (ii) the incorporation of descriptors of higher orders translates into a mild improvement. As happened with the thermodynamic analysis, this underscores that the description of kinetic processes should be tackled by considering a combination of different descriptors, instead of looking for a single, determining one, as appears that different aspects influence the kinetic energy barriers.

Thus, following the same procedure as for the thermodynamic analysis, we applied here different ML regression algorithms to have tools to forecast diffusion energy barriers and locate key descriptors governing such processes. Note that now the data set is four times larger—324*E*_b_ values—than the 81 cases of *E*_ads_ or *E*_abs_. The features and descriptors used are the same as the thermodynamic analysis, but now adding the most stable position *E*_ads/abs_ and Δ*E*. Moreover, the barrier type, BT—*E*_sur_, *E*_sub_, *E*_sink_, or *E*_emer_—and the CN of the initial and final sites, named CN_IS_ and CN_FS_, respectively, are also included. The target goal here is to predict *E*_b_, and to this end, MLR, DTR, and RFR have been used. A first analysis was carried out following the procedure and hyperparameters used for the thermodynamic data, yielding MAEs of 34.3 ± 2.4, 42.5 ± 4.7, and 33.1 ± 2.8 kJ·mol^–1^ for MLR, DTR, and RFR, respectively. Again, RFR is posed as the best-performing ML algorithm.

By refining the number of descriptors by means of the leave-one-out procedure, the less relevant features were removed, and left only those that had a significant enough impact in terms of error minimization, which are Δ*E*, *E*_ads/abs_, ϕ, CN_FS_, and CN_IS_, with weights of 0.43, 0.27, 0.15, 0.09, and 0.06, respectively. Notice how Δ*E* is the most important feature, as expected from the BEP analysis and particularly true for *E*_sink_ and *E*_emer_ barriers, although it is not as predominant as ε_d_ was for *E*_ads/abs_. The next in the list is indeed the most stable position *E*_ads/abs_, in line with the discussion above where it was found to affect the barrier heights. Notice that the two most important descriptors are thermodynamic parameters, which can be used to estimate kinetic energy barriers. Other factors affecting the energy barriers are, unexpectedly, ϕ, even if by scratch does not show good linear correlations with the kinetic data, and CN_IS_ and CN_FS_, which shows that the CN of the sites involved in the diffusion play minor, yet a relevant role in the height of the barriers.

**Figure 11:**
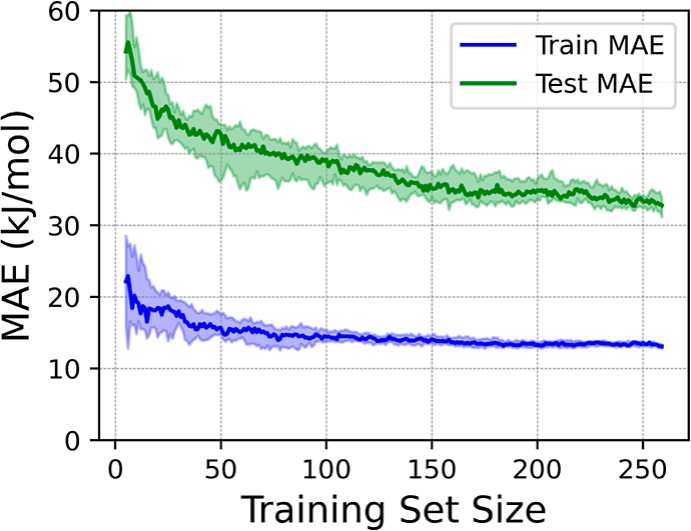
MAE evolution for training (blue) and the test (green) sets as a function of the number of samples contained in the training set for the prediction of *E*_b_ using an RFR algorithm. Colored areas around the lines account for the error dispersion resulting from the CV using 20 runs.

As the last step, we selected the best performing hyperparameters of the RFR algorithm by performing CV evaluations on different combinations of parameters and choosing those that lead to minimal MAE, see the learning curve in Figure 11. Results showed that, by using only Δ*E*, *E*_ads/abs_, ϕ, CN_FS_, and CN_IS_ as the input features, an RFR with 30 decision trees that considers three features for each split, see Figure 11, provided the best accuracy, with an MAE of 32.6 ± 2.3 kJ·mol^–1^ for the test set, while the training set offers an MAE of 13.1 ± 0.4 kJ·mol^–1^. The dimensionality reduction in Figure 7 avoids using a KM clustering in the feature space, also because different features are simultaneously affecting the different *E*_b_ values. Other than that, the analysis done in Figure 11 can actually be carried out, differentiating the four different diffusive processes. This has been done and discussed in Figures S28–S31 and Table S11 of Section S10 of the Supporting Information. Briefly, RFR persisted as the best performing algorithm, with MAEs very similar to that obtained when considering all *E*_b_ altogether, with the best performance found for *E*_sur_ barriers, with a test set MAE of 25.2 ± 6.6 kJ·mol^–1^, and the less accurate case being *E*_sub_, with a test set MAE of 38.2 ± 7.1 kJ·mol^–1^. Compared to the thermodynamic data, the RFR offers more accuracy for diffusion energy barriers, which gets closer to the typical DFT accuracy of 10–20 kJ·mol^–1^, although one is still far from achieving an accuracy that would support quantitative estimations. However, the main descriptors affecting the C diffusions have been unfolded, and estimates can be argued upon them, allowing for a rapid qualitative assessment and sieving process.

## 4 Conclusions

In this work, by performing high-throughput periodic DFT calculations on proper slab models of 81 TM surfaces with the maximum Miller index of one using the PBE *xc* functional, we provide compelling thermodynamic *plus* kinetic information on the interplay of the C atom on and in such TM surfaces, providing detailed information on adsorption and absorption minima sites and energies, and diffusive energy barriers along the surface region, subsurface region, as well as in between both regions, summarized in Table S12 of Section S11 of the Supporting Information. The provided thermodynamic and kinetic results are in line with experimental observations regarding carbide formation in bcc and early hcp TMs, also revealing the possibility of surface reconstruction, being the key step of such process in some of the cases. Moreover, results are also in line with the well-known tendency of C to form graphene layers on top of fcc TM surfaces, effectively poisoning them for heterogeneous catalysis purposes. Thermodynamic phase diagrams have been built for all the explored surfaces, delimiting temperature and ethylene partial pressure working conditions at which the presence of C atoms would be favorable.

Further than that, electronic and energetic descriptors have been analyzed on ad- and absorptive minima and energy barriers, where the *d*-band center has been found to be the most successful one when correlating ad/absorption energies, in a particular reliable fashion for hcp and fcc TMs, whereas presenting significant deviations for bcc TMs. For diffusion energy barriers, the adsorption or absorption strength of the departing minimum, and the difference in energy between minima, Δ*E*, in the cases of sinking and emerging diffusions, have been used. Finally, *k*-means clustering has been used to delimit three types of TM thermodynamic and kinetic behavior toward C atoms, while ML random forest regression revealed that, both for the gained thermodynamic and kinetic data, a combination of descriptors yields a better description of minima and energy barriers, although the accuracy is so far only valid for a rapid qualitative assessment. In any case, the analysis underscores the need of looking for a few descriptors biasing the interaction strength and diffusion possibilities, rather than trying to get a single perfect descriptor, which may not be the wisest option as different physicochemical aspects do indeed affect such processes.

## Supporting Information Available

Models and definitions, *k*-means clustering details, ad/absorption energies and heights, *k*-means inertia and silhouette scores evaluation, built phase diagrams, evaluation based on isolated descriptors, details on the employed machine-learning regression algorithms, diffusion energy barriers and their evaluation based on single descriptors and their evaluation based on ML regressions, and a table delivering a summary of C adsorption/absorption preferences (PDF)
