# Supplementary material for: Charting the Atomic C Interaction with Transition Metal Surfaces
Source: ACS Catal. 2022 Jul 15;12(15):9256–69. doi: 10.1021/acscatal.2c01562 (PMC9880994; doi:10.1021/acscatal.2c01562)
Supplement: Supplementary file 1 — cs2c01562_si_001.pdf [file cs2c01562_si_001.pdf]

Charting the Atomic C Interaction with Transition Metal Surfaces

Oriol Piqué,[†,¶] Iskra Z. Koleva,[‡,¶] Albert Bruix,[†] Francesc Viñes,[†,*] Hristiyan A. Aleksandrov,[‡,*] Georgi N. Vayssilov,[‡] and Francesc Illas[†]

[†] *Departament de Ciència de Materials i Química Física & Institut de Química Teòrica i Computacional (IQTCUB), Universitat de Barcelona, c/ Martí i Franquès 1, 08028 Barcelona, Spain*

[‡] *Faculty of Chemistry and Pharmacy, University of Sofia, 1126 Sofia, Bulgaria*

*Emails: francesc.vines@ub.edu and haa@chem.uni-sofia.bg

Index of Sections

S1. Models and Definitions S2
S2. K-means Clustering S5
S3. Ad/Absorption Energies and Heights S6
S4. K-means Inertia S16
S5. Phase Diagrams S18
S6. Isolated Descriptors Evaluation S21
S7. Machine Learning Regression Algorithms S28
S8. Diffusion Energy Barriers S32
S9. Diffusion Energy Barriers Isolated Descriptors S44
S10. Machine Learning Algorithms on Diffusion Energy Barriers S51
S11. Summary Table S56
References S58

[¶] Both authors contributed equally

Section S1: Models and Definitions

Figure S1. Periodic table with *fcc* TMs coloured in blue, *bcc* TMs in green, and *hcp* TMs in purple.

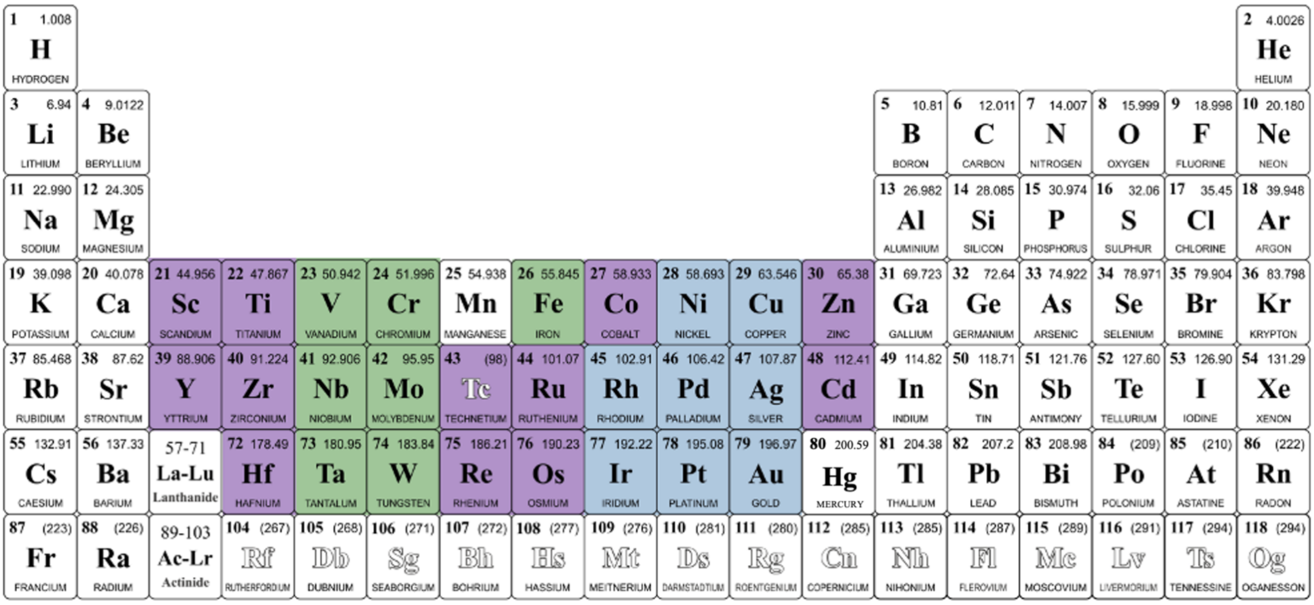

Figure S2. Depiction of positions studied for each surface termination of each crystallographic structure. In blue, from left to right, *fcc* unit cell, and (001), (011), and (111) surfaces. In green, from left to right, *bcc* unit cell, and (001), (011), and (111) surfaces. In purple, from left to right, *hcp* unit cell, and (0001), $(10\bar{1}0)$, and $(11\bar{2}0)$ surfaces. Positions depicted for *fcc* (001) correspond to a) Top, b) Hollow, c) Bridge; for *fcc* (011) a) Top, b) Hollow, c) BridgeS, d) BridgeL; for *fcc* (111) a) Top, b) Hollow *hcp*, c) Hollow *fcc*, d) Bridge. Positions depicted for *bcc* (001) correspond to a) Top, b) Hollow, c) Bridge; for *bcc* (011) a) Top, b) Hollow, c) HollowT, d) Bridge; for *bcc* (111) a) Top, b) Hollow *hcp*, c) Hollow *fcc*, d) Bridge. Positions depicted for *hcp* (0001) correspond to a) Top, b) Hollow, c) HollowE, d) Bridge; for *hcp* (100) a) Top, b) Hollow, c) BridgeL, d) BridgeS; for *hcp* (110) a) Top, b) Hollow, c) BridgeL, d) BridgeS.

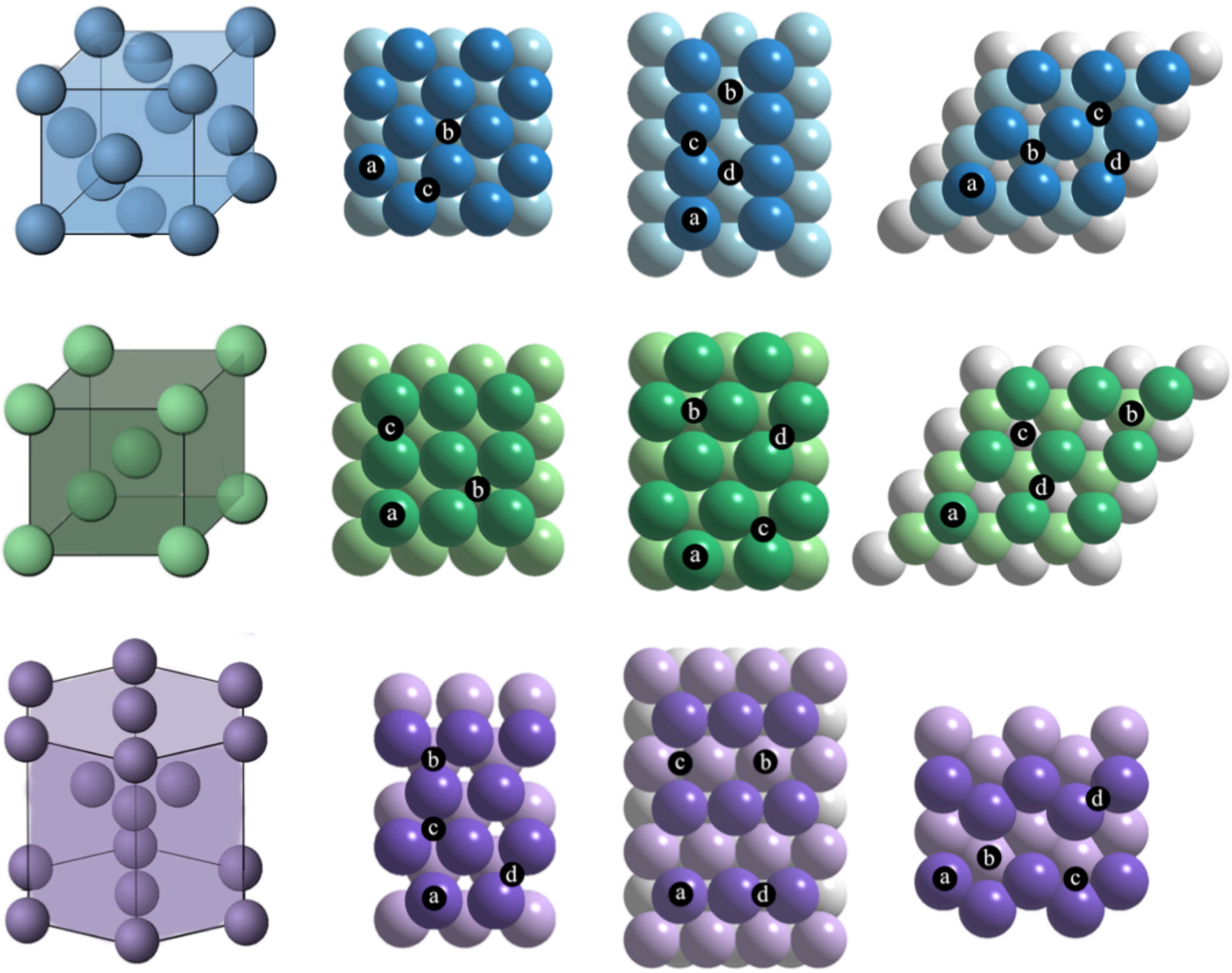

Figure S3. Schematic depiction of the different diffusion barrier types studied in this work: Surface diffusion (E_{sur} – blue), subsurface diffusion (E_{sub} – red), sinking diffusion (E_{sink} – yellow), and emerging diffusion (E_{emer} – green).

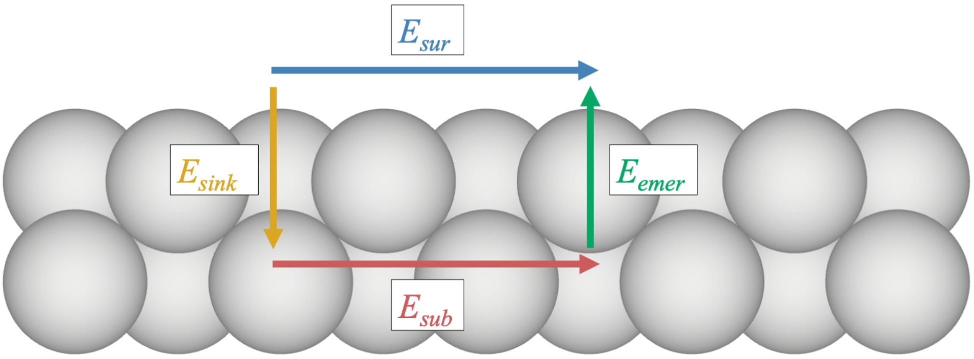

Section S2: K-means Clustering

The k-means (KM) algorithm has been used as implemented in the *sklearn* python library.[1] KM clusters data by splitting samples in n groups (clusters) of equal variance, where n is defined beforehand. The algorithm automatically finds n cluster centres which minimize the within-cluster sum-of-squares, also called inertia, see Eq. 1, in order to avoid compensation by negative displacement vectors from the centre, and as a way of preferentially gather those data points close to the cluster centre, and, at the same time, bias those located farther away.

$$\sum_{i=0}^{n} \min_{\mu_j \in C}\left(\left\|x_i - \mu_j\right\|\right)^2 \quad (1),$$

where μ_j is the samples mean, also called cluster centroid, in the C disjoint clusters.

Inertia can be understood as a quantitative measure of how coherent clusters are, yet has some drawbacks. It assumes that clusters are isotropic and convex, and so, performs poorly with elongated clusters. Moreover, note that inertia is a non-normalized metric; *i.e.* one can just state that lower values are better, and zero optimal. In that regard, it does not perform well in high-dimensional spaces, where Euclidean distances tend to become inflated. Running a dimensionality reduction algorithm prior to the k-means analysis can help alleviating this problem.

However, that is not the case in the present study, and so this method is suited for the employed data, displayed two-dimensionally. Note that there exist other methodologies to find and define groups of similar behaviour, such as the subgroup discovery (SGD),[2] where a target variable is expressed as a function of a series of description features, which are Monte Carlo optimized to define regions of subgroups, as done *e.g.* in the past in the categorization of binary structures to rocksalt or zinc blende structures as a function of the atomic radii of valence s and p orbitals.[3] However, SGD defines fringe conditions from which one expects one or another behaviour, while here KM defines centroids whose close data implies a similar behaviour.

Focusing on KM, notice that when n is exactly the number of data points, the minimum inertia of zero is achieved. Therefore, one needs to define a criterion to restrict to a small and useful n number. This has been done using the elbow method,[4] which consists in plotting the inertia as a function of n and picking the elbow of the curve as the suited number of clusters to use. Further than that, we employed the silhouette scores,[5] simply defined as b-a/max(a,b), where a is the mean distance between points of a group, b is the mean distance of such points to the closest neighbouring cluster, and max(a,b) is the largest value within such sets. This way, the most gathered and cohesive clusters without overlapping each other maximize this function, which can be used to determine the optimum number of clusters for the KM.

Section S3: Ad/Absorption Energies and Heights

Table S1. Adsorption, E_{ads}, and absorption, E_{abs}, energies of all different positions studied for (001), (011), and (111) surfaces of *fcc* TMs. All values are given in kJ mol^{-1}.

TM	(001) Position	E_{ads}	E_{abs}	(011) Position	E_{ads}	E_{abs}	(111) Position	E_{ads}	E_{abs}
**Rh**	Top	513	654	Top	523	560	Top	498	631
	Hollow	777	—	Hollow	633	—	Hollow *hcp*	706	612
	Bridge	638	613	BridgeS	651	658	Hollow *fcc*	686	684
	—	—	—	BridgeL	717	—	Bridge	—	—
**Ir**	Top	543	505	Top	577	412	Top	534	555
	Hollow	761	—	Hollow	600	—	Hollow *hcp*	720	559
	Bridge	656	527	BridgeS	697	530	Hollow *fcc*	697	588
	—	—	—	BridgeL	698	—	Bridge	—	—
**Ni**	Top	432	684	Top	423	610	Top	424	605
	Hollow	792	—	Hollow	697	—	Hollow *hcp*	651	593
	Bridge	582	612	BridgeS	578	708	Hollow *fcc*	647	708
	—	—	—	BridgeL	711	—	Bridge	—	—
**Pd**	Top	428	728	Top	438	646	Top	423	685
	Hollow	—	781	Hollow	685	—	Hollow *hcp*	682	705
	Bridge	599	695	BridgeS	613	748	Hollow *fcc*	680	751
	—	—	—	BridgeL	—	742	Bridge	—	—
**Pt**	Top	501	608	Top	531	542	Top	479	650
	Hollow	751	—	Hollow	616	—	Hollow *hcp*	697	696
	Bridge	642	677	BridgeS	662	661	Hollow *fcc*	707	652
	—	—	—	BridgeL	—	715	Bridge	—	—
**Cu**	Top	292	455	Top	266	459	Top	256	383
	Hollow	584	—	Hollow	546	438	Hollow *hcp*	436	394
	Bridge	419	442	BridgeS	—	538	Hollow *fcc*	440	482
	—	—	—	BridgeL	528	—	Bridge	—	—

**Ag**	Top	176	310	Top	170	317	Top	161	268
	Hollow	398	—	Hollow	364	—	Hollow *hcp*	314	294
	Bridge	250	311	BridgeS	—	379	Hollow *fcc*	320	338
	—	—	—	BridgeL	364	—	Bridge	—	—
**Au**	Top	238	329	Top	246	314	Top	211	310
	Hollow	447	—	Hollow	401	—	Hollow *hcp*	403	389
	Bridge	391	413	BridgeS	395	420	Hollow *fcc*	414	350
	—	—	—	BridgeL	—	417	Bridge	—	—

Table S2. Adsorption, E_{ads}, and absorption, E_{abs}, energies of all different positions studied for (001), (011), and (111) surfaces of *bcc* TMs. All values are given in kJ mol^{-1}.

TM	(001) Position	E_{ads}	E_{abs}	(011) Position	E_{ads}	E_{abs}	(111) Position	E_{ads}	E_{abs}
**V**	Top	431	—	Top	478	738	Top	364	599
	Hollow	898	—	Hollow	806	—	Hollow *hcp*	636	—
	Bridge	722	769	HollowT	—	815	Hollow *fcc*	773	—
	—	—	—	Bridge	769	—	Bridge	770	—
**Nb**	Top	436	—	Top	446	722	Top	353	550
	Hollow	902	—	Hollow	770	—	Hollow *hcp*	526	—
	Bridge	645	740	HollowT	—	798	Hollow *fcc*	636	—
	—	—	—	Bridge	636	—	Bridge	716	—
**Ta**	Top	404	—	Top	438	696	Top	373	539
	Hollow	915	—	Hollow	773	—	Hollow *hcp*	556	—
	Bridge	640	740	HollowT	—	819	Hollow *fcc*	619	—
	—	—	—	Bridge	645	—	Bridge	747	—
**Cr**	Top	540	—	Top	487	629	Top	421	544
	Hollow	915	—	Hollow	750	—	Hollow *hcp*	637	—
	Bridge	683	777	HollowT	—	666	Hollow *fcc*	729	—
	—	—	—	Bridge	659	—	Bridge	755	—
**Mo**	Top	512	—	Top	477	566	Top	450	508
	Hollow	888	—	Hollow	756	—	Hollow *hcp*	537	—
	Bridge	684	749	HollowT	—	679	Hollow *fcc*	620	—
	—	—	—	Bridge	668	—	Bridge	735	—
**W**	Top	522	—	Top	447	496	Top	416	412
	Hollow	951	—	Hollow	763	—	Hollow *hcp*	499	—
	Bridge	736	796	HollowT	—	626	Hollow *fcc*	576	—
	—	—	—	Bridge	672	—	Bridge	717	—
**Fe**	Top	526	—	Top	691	823	Top	495	673
	Hollow	817	—	Hollow	894	770	Hollow *hcp*	505	—
	Bridge	696	692	HollowT	—	849	Hollow *fcc*	728	—
	—	—	—	Bridge	821	—	Bridge	748	—

Table S3. Adsorption, E_{ads}, and absorption, E_{abs}, energies of all different positions studied for (0001), $(10\bar{1}0)$, and $(11\bar{2}0)$ surfaces of *hcp* TMs. All values are given in kJ mol^{-1}.

TM	(0001) Position	E_{ads}	E_{abs}	$(10\bar{1}0)$ Position	E_{ads}	E_{abs}	$(11\bar{2}0)$ Position	E_{ads}	E_{abs}
**Sc**	Top	—	739	Top	—	688	Top	—	794
	Hollow	753	—	Hollow	665	—	Hollow	841	—
	HollowE	—	871	BridgeS	—	801	BridgeL	—	—
	Bridge	—	—	BridgeL	752	—	BridgeS	—	882
**Y**	Top	—	736	Top	—	670	Top	—	755
	Hollow	739	—	Hollow	616	—	Hollow	781	—
	HollowE	—	831	BridgeS	—	790	BridgeL	—	—
	Bridge	—	---	BridgeL	724	—	BridgeS	—	836
**Ti**	Top	—	702	Top	—	705	Top	—	787
	Hollow	779	—	Hollow	688	—	Hollow	843	—
	HollowE	757	881	BridgeS	—	812	BridgeL	—	—
	Bridge	—	—	BridgeL	830	—	BridgeS	—	927
**Zr**	Top	—	728	Top	380	—	Top	—	792
	Hollow	776	—	Hollow	583	—	Hollow	808	—
	HollowE	747	887	BridgeS	—	805	BridgeL	—	—
	Bridge	—	—	BridgeL	799	—	BridgeS	—	930
**Hf**	Top	—	713	Top	382	636	Top	—	767
	Hollow	790	—	Hollow	517	—	Hollow	806	—
	HollowE	756	896	BridgeS	—	816	BridgeL	—	—
	Bridge	—	—	BridgeL	823	—	BridgeS	—	941
**Tc**	Top	468	—	Top	594	527	Top	—	626
	Hollow	698	567	Hollow	571	—	Hollow	690	—
	HollowE	684	669	BridgeS	751	608	BridgeL	—	—
	Bridge	—	—	BridgeL	781	—	BridgeS	607	664
**Re**	Top	490	525	Top	515	—	Top	—	591
	Hollow	732	—	Hollow	491	—	Hollow	684	—
	HollowE	739	701	BridgeS	694	600	BridgeL	—	—
	Bridge	—	—	BridgeL	731	—	BridgeS	623	584
**Ru**	Top	502	571	Top	555	—	Top	555	643
	Hollow	718	—	Hollow	538	—	Hollow	729	—
	HollowE	654	607	BridgeS	664	602	BridgeL	732	—
	Bridge	—	—	BridgeL	716	—	BridgeS	666	689

**Os**	Top	490	502	Top	576	576	Top	582	615
	Hollow	721	—	Hollow	504	—	Hollow	716	—
	HollowE	655	508	BridgeS	679	513	BridgeL	—	—
	Bridge	—	—	BridgeL	699	—	BridgeS	698	608
**Co**	Top	—	572	Top	465	550	Top	529	608
	Hollow	660	581	Hollow	523	—	Hollow	691	—
	HollowE	638	652	BridgeS	583	589	BridgeL	—	—
	Bridge	—	—	BridgeL	666	—	BridgeS	567	661
**Zn**	Top	—	421	Top	—	382	Top	—	491
	Hollow	—	438	Hollow	225	337	Hollow	509	—
	HollowE	397	461	BridgeS	—	421	BridgeL	—	—
	Bridge	—	—	BridgeL	—	413	BridgeS	—	531
**Cd**	Top	—	367	Top	183	345	Top	—	404
	Hollow	—	383	Hollow	157	290	Hollow	411	—
	HollowE	344	397	BridgeS	—	384	BridgeL	—	—
	Bridge	—	—	BridgeL	—	377	BridgeS	—	441

Table S4. C height, *h*, with respect to the TM surface plane, h^{sur} and h_{sub}, respectively, for all different positions studied for (001), (011), and (111) surfaces of *fcc* TMs. All values are given in pm.

TM	(001) Position	h^{sur}	h_{sub}	(011) Position	h^{sur}	h_{sub}	(111) Position	h^{sur}	h_{sub}
Rh	Top	170	-194	Top	169	-178	Top	169	-182
	Hollow	16	---	Hollow	47	—	Hollow *hcp*	96	-76
	Bridge	107	-95	BridgeS	115	-126	Hollow *fcc*	94	-108
	—	—	—	BridgeL	5	—	Bridge	—	—
Ir	Top	174	-189	Top	173	-176	Top	173	-184
	Hollow	57	—	Hollow	62	—	Hollow *hcp*	99	-76
	Bridge	108	-94	BridgeS	121	-120	Hollow *fcc*	97	-105
	—	—	—	BridgeL	25	—	Bridge	—	—
Ni	Top	164	-182	Top	162	-171	Top	164	-172
	Hollow	21	—	Hollow	37	—	Hollow *hcp*	95	-68
	Bridge	105	-71	BridgeS	110	-118	Hollow *fcc*	99	-102
	—	—	—	BridgeL	10	—	Bridge	—	—
Pd	Top	173	-202	Top	172	-187	Top	173	-187
	Hollow	—	-1	Hollow	19	—	Hollow *hcp*	88	-70
	Bridge	98	-98	BridgeS	107	-135	Hollow *fcc*	80	-116
	—	—	—	BridgeL	—	-17	Bridge	—	—
Pt	Top	173	-190	Top	173	-185	Top	174	-189
	Hollow	6	—	Hollow	31	—	Hollow *hcp*	88	-75
	Bridge	107	-95	BridgeS	113	-69	Hollow *fcc*	76	-112
	—	—	—	BridgeL	—	-8	Bridge	—	—
Cu	Top	175	-192	Top	173	-181	Top	176	-182
	Hollow	33	—	Hollow	38	—	Hollow *hcp*	98	-72
	Bridge	101	-66	BridgeS	—	-111	Hollow *fcc*	100	-98
	—	—	—	BridgeL	17	—	Bridge	—	—
Ag	Top	194	-220	Top	192	-204	Top	195	-207
	Hollow	38	—	Hollow	37	—	Hollow *hcp*	104	-72

	Bridge	157	-86	BridgeS	—	-125	Hollow *fcc*	105	-109
	—	—	—	BridgeL	10	—	Bridge	—	—
**Au**	Top	186	-202	Top	185	-201	Top	187	-204
	Hollow	51	—	Hollow	15	—	Hollow *hcp*	79	-58
	Bridge	102	-85	BridgeS	72	-51	Hollow *fcc*	84	-87
	—	—	—	BridgeL	—	-9	Bridge	—	—

Table S5. C height, h, with respect to the TM surface plane, h^{sur} and h_{sub}, respectively, for all different positions studied for (001), (011), and (111) surfaces of *bcc* TMs. All values are given in pm.

TM	(001) Position	h^{sur}	h_{sub}	(011) Position	h^{sur}	h_{sub}	(111) Position	h^{sur}	h_{sub}
**V**	Top	175	—	Top	175	-44	Top	175	-175
	Hollow	56	—	Hollow	38	—	Hollow *hcp*	79	—
	Bridge	1	-119	HollowT	—	-81	Hollow *fcc*	17	—
	—	—	—	Bridge	1	—	Bridge	26	—
**Nb**	Top	185	—	Top	187	-50	Top	186	-172
	Hollow	52	—	Hollow	25	—	Hollow *hcp*	191	—
	Bridge	121	-17	HollowT	—	-110	Hollow *fcc*	20	—
	—	—	—	Bridge	120	—	Bridge	28	—
**Ta**	Top	188	—	Top	190	-53	Top	190	-186
	Hollow	52	—	Hollow	83	—	Hollow *hcp*	192	—
	Bridge	116	-10	HollowT	—	-119	Hollow *fcc*	204	—
	—	—	—	Bridge	125	—	Bridge	34	—
**Cr**	Top	168	—	Top	171	-182	Top	167	-170
	Hollow	61	—	Hollow	107	—	Hollow *hcp*	78	—
	Bridge	125	-1	HollowT	—	-90	Hollow *fcc*	35	—
	—	—	—	Bridge	118	—	Bridge	41	—
**Mo**	Top	177	—	Top	181	-44	Top	176	-179
	Hollow	57	—	Hollow	104	—	Hollow *hcp*	84	—
	Bridge	127	-2	HollowT	—	-96	Hollow *fcc*	43	—
	—	—	—	Bridge	125	—	Bridge	38	—
**W**	Top	183	—	Top	188	-45	Top	182	-181
	Hollow	59	—	Hollow	108	—	Hollow *hcp*	191	—
	Bridge	132	-4	HollowT	—	-98	Hollow *fcc*	52	—
	—	—	—	Bridge	130	—	Bridge	46	—
**Fe**	Top	160	—	Top	164	-91	Top	159	-182
	Hollow	40	—	Hollow	89	-96	Hollow *hcp*	186	—
	Bridge	9	-118	HollowT	—	-104	Hollow *fcc*	12	—
	—	—	—	Bridge	117	—	Bridge	21	—

Table S6. C height (h) with respect to the TM surface plane, h^{sur} and h_{sub}, respectively, for all different positions studied for (0001), $(10\bar{1}0)$, and $(11\bar{2}0)$ surfaces of *hcp* TMs. All values are given in pm.

TM	(0001) Position	h^{sur}	h_{sub}	$(10\bar{1}0)$ Position	h^{sur}	h_{sub}	$(11\bar{2}0)$ Position	h^{sur}	h_{sub}
**Sc**	Top	—	-202	Top	—	-203	Top	—	-157
	Hollow	2	—	Hollow	33	—	Hollow	19	—
	HollowE	—	-123	BridgeS	—	-89	BridgeL	—	—
	Bridge	—	---	BridgeL	10	—	BridgeS	—	-139
**Y**	Top	—	-236	Top	—	-218	Top	—	-185
	Hollow	2	—	Hollow	26	—	Hollow	1	—
	HollowE	—	-132	BridgeS	—	-100	BridgeL	—	—
	Bridge	—	—	BridgeL	1	—	BridgeS	—	-151
**Ti**	Top	—	-57	Top	—	-187	Top	—	-98
	Hollow	43	—	Hollow	57	—	Hollow	38	—
	HollowE	98	-120	BridgeS	—	-47	BridgeL	—	—
	Bridge	—	—	BridgeL	32	—	BridgeS	—	-124
**Zr**	Top	—	-79	Top	197	—	Top	—	-108
	Hollow	17	—	Hollow	51	—	Hollow	31	—
	HollowE	96	-131	BridgeS	—	-59	BridgeL	—	—
	Bridge	—	—	BridgeL	23	—	BridgeS	—	-140
**Hf**	Top	—	-60	Top	199	-198	Top	—	-149
	Hollow	27	—	Hollow	202	—	Hollow	35	—
	HollowE	95	-124	BridgeS	—	-59	BridgeL	—	—
	Bridge	—	—	BridgeL	29	—	BridgeS	—	-137
**Tc**	Top	176	—	Top	172	-96	Top	—	-140
	Hollow	108	-86	Hollow	176	—	Hollow	59	—
	HollowE	111	-97	BridgeS	128	-74	BridgeL	—	—
	Bridge	—	—	BridgeL	52	—	BridgeS	126	-96
**Re**	Top	180	-79	Top	179	—	Top	—	-143
	Hollow	111	—	Hollow	186	—	Hollow	71	—
	HollowE	113	-100	BridgeS	124	-60	BridgeL	—	—
	Bridge	—	—	BridgeL	52	—	BridgeS	132	-100
**Ru**	Top	172	-182	Top	171	—	Top	169	-136
	Hollow	101	—	Hollow	182	—	Hollow	52	—
	HollowE	100	-95	BridgeS	111	-85	BridgeL	33	—
	Bridge	—	—	BridgeL	96	—	BridgeS	125	-95

**Os**	Top	176	-184	Top	174	-180	Top	172	-128
	Hollow	104	—	Hollow	185	—	Hollow	46	—
	HollowE	107	-94	BridgeS	121	-88	BridgeL	—	—
	Bridge	—	—	BridgeL	101	—	BridgeS	132	-87
**Co**	Top	—	-171	Top	160	-170	Top	77	-131
	Hollow	97	-85	Hollow	171	—	Hollow	45	—
	HollowE	101	-96	BridgeS	98	-84	BridgeL	—	—
	Bridge	—	—	BridgeL	48	—	BridgeS	112	-95
**Zn**	Top	—	-154	Top	—	-201	Top	—	-99
	Hollow	—	-25	Hollow	190	-198	Hollow	1	—
	HollowE	86	-130	BridgeS	—	-52	BridgeL	—	—
	Bridge	—	—	BridgeL	—	-47	BridgeS	—	-45
**Cd**	Top	—	-225	Top	217	-229	Top	—	-156
	Hollow	—	-42	Hollow	214	-225	Hollow	-41	—
	HollowE	95	-151	BridgeS	—	-33	BridgeL	—	—
	Bridge	—	—	BridgeL	—	-57	BridgeS	—	-51

Section S4: K-means Inertia and Silhouette Scores

Figure S4. Evolution of inertia as a function of the number of employed clusters in the $E_{ads/abs}$ *vs.* h quadrant plots. Within the elbow method, the optimal number of clusters would be three, belonging to the inflection point in the overall trend.

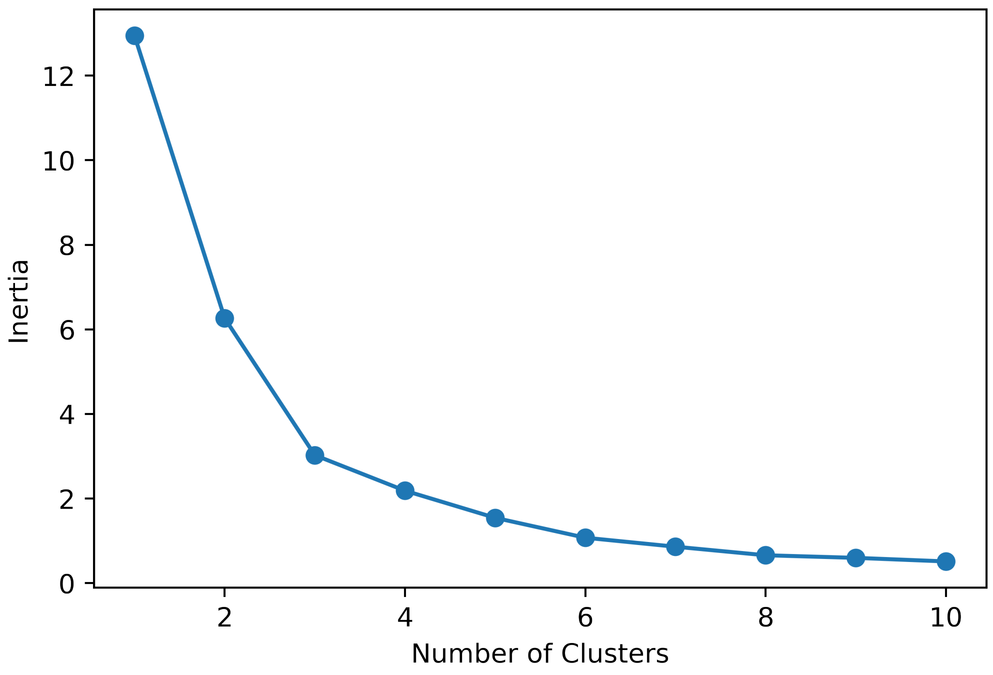

Figure S5. Silhouette score as a function the number of employed clusters.

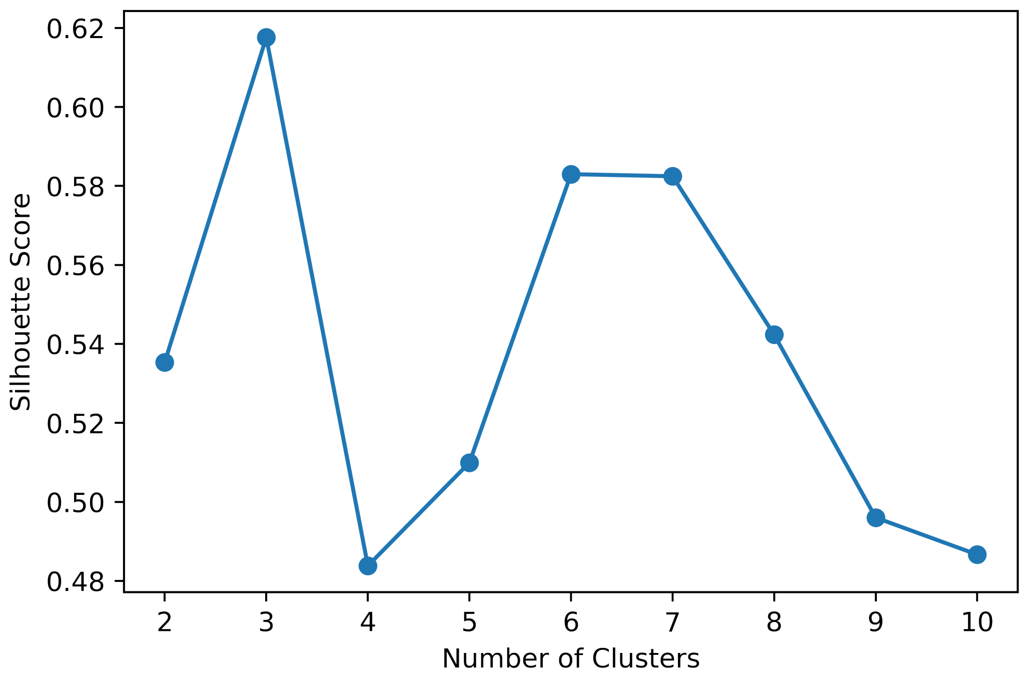

Section S5: Phase Diagrams

Figure S6. Calculated phase diagrams for *fcc* TMs (001), (011), and (111) surfaces depending on the acetylene partial pressure, $p_{C_2H_2}$, and temperature, *T*. Diagrams are obtained for a constant partial pressure of H_2, $p_{H_2} = 10^{-7}$ Pa, and distinguish surface, C^{sur}, and subsurface, C_{sub}, situations. Regions above or below each curve indicate conditions at which the C-containing or pristine surfaces, respectively, are thermodynamically preferred.

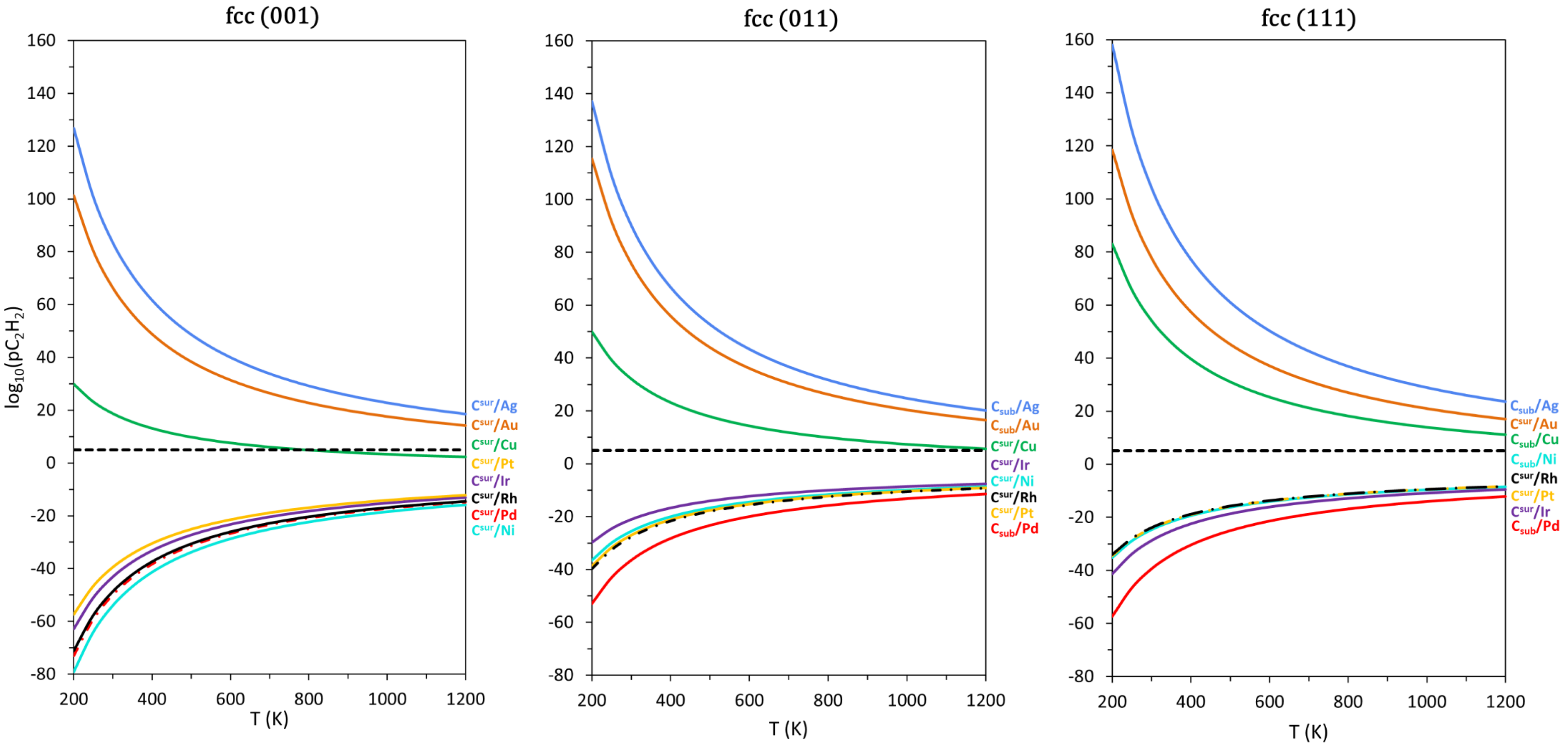

Figure S7. Calculated phase diagrams for *hcp* TMs for (0001), $(10\bar{1}0)$, and $(11\bar{2}0)$ surfaces depending on the acetylene partial pressure, $p_{C_2H_2}$, and temperature, *T*. Diagrams are obtained for a constant partial pressure of H_2, $p_{H_2} = 10^{-7}$ Pa, and distinguish surface, C^{sur}, and subsurface, C_{sub}, situations. Regions above or below each curve indicate conditions at which the C-containing or pristine surfaces, respectively, are thermodynamically preferred.

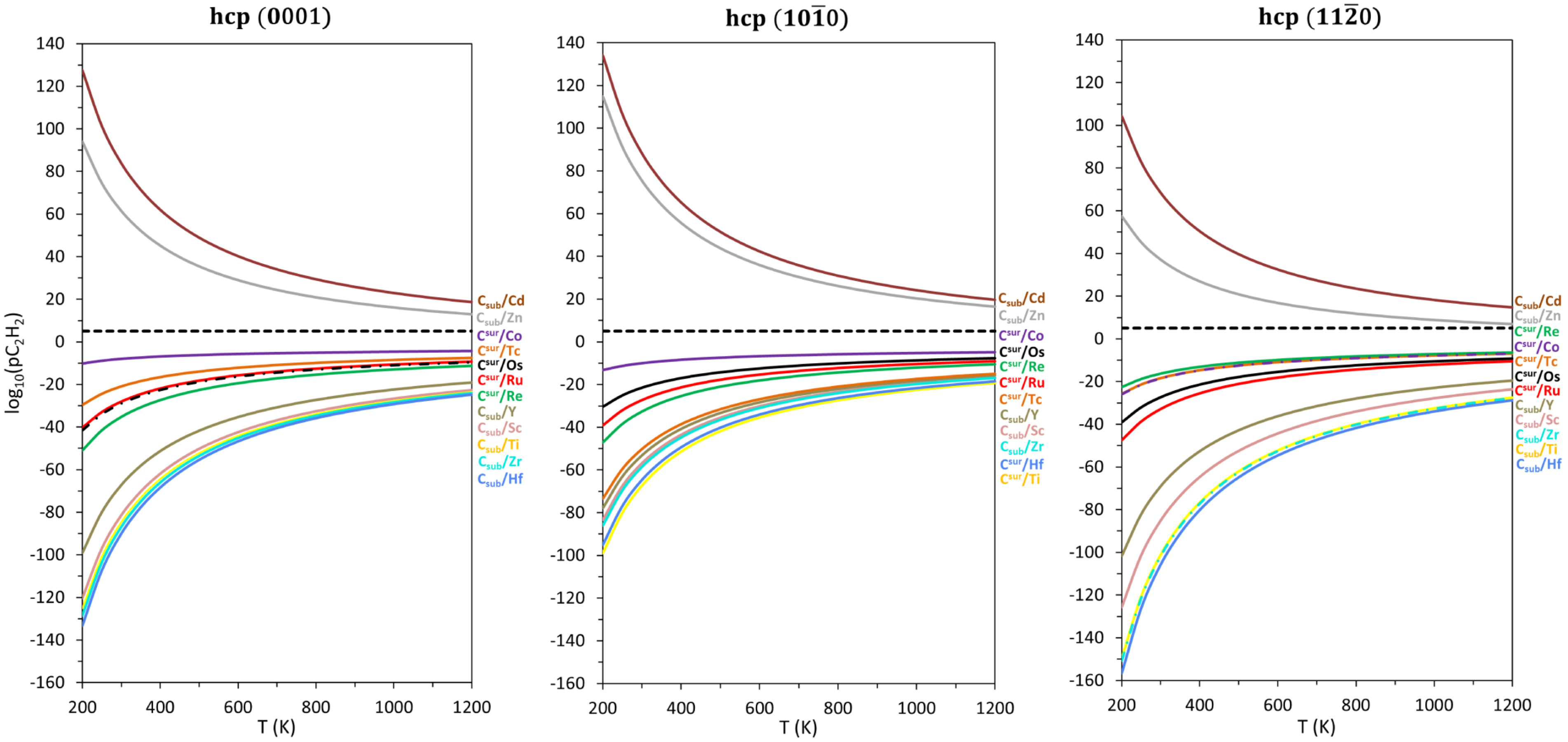

Figure S8. Calculated phase diagrams for *bcc* TMs (001), (011), and (111) surfaces depending on the acetylene partial pressure, $p_{C_2H_2}$, and temperature, *T*. Diagrams are obtained for a constant partial pressure of H_2, $p_{H_2} = 10^{-7}$ Pa, and distinguish surface, C^{sur}, and subsurface, C_{sub}, situations. Regions above or below each curve indicate conditions at which the C-containing or pristine surfaces, respectively, are thermodynamically preferred.

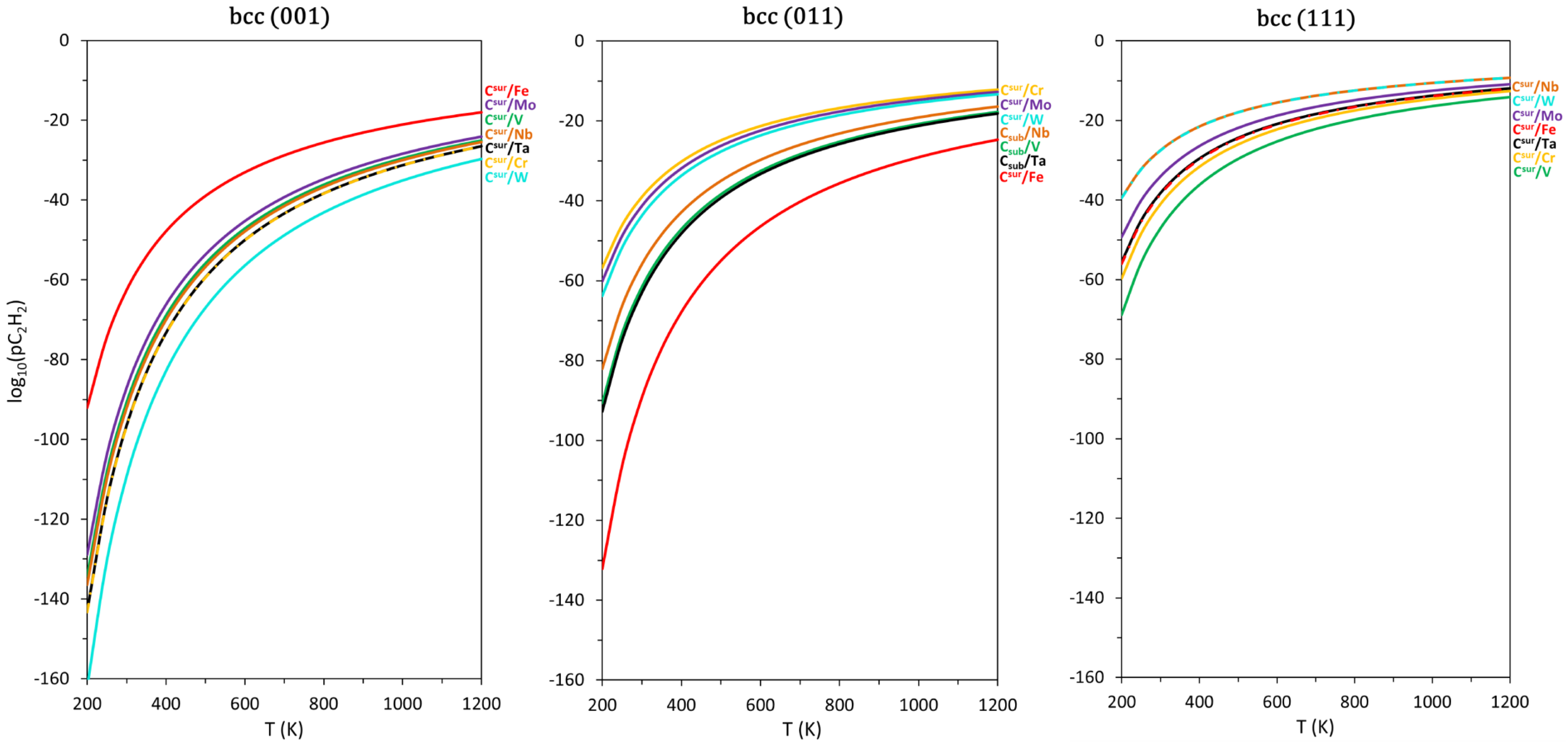

Section S6: Isolated Descriptors Evaluation

Figure S9. Linear correlation between the *d*-band centre, ε_d, and most stable positions $E_{ads/abs}$. The regression coefficient, R, is displayed.

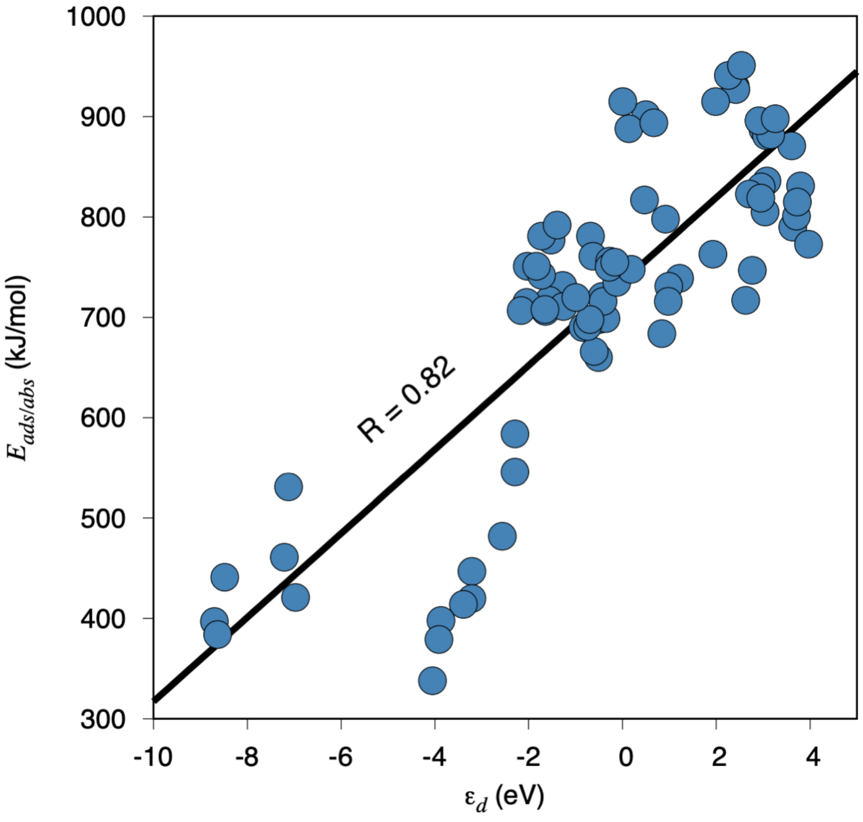

Figure S10. Linear correlation between the width-corrected *d*-band centre, ε_d^W, and most stable positions $E_{ads/abs}$. The regression coefficient, *R*, is displayed.

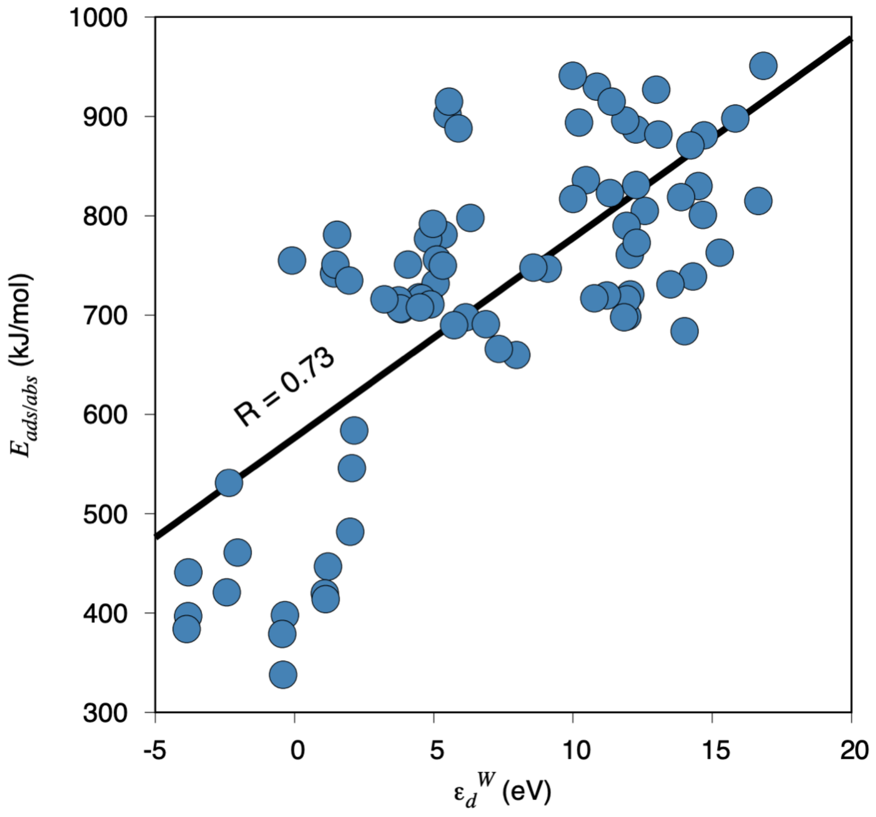

Figure S11. Linear correlation between the maximum Hilbert peak, ε_u, and most stable positions $E_{ads/abs}$. The regression coefficient, R, is displayed.

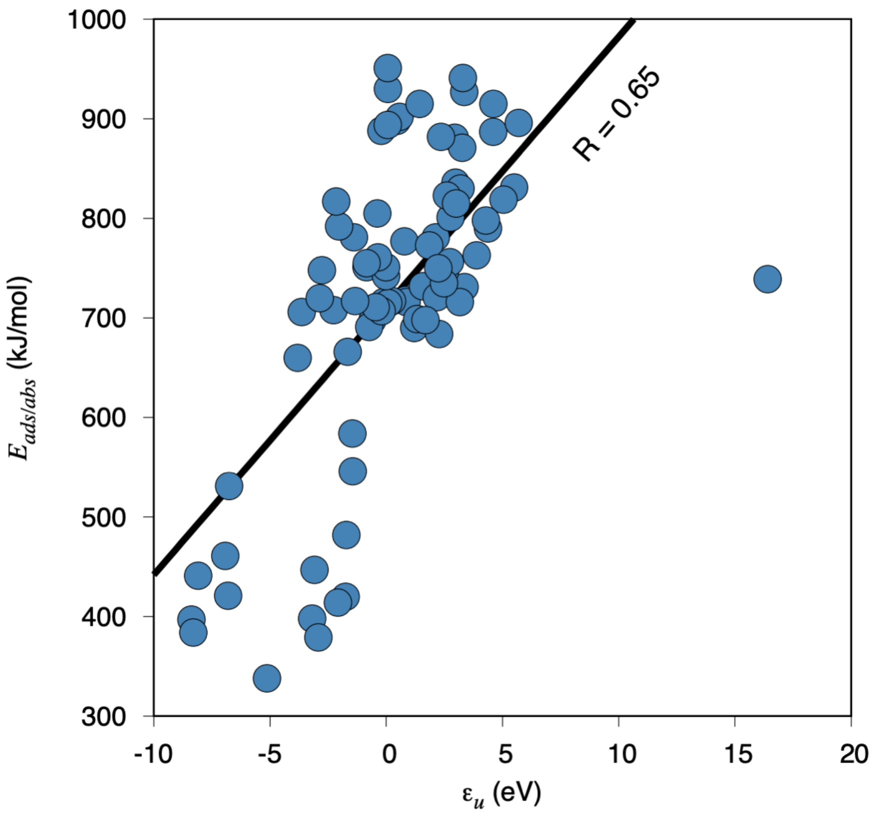

Figure S12. Linear correlation between the surface energy, γ, and most stable positions $E_{ads/abs}$. The regression coefficient, *R*, is displayed.

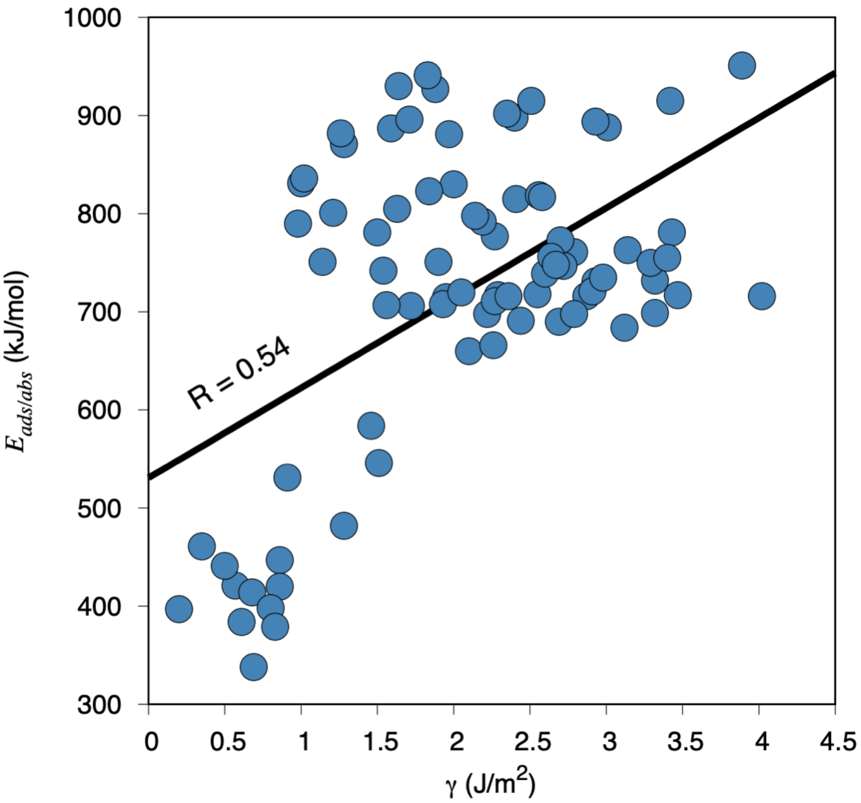

Figure S13. Correlation between the surface work function, ϕ, and most stable positions $E_{ads/abs}$. The regression coefficient, R, is displayed.

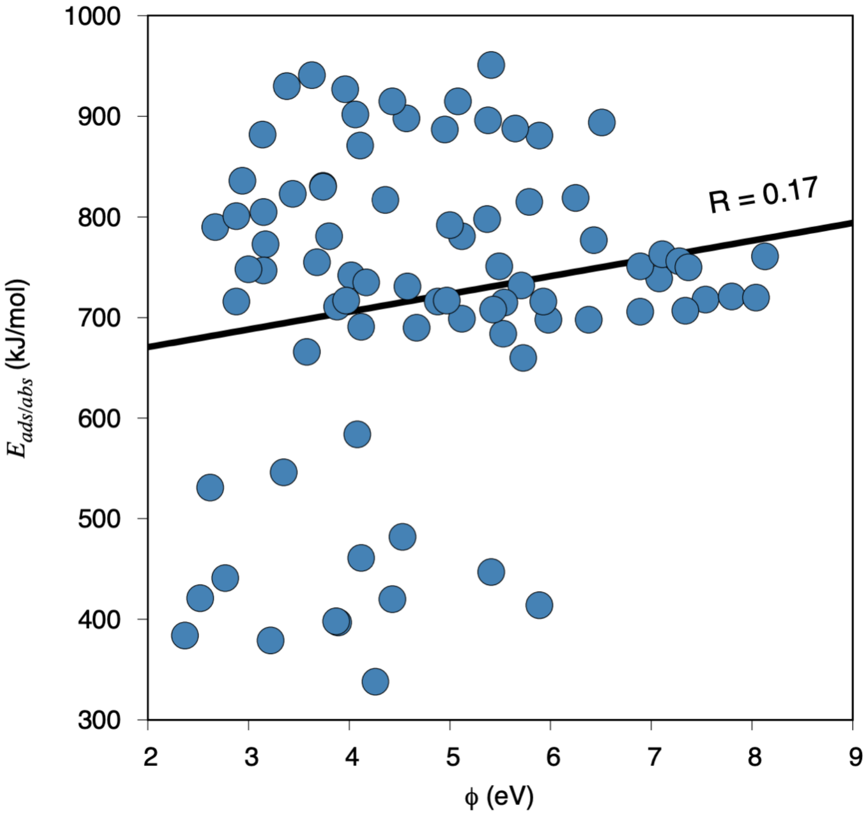

Figure S14. Correlation between ε_d and most stable positions $E_{ads/abs}$. A regression line is shown for each of the nine different studied surfaces. Correlation coefficients of 0.91, 0.89, 0.93, 0.98, 0.93, 0.96, 0.41, 0.28, and 0.23 are found for *fcc* (001), *fcc* (011), *fcc* (111), *hcp* (0001), *hcp* (10$\bar{1}$0), *hcp* (11$\bar{2}$0), *bcc* (001), *bcc* (011), and *bcc* (111), respectively.

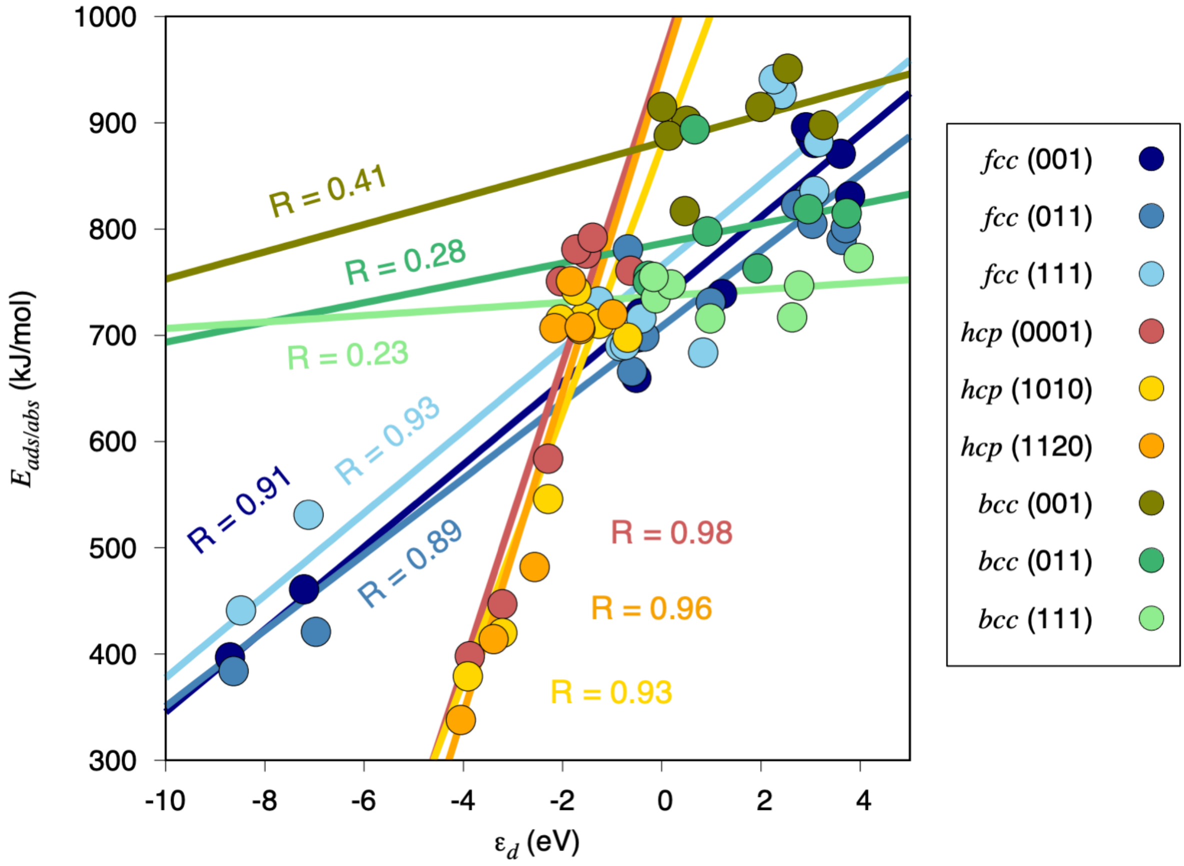

Figure S15. Most stable $E_{ads/abs}$ situations with respect to ε_d, and the corresponding linear correlations, disregarding Groups III and IV TMs for *hcp* cases. Values in blue correspond to *fcc* TMs, green to *bcc* TMs, and purple to *hcp* TMs.

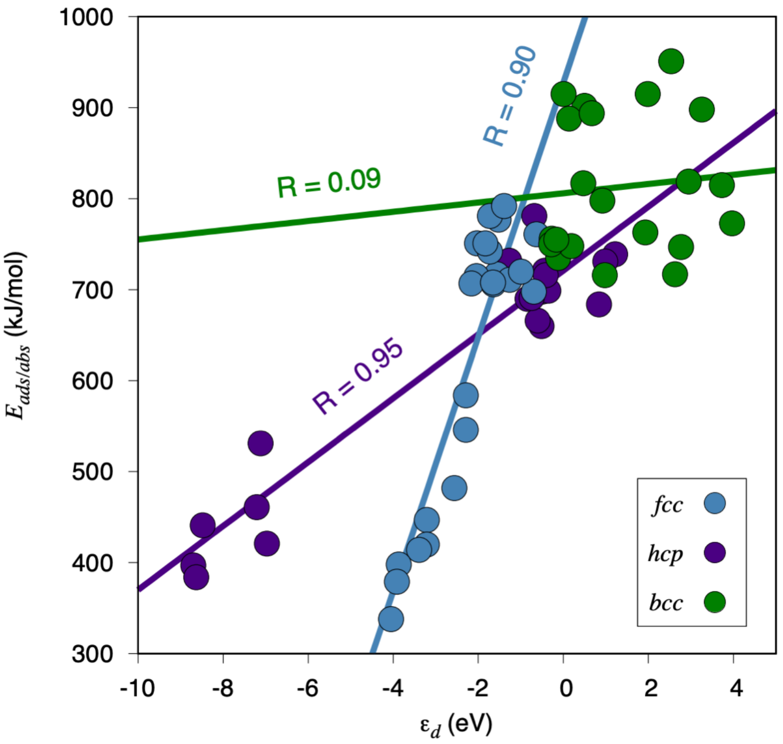

Section S7: Machine Learning Regression Algorithms

All the machine learning (ML) algorithms used in this work were applied as implemented in the *sklearn* Python library.[1] The multivariable linear regression (MLR) simply corresponds to the ordinary least squares linear regression but as a function of two or more variables. The decision tree regressor (DTR) is a supervised learning algorithm that has, as a goal, to build a model predicting the value of a target variable, in our case the E_{ads} or E_{abs}, through the learning of a set of binary rules derived from the data features, here the descriptors and surface features listed in the manuscript. For this DTR builds a model in the form of a tree structure featuring branches, nodes, and leaves. The order of the questions —known as decision nodes, which gives the name to the method— as well as their content is automatically determined and optimized by the algorithm, which looks for the homogeneity of y values in a found subset, and for that, uses the standard deviation of y values as an optimization criterion. During the training, the model learns any relationships between the data and the target variable, defining the best questions as well as their order to make the most accurate estimates possible. When predicting the dependent variable value of a new data point, this point is run completely through the entire tree branches by answering the node questions —posed as logic steps— until reaching the final answer leaf, with the target variable. Indeed, this variable value is just the average value of all points satisfying the same logic questions as the dependent point.

The complexity of the above explanation calls for an illustrative example so as to understand the basics of the procedure. Let us define a dependent variable target, y —which could be, *e.g.*, E_{ads}—, which depends on two features, x_1 and x_2 —*e.g.*, two descriptors, such as ε_d and γ. Figure S14 displays a scatter plot of x_2 *vs.* x_1, and one could well imagine a three-dimensional plot, where y values would define the points heights in an orthogonal axis to x_1 and x_2. In it, dotted lines would delimit regions defined by the nodes, *e.g.*, a first one asking whether x_1 values is larger than 50, and second ones asking whether x_2 value is larger than 4 for x_1 values smaller than 50, or x_2 value being larger than 12 when x_1 is larger than 50. That leads to final four leaves, with different average y values, $\bar{y}$, see Figure S15. For instance, when this is done for a training set, a test set value with x_1 = 25 and x_2 = 12 would yield an expected $\bar{y}$ value of 5.1. Notice that this is done for a very simplified, two-dimensional case, with y values depending on two variables, but is easily applied for the dependence on a higher number of variables.

Notice that, even if DTR is relatively easy to interpret, and requires little data preparation, the resulting tree is sensitive to the employed data, *i.e.* small variations on the data may be translated into a completely different built tree. To overcome this drawback, an ensemble of trees, *a.k.a.* a forest, can be used. This is indeed the basics of the random forest regression (RFR), where an ensemble of decision trees is grown. Each tree is assembled from a sample randomly drawn from the training set. For each

tree, when splitting each node, the best split can be found either from all the input features or for a random subset of features, *a.k.a. max_features*. These two sources of randomness help decreasing the variance of the estimator, since individual decision trees usually exhibit high variance and tend to overfit. The background idea is that, when a prediction is cast on a data set, *e.g.* the above commented x_1 and x_2 values, different $\bar{y}_i$ values are obtained for $i = 1 - N$, where N is the number of trees of the forest, a.k.a. *n_estimators*. Thus, the expected value of $\bar{y}$ is simply the average over the expected $\bar{y}_i$ values on the different N trees. By doing so, the predictive accuracy of the trees is narrowed, diluting possible extreme $\bar{y}$ forecasts. Notice that RFR becomes DTR for $i = 1$, and that the more the trees, the better the accuracy is, yet computationally more expensive. Aside, notice that accuracy decays with N, but results do not normally improve beyond a critical number of trees.

An appealing aspect of both DTR and RFR is the fact that the relative rank of a feature used as a decision node in a tree can be used to assess the relative importance of that feature with respect to the target variable predictions. For instance, features used at the top of the tree have a larger impact, since they affect the final prediction decision of a larger fraction of samples. Hence, the relative importance of the features can be estimated as the expected fraction of samples they contribute to.

Figure S16. Exemplary scatter plot of values, shown as blue circles, of x_2 *vs.* x_1 variables, where black dotted lines represent the variable splitting decisions learned by the model. In light blue, the $\bar{y}$ average values for all those points belonging in each of the resulting sections inside the plot.

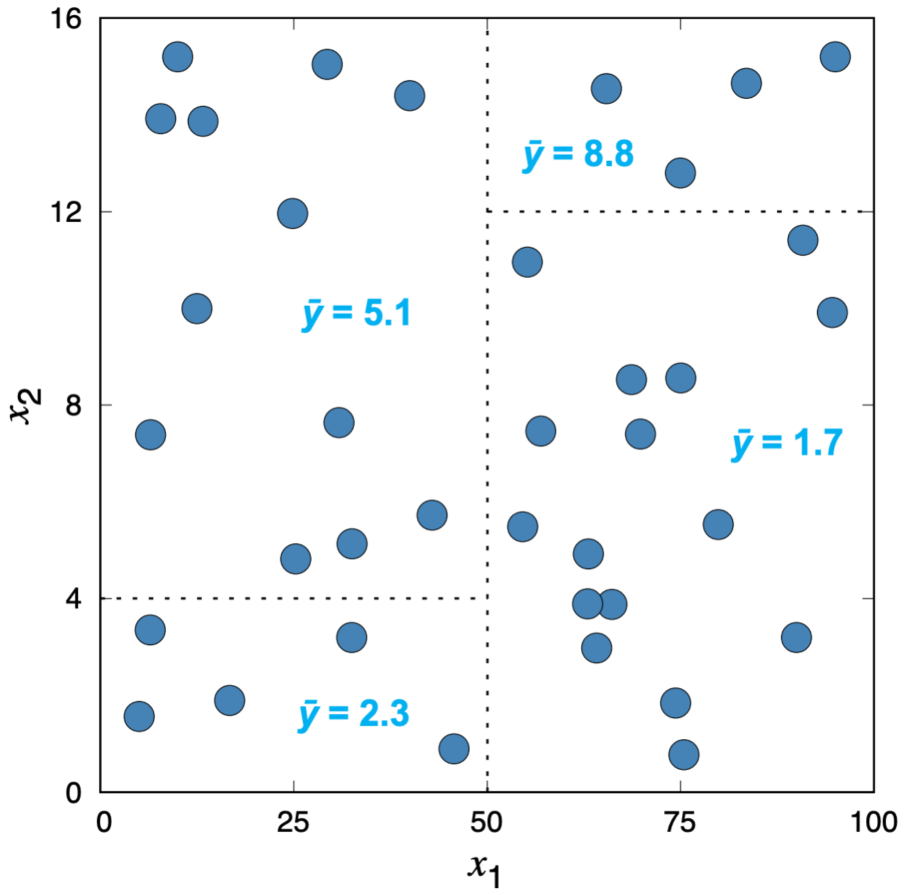

Figure S17. Exemplary decision tree from data shown in Figure S14.

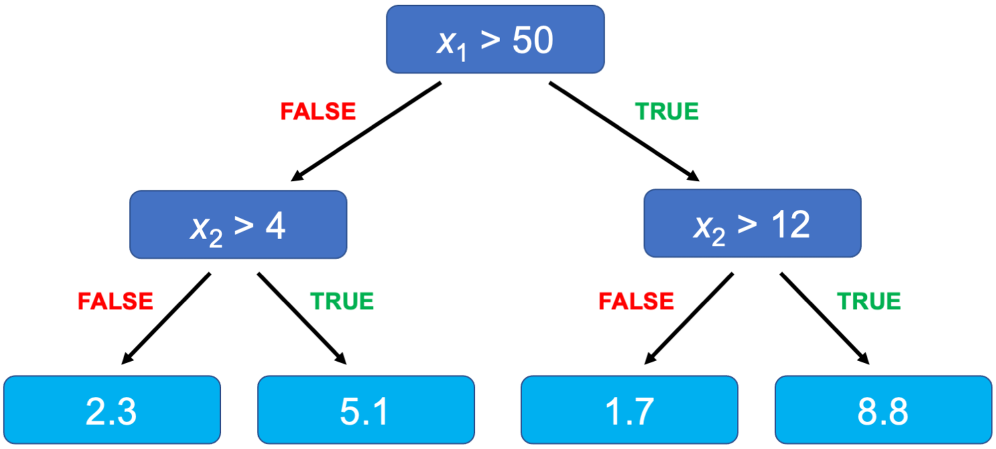

Section S8: Diffusion Energy Barriers

In order to evaluate the different diffusion E_b energy barriers, different paths have been explored for all the nine different TM surface types. Notice that, for some surfaces, and some sorts of diffusions, different paths have been evaluated, normally implying different adsorption or absorption minima. As an example of many, *fcc* (111) surfaces normally display two possible adsorption minima, Hollow *fcc* or Hollow *hcp*, see Figure S2. C atoms can sink from these two adsorption minima towards two different absorption minima, these are, subsurface Hollow *fcc* or subsurface Hollow *hcp*. Since normally such surface minima are close in energy, we decided to evaluate both possible subsurface sinking paths to have a more complete description, although the final barrier used for a further evaluation was the lowest explored for a same process on the same surface.

Table S7. Explored paths for surface (E_{sur}), subsurface (E_{sub}), sinking (E_{sink}), and emerging (E_{emer}) diffusions on *fcc* TMs and their labelling. For each path, different minima stages are defined, with a subscript S to denote subsurface positions.

Surface	Barrier	Path 1	Path 2	Label Path 1	Label Path 2
**(001)**	E_{sur}	Hollow/Bridge/Hollow	—	a1	—
	E_{sub}	Top_S/$Bridge_S$/Top_S	$Bridge_S$/Top_S/$Bridge_S$	a2	a3
	E_{sink}	Hollow/Top_S	—	a4	—
	E_{emer}	Top_S/Hollow	—	a5	—
**(011)**	E_{sur}	BridgeS/Hollow/BridgeS	—	a6	—
	E_{sub}	$BridgeL_S$/Top_S/$BridgeL_S$	—	a7	—
	E_{sink}	BridgeS/$BridgeL_S$	Hollow/$BridgeL_S$	a8	a9
	E_{emer}	$BridgeL_S$/BridgeS	$BridgeL_S$/Hollow	a10	a11
**(111)**	E_{sur}	Hollow *fcc*/Hollow *hcp*/Hollow *fcc*	Hollow *hcp*/Hollow *fcc*/Hollow *hcp*	a12	a13
	E_{sub}	Hollow fcc_S/Hollow hcp_S/Hollow fcc_S	Hollow hcp_S/Hollow fcc_S/Hollow hcp_S	a14	a15
	E_{sink}	Hollow *fcc*/Hollow fcc_S	Hollow *hcp*/Hollow hcp_S	a16	a17
	E_{emer}	Hollow fcc_S/Hollow *fcc*	Hollow hcp_S/Hollow *hcp*	a18	a19

Table S8. Explored paths for surface (E_{sur}), subsurface (E_{sub}), sinking (E_{sink}), and emerging (E_{emer}) diffusions on *bcc* TMs and their labelling. For each path, different minima stages are defined, with a subscript S to denote subsurface positions.

Surface	Barrier	Path 1	Path 2	Label Path 1	Label Path 2
**(001)**	E_{sur}	Hollow/Bridge/Hollow	—	b1	—
	E_{sub}	Top_S/$Bridge_S$/Top_S	$Bridge_S$/$Hollow_S$/$Bridge_S$	b2	b3
	E_{sink}	Hollow/Top_S	—	b4	—
	E_{emer}	Top_S/Hollow	—	b5	—
**(011)**	E_{sur}	Hollow/Bridge/Hollow	—	b6	—
	E_{sub}	$HollowT_S$/Hollow/$HollowT_S$	—	b7	—
	E_{sink}	Hollow/$HollowT_S$	—	b8	—
	E_{emer}	$HollowT_S$/Hollow	—	b9	—
**(111)**	E_{sur}	Bridge/Hollow *fcc*/Bridge	Hollow *fcc*/Hollow *hcp*/Hollow *fcc*	b10	b11
	E_{sub}	Top_S/$Bridge_S$/Top_S	—	b12	—
	E_{sink}	Bridge/Top_S	Hollow *fcc*/Top_S	b13	b14
	E_{emer}	Top_S/Bridge	Top_S/Hollow *fcc*	b15	b16

Table S9. Explored paths for surface (E_{sur}), subsurface (E_{sub}), sinking (E_{sink}), and emerging (E_{emer}) diffusions on *hcp* TMs and their labelling. For each path, different minima stages are defined, with a subscript S to denote subsurface positions.

Surface	Barrier	Path 1	Path 2 ; 3 ; 4	Label Path 1	LabelS PathS 2 ; 3 ; 4
**(0001)**	E_{sur}	Hollow/HollowE/Hollow	HollowE/Hollow/HollowE	c1	c2
	E_{sub}	HollowE$_S$/Hollow$_S$/HollowE$_S$	—	c3	—
	E_{sink}	Hollow/HollowE$_S$	HollowE/HollowE$_S$	c4	c5
	E_{emer}	HollowE$_S$/Hollow	HollowE$_S$/HollowE	c6	c7
**(10$\bar{1}$0)**	E_{sur}	BridgeL/BridgeS/BridgeL	BridgeS/BridgeL/BridgeS	c8	c9
	E_{sub}	BridgeS$_S$/BridgeL$_S$/BridgeS$_S$	Top$_S$/BridgeL$_S$/Top$_S$	c10	c11
	E_{sink}	BridgeL/BridgeS$_S$	BridgeL/Top$_S$; BridgeS/Top$_S$	c12	c13 ; c13.2
	E_{emer}	BridgeS$_S$/BridgeL	Top$_S$/BridgeL ; Top$_S$/BridgeS	c14	c15 ; c15.2
**(11$\bar{2}$0)**	E_{sur}	Hollow/BridgeS/Hollow	BridgeS/BridgeL/BridgeS ; BridgeL/BridgeS/BridgeL	c16	c17 ; c17.2
	E_{sub}	BridgeS$_S$/BridgeL$_S$/BridgeS$_S$	Top$_S$/BridgeL$_S$/Top$_S$	c18	c19
	E_{sink}	Hollow/BridgeS$_S$	BridgeS/Top$_S$; Hollow/Top$_S$; BridgeL/BridgeS$_S$	c20	c21 ; c21.2 ; c21.3
	E_{emer}	BridgeS$_S$/Hollow	Top$_S$ / Bridge$_S$; Top$_S$/Hollow ; BridgeS$_S$/BridgeL	c22	c23 ; c23.2 ; c23.3

Table S10. Calculated surface (E_{sur}), subsurface (E_{sub}), sinking (E_{sink}), and emerging (E_{emer}) diffusion energy barriers for all the TM surfaces featured in this work, along the associated diffusion paths defined in Tables S7-S9. All values are given in kJ mol^{-1}.

TM	Surface	E_{sur}	Path	E_{sub}	Path	E_{sink}	Path	E_{emer}	Path
	**(001)**	136.7	a1	46.5	a2	187.2	a4	64.5	a5
**Rh**	**(011)**	82.6	a6	95.7	a7	72.6	a8	13.4	a10
	**(111)**	68.6	a13	89.6	a14	90.0	a16	13.1	a18
	**(001)**	104.8	a1	28.8	a3	256.0	a4	0.7	a5
**Ir**	**(011)**	99.4	a6	116.6	a7	170.8	a8	2.8	a10
	**(111)**	74.4	a13	72.1	a14	164.2	a16	11.6	a18
	**(001)**	195.1	a1	150.6	a2	157.6	a4	49.3	a5
**Ni**	**(011)**	40.2	a6	98.7	a7	60.9	a8	57.8	a10
	**(111)**	36.2	a13	116.7	a14	64.5	a17	6.6	a19
	**(001)**	192.1	a1	80.1	a2	155.1	a4	102.4	a5
**Pd**	**(011)**	77.3	a6	112.7	a7	79.5	a8	84.7	a10
	**(111)**	71.6	a13	96.0	a14	27.3	a17	50.1	a19
	**(001)**	117.8	a1	115.5	a3	207.0	a4	64.3	a5
**Pt**	**(011)**	105.6	a6	119.6	a7	115.8	a8	61.6	a10
	**(111)**	80.2	a12	75.7	a15	39.4	a17	38.0	a19
	**(001)**	167.4	a1	13.0	a2	139.3	a4	11.0	a5
**Cu**	**(011)**	35.3	a6	79.7	a7	79.2	a9	71.6	a11
	**(111)**	8.4	a12	89.7	a14	50.7	a17	7.9	a19
	**(001)**	114.5	a1	21.2	a3	105.8	a4	17.9	a5
**Ag**	**(011)**	17.5	a6	60.9	a7	22.7	a8	37.5	a10
	**(111)**	19.5	a12	44.7	a14	32.2	a17	12.0	a19
**Au**	**(001)**	58.8	a1	96.5	a3	135.1	a4	17.4	a5

	**(011)**	34.6	a6	101.4	a7	59.9	a8	63.3	a10
	**(111)**	50.7	a12	35.8	a15	24.2	a17	7.4	a19
	**(001)**	172.4	b1	86.3	b3	208.4	b4	49.4	b5
**V**	**(011)**	67.9	b6	45.8	b7	68.0	b8	77.0	b9
	**(111)**	140.9	b11	173.5	b12	173.5	b14	1.3	b16
	**(001)**	252.2	b1	24.8	b2	233.6	b4	76.6	b5
**Nb**	**(011)**	132.8	b6	112.5	b7	82.0	b8	110.1	b9
	**(111)**	69.5	b10	166.2	b12	166.2	b13	2.4	b15
	**(001)**	263.2	b1	40.7	b2	279.5	b4	111.1	b5
**Ta**	**(011)**	126.6	b6	180.0	b7	183.3	b9	229.0	b9
	**(111)**	114.7	b10	208.1	b12	208.1	b13	1.4	b15
	**(001)**	209.5	b1	54.9	b2	232.1	b4	1.0	b5
**Cr**	**(011)**	90.9	b6	71.7	b7	109.4	b8	25.2	b9
	**(111)**	24.1	b10	210.2	b12	210.2	b13	1.5	b15
	**(001)**	192.8	b1	58.8	b2	247.8	b4	23.6	b5
**Mo**	**(011)**	86.5	b6	109.1	b7	86.4	b8	9.2	b9
	**(111)**	97.4	b10	226.3	b12	226.3	b13	1.0	b15
	**(001)**	202.1	b1	33.7	b2	326.6	b4	1.0	b5
**W**	**(011)**	92.0	b6	138.7	b7	290.9	b8	153.8	b9
	**(111)**	122.6	b10	303.7	b12	303.7	b13	1.2	b15
	**(001)**	185.5	b1	52.8	b2	182.9	b4	69.2	b5
**Fe**	**(011)**	73.4	b6	55.0	b7	131.1	b8	85.9	b9
	**(111)**	21.2	b10	82.3	b12	82.3	b13	7.6	b15
	**(0001)**	62.5	c1	72.8	c3	7.5	c4	125.4	c6
**Sc**	**($10\bar{1}0$)**	96.2	c8	49.2	c10	31.5	c12	80.4	c14
	**($11\bar{2}0$)**	64.7	c16	154.9	c18	37.5	c20	78.0	c22
**Y**	**(0001)**	67.6	c1	109.2	c3	10.2	c4	101.9	c8

	**$(10\bar{1}0)$**	34.5	c8	131.2	c10	25.3	c12	91.2	c14
	**$(11\bar{2}0)$**	13.7	c16	157.3	c18	79.5	c20	79.5	c22
	**(0001)**	20.7	c1	60.0	c3	64.7	c5	188.2	c7
**Ti**	**$(10\bar{1}0)$**	155.9	c8	107.4	c11	92.1	c13	73.6	c15
	**$(11\bar{2}0)$**	94.7	c16	214.8	c18	33.8	c20	118.6	c22
	**(0001)**	44.6	c1	78.9	c3	67.0	c5	206.6	c7
**Zr**	**$(10\bar{1}0)$**	78.0	c8	160.6	c10	89.8	c12	95.9	c14
	**$(11\bar{2}0)$**	85.8	c16	253.0	c18	67.3	c20	188.9	c22
	**(0001)**	73.6	c1	137.2	c3	39.5	c5	45.7	c8
**Hf**	**$(10\bar{1}0)$**	122.3	c8	114.9	c10	122.2	c12	114.9	c14
	**$(11\bar{2}0)$**	81.6	c16	280.5	c17	59.4	c20	193.9	c22
	**(0001)**	35.9	c1	102.0	c3	161.3	c5	146.3	c7
**Tc**	**$(10\bar{1}0)$**	60.9	c8	81.5	c11	175.3	c13	1.4	c15
	**$(11\bar{2}0)$**	80.9	c16	63.1	c18	63.7	c20	37.0	c22
	**(0001)**	41.9	c2	175.9	c3	235.5	c5	197.5	c7
**Re**	**$(10\bar{1}0)$**	46.8	c8	38.9	c10	208.8	c12	77.2	c14
	**$(11\bar{2}0)$**	53.2	c16	48.7	c19	165.9	c21.2	72.3	c23.2
	**(0001)**	72.3	c1	58.6	c3	92.6	c5	44.7	c7
**Ru**	**$(10\bar{1}0)$**	68.4	c8	11.1	c10	124.8	c12	11.1	c14
	**$(11\bar{2}0)$**	70.2	c17.2	7.7	c18	49.7	c21.3	6.9	c23.3
	**(0001)**	76.1	c1	46.5	c3	164.7	c5	17.7	c7
**Os**	**$(10\bar{1}0)$**	57.0	c8	135.2	c11	154.9	c13	31.4	c15
	**$(11\bar{2}0)$**	43.3	c16	53.8	c19	110.5	c21.2	9.1	c23.2
	**(0001)**	29.1	c1	71.8	c3	76.7	c5	91.0	c7
**Co**	**$(10\bar{1}0)$**	47.6	c8	41.0	c11	117.2	c13	0.4	c15
	**$(11\bar{2}0)$**	84.3	c16	19.3	c18	41.6	c20	12.1	c22
**Zn**	**(0001)**	37.5	c1	28.0	c3	20.0	c5	84.3	c7

	**$(10\bar{1}0)$**	40.1	c9	2.4	c11	40.0	c13.2	1.1	c15.2
	**$(11\bar{2}0)$**	149.2	c17	54.7	c19	0.8	c21	22.3	c23
	**(0001)**	22.7	c1	29.3	c3	17.9	c5	71.0	C7
**Cd**	**$(10\bar{1}0)$**	58.3	c9	36.6	c11	73.3	c13.2	34.3	c15.2
	**$(11\bar{2}0)$**	25.7	c17	71.2	c19	31.8	c21	31.8	c23

Figure S18. Top (bottom image) and side (top image) views of exemplary TSs along the different paths explored on *fcc* TMs. Brown and grey spheres denote C and metal atoms, respectively.

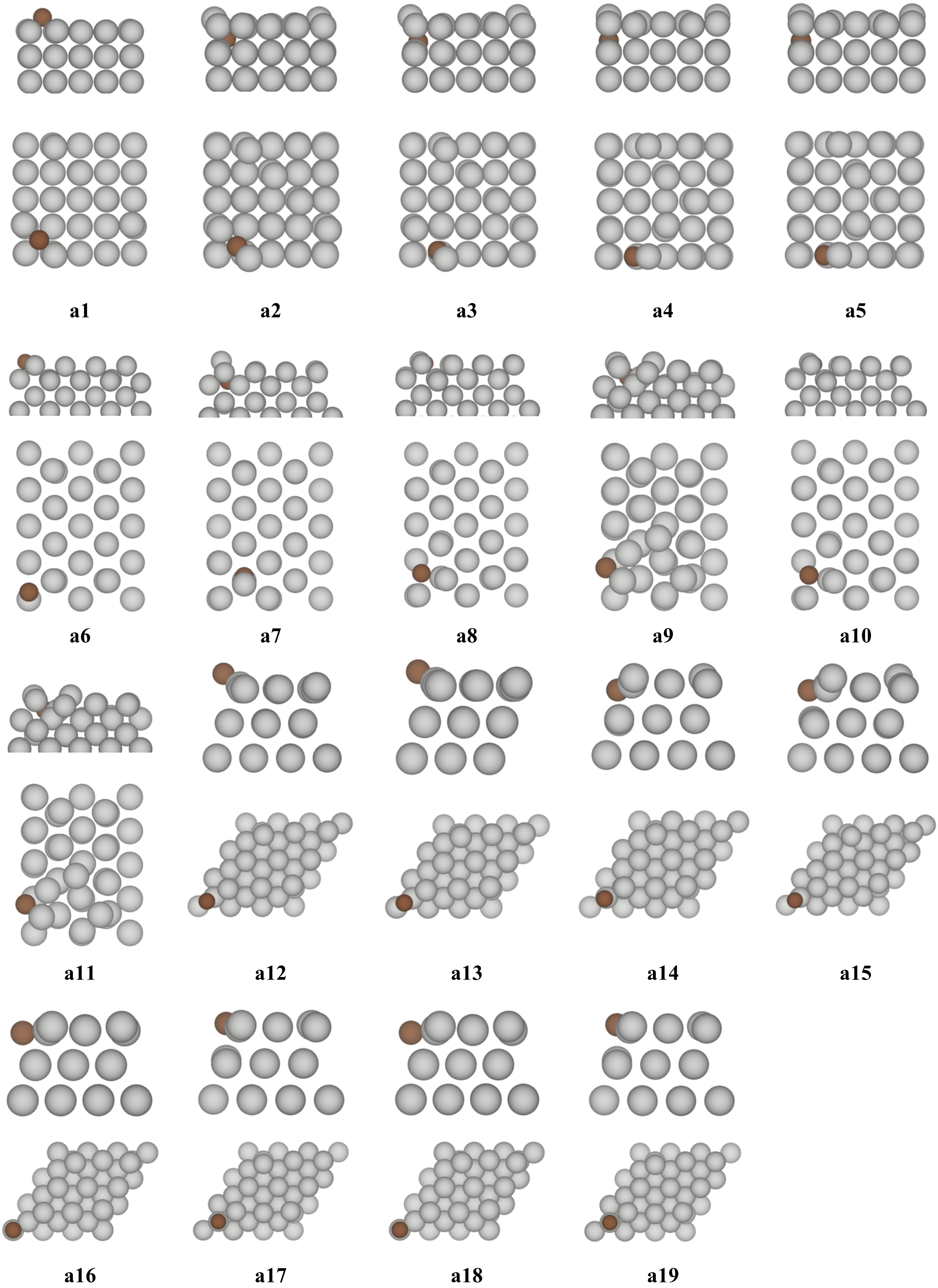

Figure S19. Top (bottom image) and side (top image) views of exemplary TSs along the different paths explored on *bcc* TMs. Brown and grey spheres denote C and metal atoms, respectively.

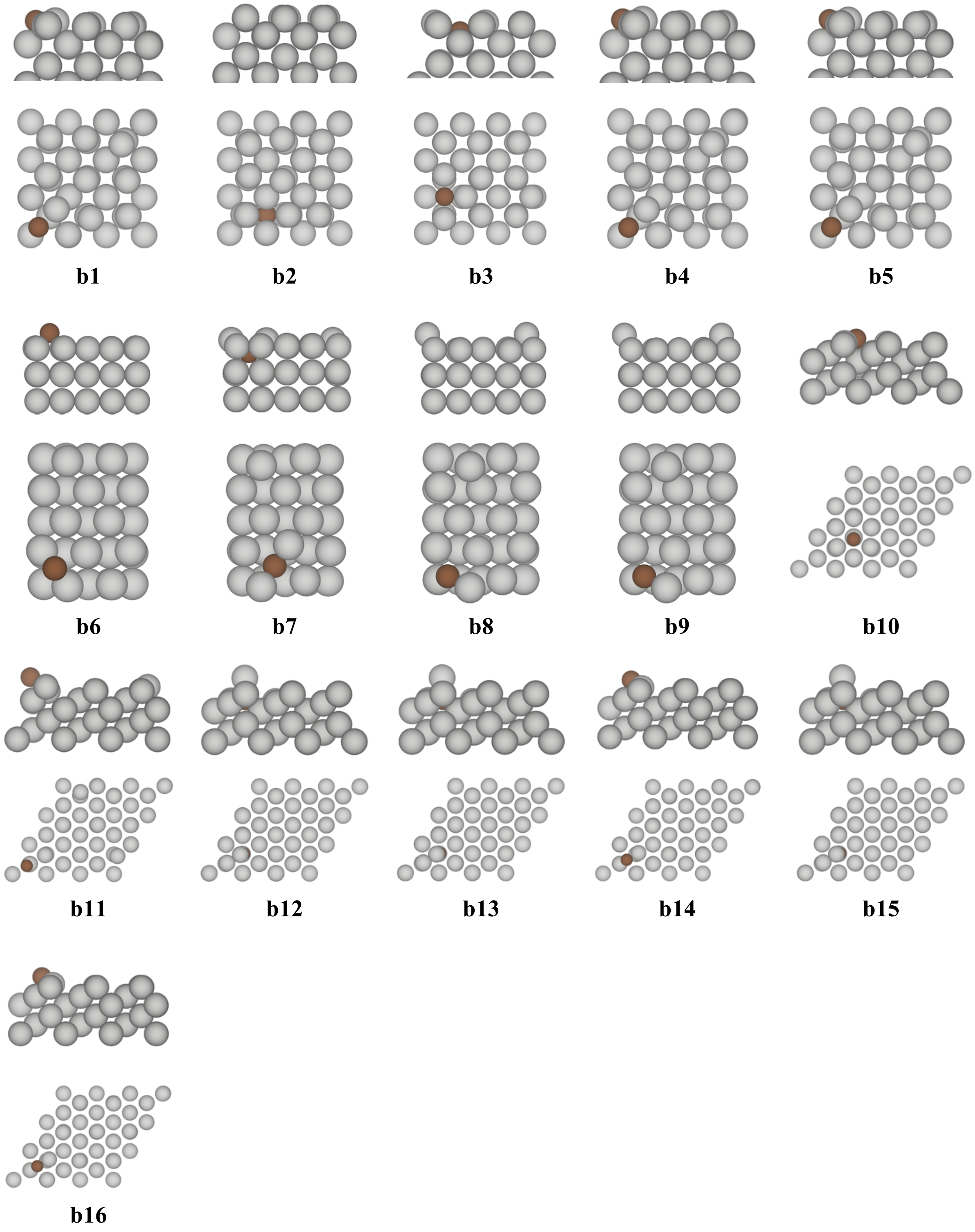

Figure S20. Top (bottom image) and side (top image) views of exemplary TSs along the different paths explored on *hcp* TMs. Brown and grey spheres denote C and metal atoms, respectively.

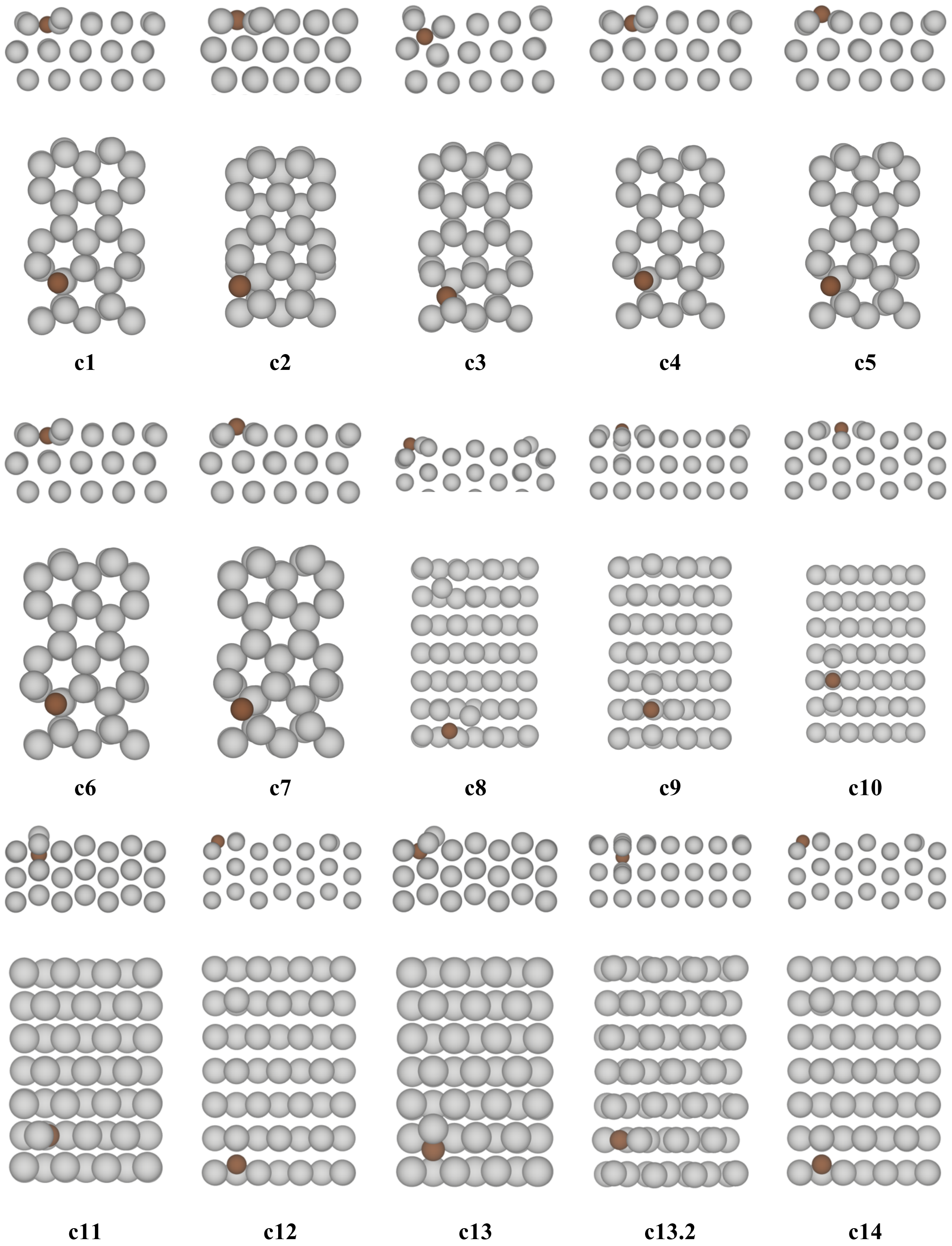

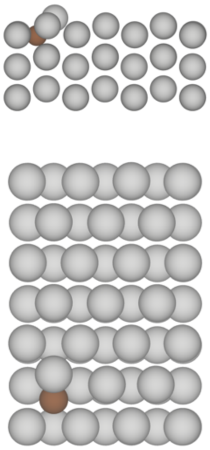

c15

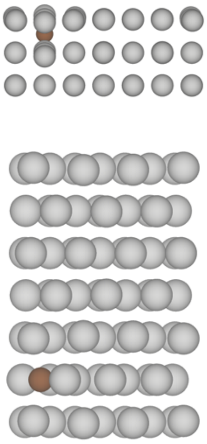

c15.2

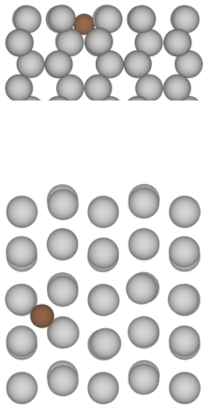

c16

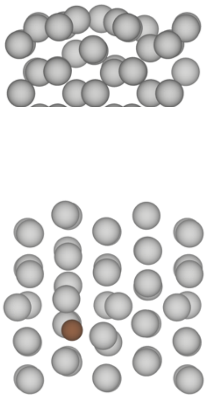

c17

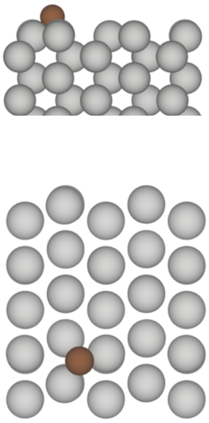

c17.2

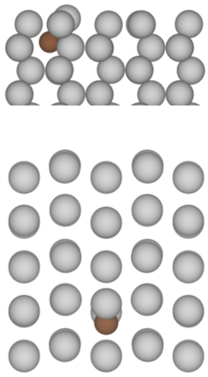

c18

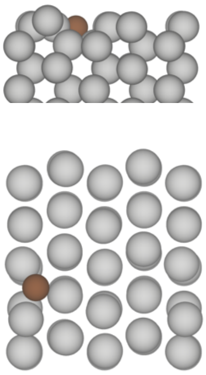

c19

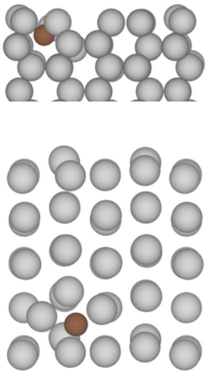

c20

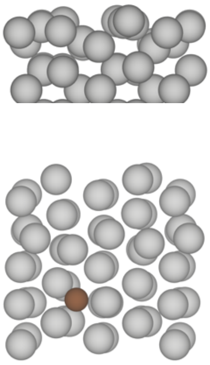

c21

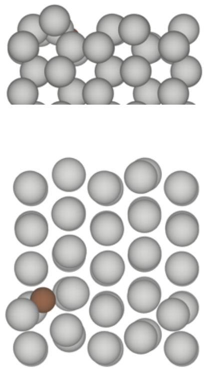

c21.2

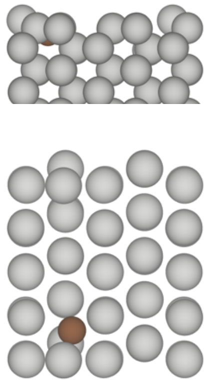

c21.3

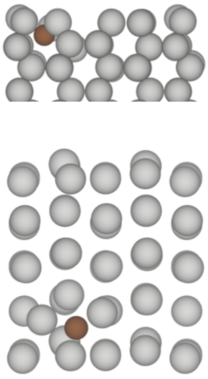

c22

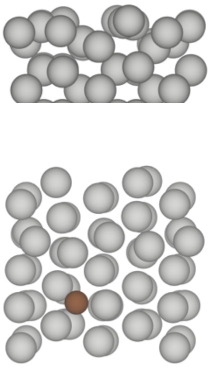

c23

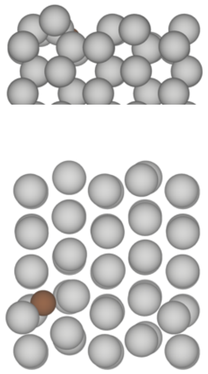

c23.2

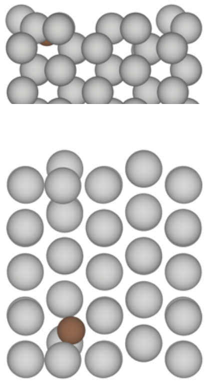

c23.3

Section S9: Diffusion Energy Barriers Isolated Descriptors

Figure S21. Linear correlation between diffusion energy barriers, E_b, and the TM surface *d*-band centre, ε_d. The regression coefficient, *R*, is displayed.

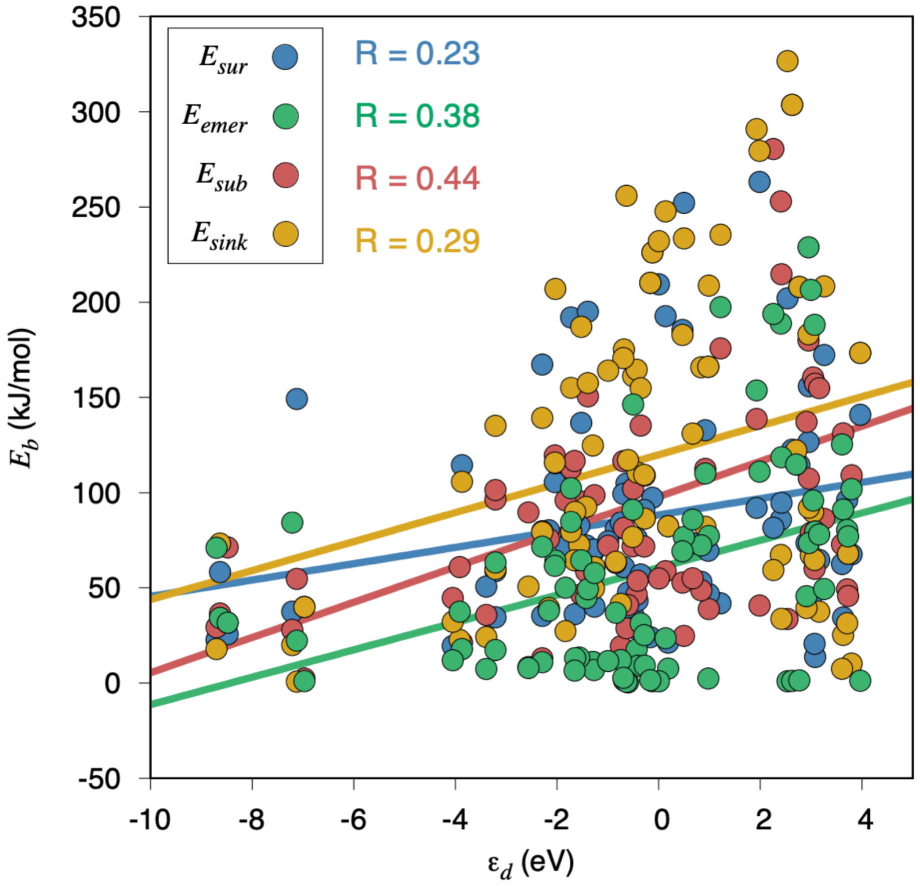

Figure S22. Linear correlation between diffusion energy barriers, E_b, and the TM surface width-corrected d-band centre, ε_d^W. The regression coefficient, R, is displayed.

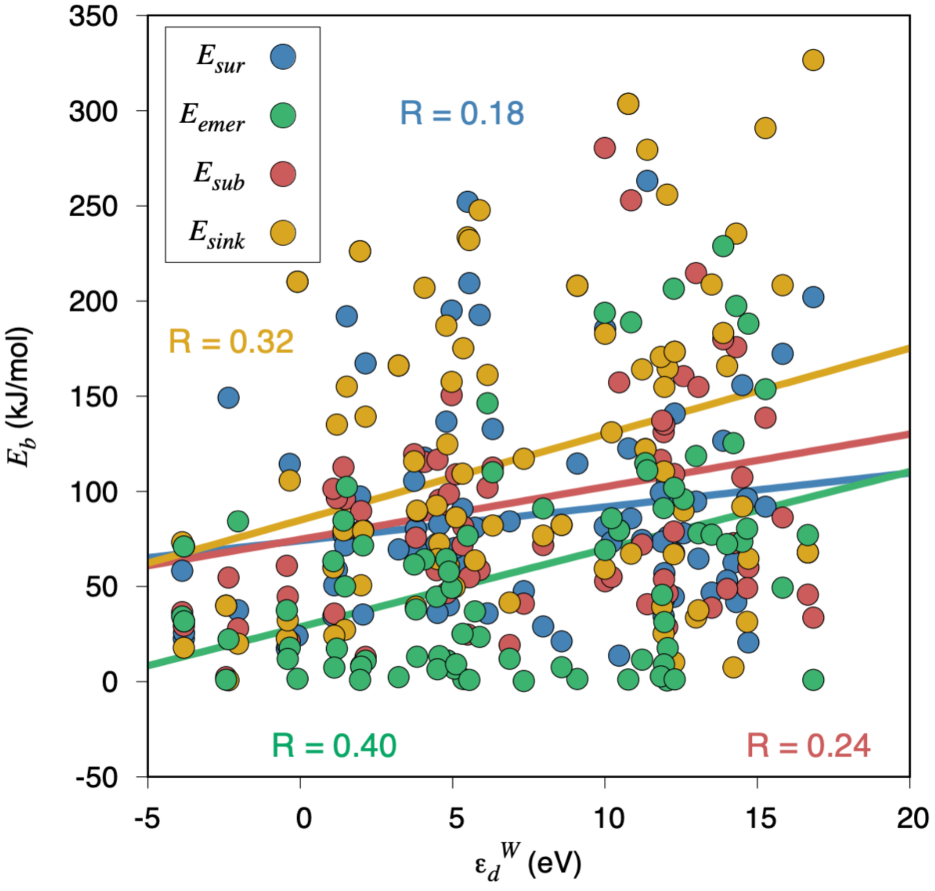

Figure S23. Linear correlation between diffusion energy barriers, E_b, and the TM surface maximum Hilbert peak, ε_u. The regression coefficient, *R*, is displayed.

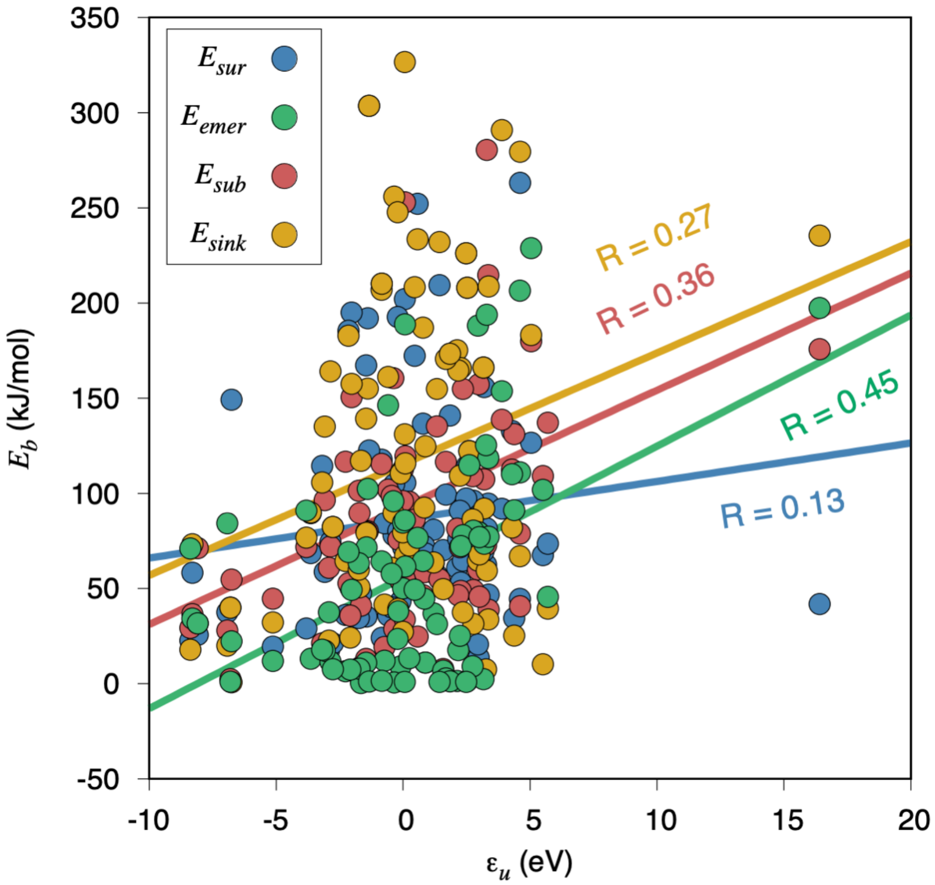

Figure S24. Linear correlation between diffusion energy barriers, E_b, and the TM surface energy, γ. The regression coefficient, *R*, is displayed.

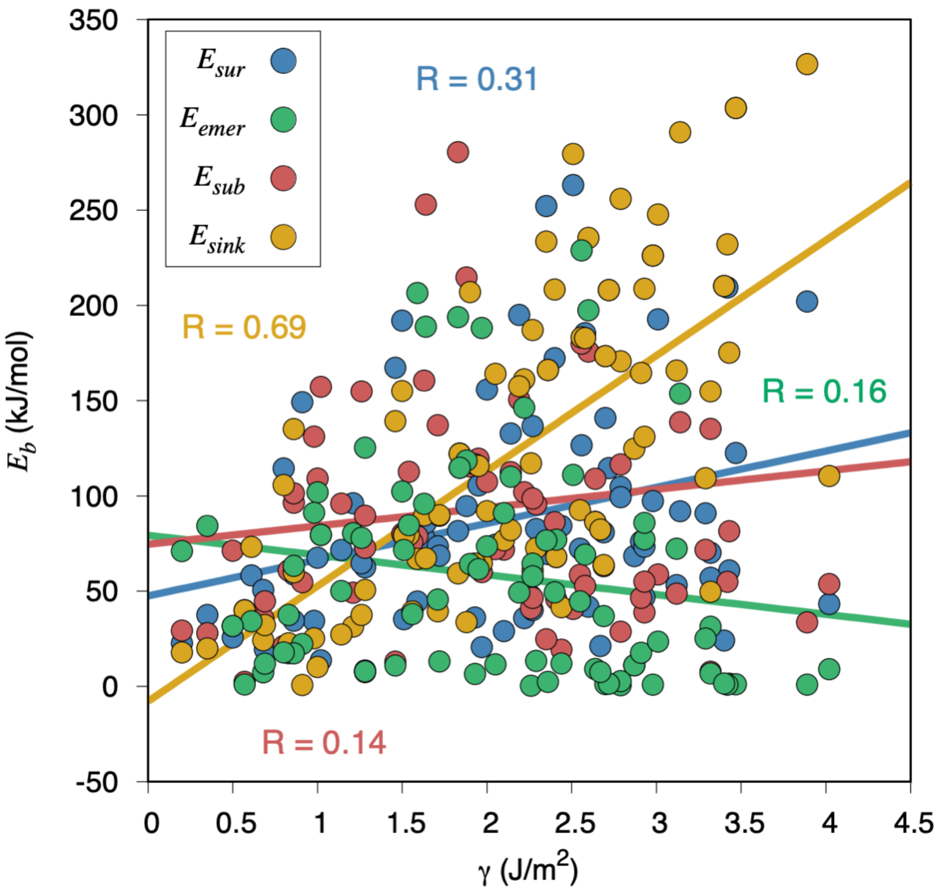

Figure S25. Linear correlation between diffusion energy barriers, E_b, and the surface work function, ϕ. The regression coefficient, *R*, is displayed.

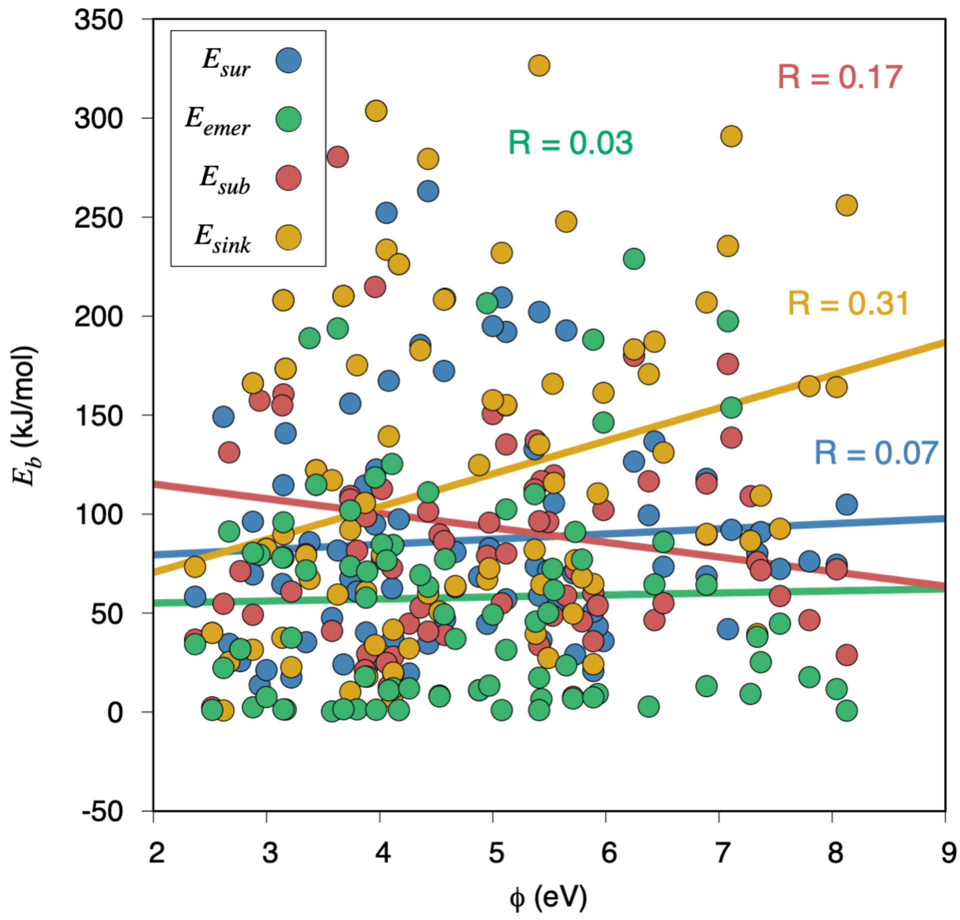

Figure S26. BEP linear evolutions of E_{sink} with respect to ΔE, and the corresponding linear correlations for each explored barrier type, as a function of the TM crystal structure. Dashed lines define latest TS limit, where $E_b = \Delta E$, or earliest TS limit, where $E_b = 0$ regardless of ΔE.

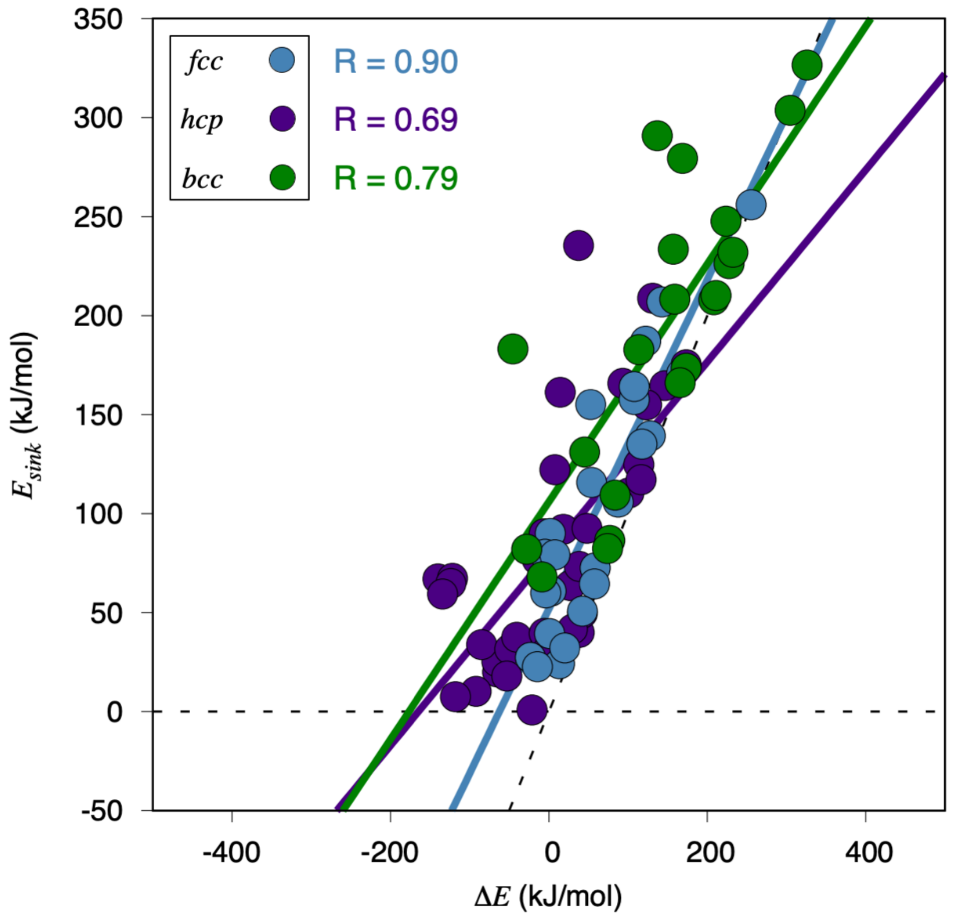

Figure S27. BEP linear evolutions of E_{emer} with respect to ΔE, and the corresponding linear correlations for each explored barrier type, as a function of the TM crystal structure. Dashed lines define latest TS limit, where $E_b = \Delta E$, or earliest TS limit, where $E_b = 0$ regardless of ΔE.

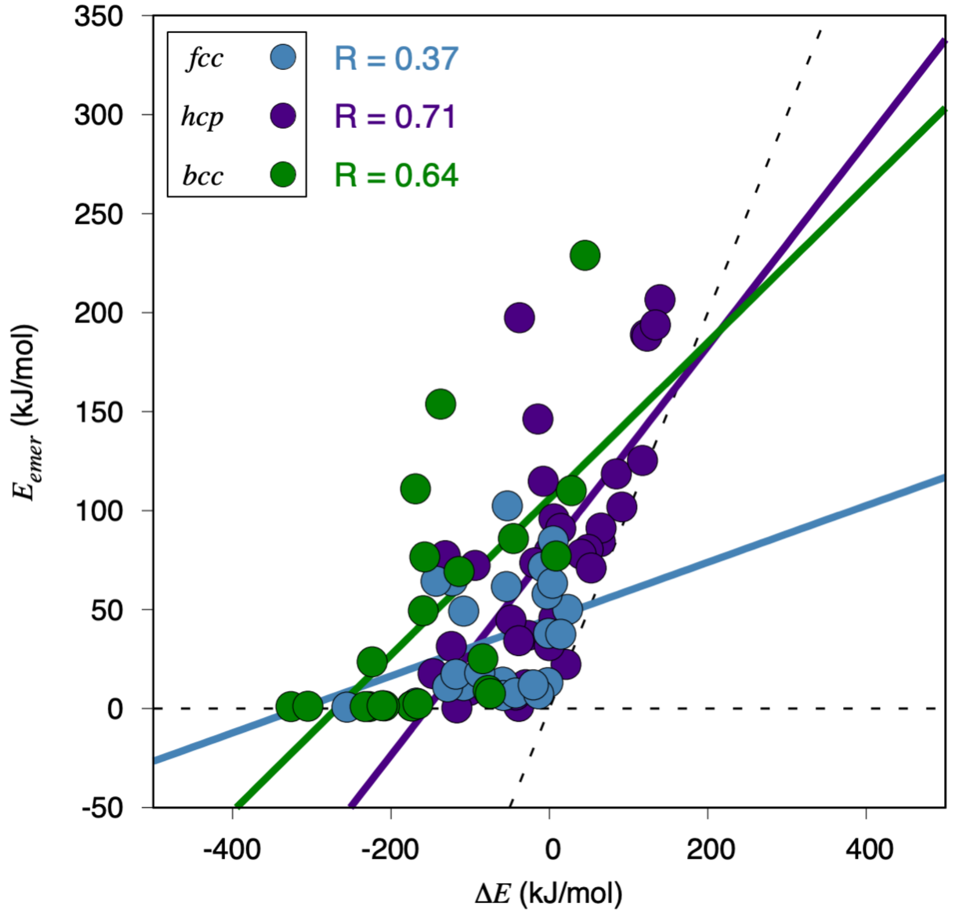

Section S10: Machine Learning Algorithms on Diffusion Energy Barriers

Figure S28. MAE evolution for training (blue) and the test (green) sets as a function of the number of samples contained in the training set for the prediction of E_{sur} using a RFR algorithm. Coloured areas around the lines account for the error dispersion resulting from the cross validation using 20 runs. The training and test sets MAEs yield values of 25.2 ± 6.6 and 10.4 ± 0.8 kJ·mol^{-1}, respectively.

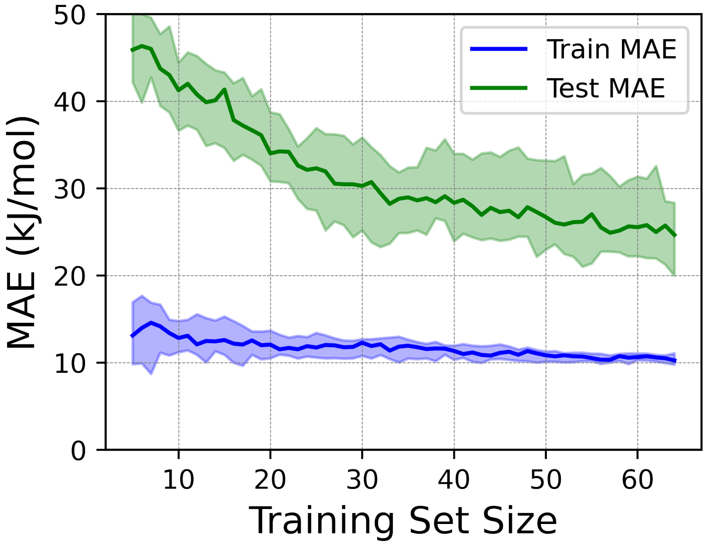

Figure S29. MAE evolution for training (blue) and the test (green) sets as a function of the number of samples contained in the training set for the prediction of E_{sub} using a RFR algorithm. Coloured areas around the lines account for the error dispersion resulting from the cross validation using 20 runs. The training and test sets MAEs yield values of 38.2 ± 7.1 and 15.7 ± 1.1 kJ·mol^{-1}, respectively.

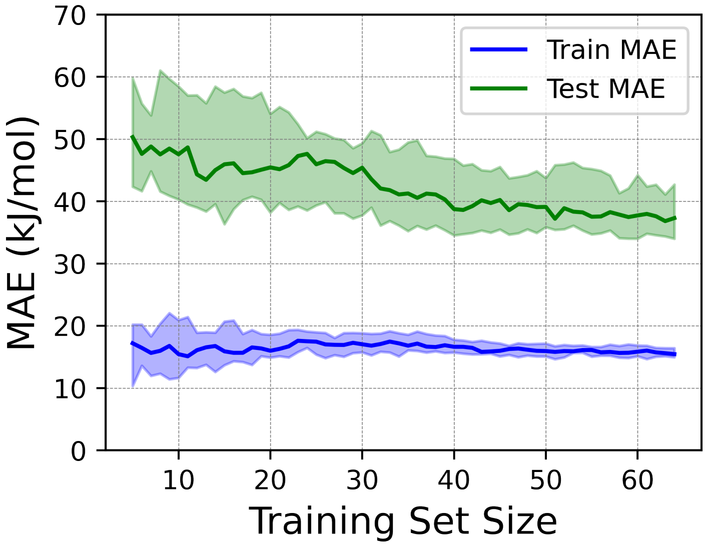

Figure S30. MAE evolution for training (blue) and the test (green) sets as a function of the number of samples contained in the training set for the prediction of E_{sink} using a RFR algorithm. Coloured areas around the lines account for the error dispersion resulting from the cross validation using 20 runs. The training and test sets MAEs yield values of 30.3 ± 5.6 and 11.1 ± 0.8 kJ·mol^{-1}, respectively.

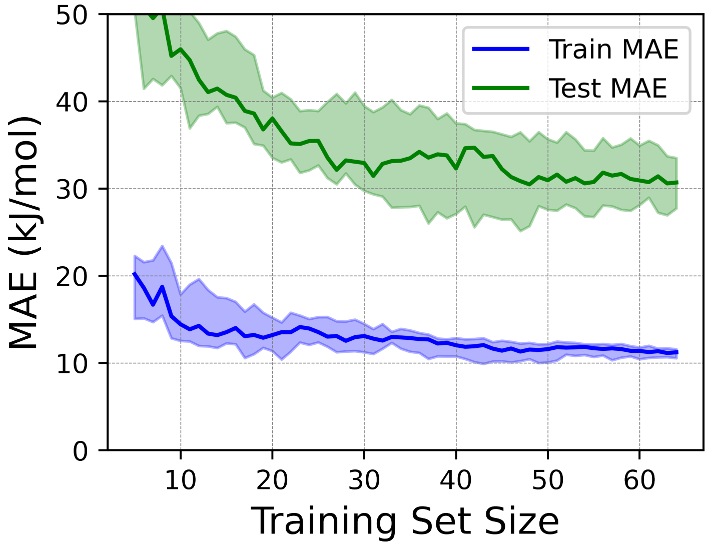

Figure S31. MAE evolution for training (blue) and the test (green) sets as a function of the number of samples contained in the training set for the prediction of E_{emer} using a RFR algorithm. Coloured areas around the lines account for the error dispersion resulting from the cross validation using 20 runs. The training and test sets MAEs yield values of 28.4 ± 6.0 and 10.6 ± 0.9 kJ·mol^{-1}, respectively.

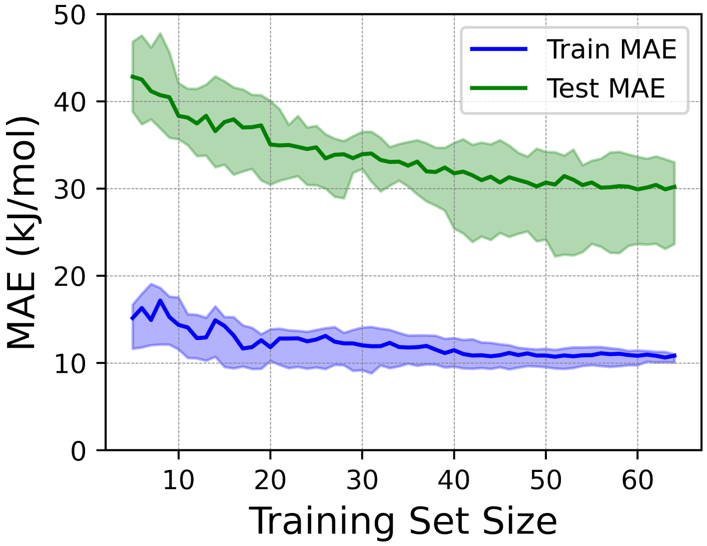

Table S11. Weight of the main features defining the Eb diffusion energy barriers, after applying the leave-one-out method on the RFR and MAE analysis shown in Figs. S27-S30.

	E_{sur}	E_{sub}	E_{sink}	E_{emer}
CN_{site}	0.174	0.138	—	—
CN_{FS}	0.316	0.021	—	—
ϕ	0.213	0.363	—	—
$E_{ads/abs}$	0.298	0.478	0.066	—
ΔE	—	—	0.603	0.628
δ	—	—	0.079	—
γ	—	—	0.251	—
ε_d	—	—	—	0.372

Section S11: Summary Table

Table S12. Summary table for all the surfaces studied in this work. It includes C preferred location (surface or subsurface), C preference for being isolated or grouped, C preferred diffusion (surface or subsurface), C preference for sinking or emerging, and to which of the thermodynamic and kinetic clusters each surface belongs.

TM	Surface	Preferred Location	C Cohesion or Isolation	Thermodynamic Cluster	Preferred Diffusion	Emergence or Sinking	Kinetic Cluster
Rh	**(001)**	Surface	Isolated	C3	Subsurface	Emerging	C3
	**(011)**	Surface	C Cohesion	C3	Surface	Emerging	C2
	**(111)**	Surface	C Cohesion	C3	Surface	Emerging	C1
Ir	**(001)**	Surface	Isolated	C3	Subsurface	Emerging	C3
	**(011)**	Surface	C Cohesion	C3	Surface	Emerging	C1
	**(111)**	Surface	C Cohesion	C3	Subsurface	Emerging	C1
Ni	**(001)**	Surface	Isolated	C3	Subsurface	Emerging	C2
	**(011)**	Surface	C Cohesion	C3	Surface	Emerging	C2
	**(111)**	Subsurface	C Cohesion	C2	Surface	Emerging	C1
Pd	**(001)**	Surface	Isolated	C3	Subsurface	Emerging	C2
	**(011)**	Surface	C Cohesion	C3	Surface	Sinking	C2
	**(111)**	Subsurface	C Cohesion	C2	Surface	Sinking	C2
Pt	**(001)**	Surface	C Cohesion	C3	Subsurface	Emerging	C2
	**(011)**	Surface	C Cohesion	C3	Surface	Emerging	C2
	**(111)**	Surface	C Cohesion	C3	Subsurface	Emerging	C2
Cu	**(001)**	Surface	C Cohesion	C3	Subsurface	Emerging	C3
	**(011)**	Surface	C Cohesion	C3	Surface	Emerging	C2
	**(111)**	Subsurface	C Cohesion	C1	Surface	Emerging	C1
Ag	**(001)**	Surface	C Cohesion	C1	Subsurface	Emerging	C3
	**(011)**	Subsurface	C Cohesion	C1	Surface	Sinking	C2
	**(111)**	Subsurface	C Cohesion	C1	Surface	Emerging	C2
Au	**(001)**	Surface	C Cohesion	C1	Surface	Emerging	C1

	**(011)**	Subsurface	C Cohesion	C1	Surface	Sinking	C2
	**(111)**	Surface	C Cohesion	C1	Subsurface	Emerging	C2
	**(001)**	Surface	Isolated	C3	Subsurface	Emerging	C3
**V**	**(011)**	Subsurface	Isolated	C2	Subsurface	Sinking	C2
	**(111)**	Surface	Isolated	C3	Surface	Emerging	C1
	**(001)**	Surface	Isolated	C3	Subsurface	Emerging	C3
**Nb**	**(011)**	Subsurface	Isolated	C2	Subsurface	Sinking	C2
	**(111)**	Surface	C Cohesion	C3	Surface	Emerging	C1
	**(001)**	Surface	Isolated	C3	Subsurface	Emerging	C3
**Ta**	**(011)**	Subsurface	Isolated	C2	Surface	Sinking	C2
	**(111)**	Surface	C Cohesion	C3	Surface	Emerging	C1
	**(001)**	Surface	Isolated	C3	Subsurface	Emerging	C3
**Cr**	**(011)**	Surface	C Cohesion	C3	Subsurface	Emerging	C2
	**(111)**	Surface	C Cohesion	C3	Surface	Emerging	C1
	**(001)**	Surface	Isolated	C3	Subsurface	Emerging	C3
**Mo**	**(011)**	Surface	C Cohesion	C3	Surface	Emerging	C1
	**(111)**	Surface	C Cohesion	C3	Surface	Emerging	C1
	**(001)**	Surface	Isolated	C3	Subsurface	Emerging	C3
**W**	**(011)**	Surface	Isolated	C3	Surface	Emerging	C2
	**(111)**	Surface	C Cohesion	C3	Surface	Emerging	C1
	**(001)**	Surface	Isolated	C3	Subsurface	Emerging	C3
**Fe**	**(011)**	Surface	Isolated	C3	Subsurface	Emerging	C2
	**(111)**	Surface	C Cohesion	C3	Surface	Emerging	C1
	**(0001)**	Subsurface	Isolated	C2	Surface	Sinking	C2
**Sc**	**$(10\bar{1}0)$**	Subsurface	Isolated	C2	Subsurface	Sinking	C2
	**$(11\bar{2}0)$**	Subsurface	Isolated	C2	Surface	Sinking	C2
	**(0001)**	Subsurface	Isolated	C2	Surface	Sinking	C2
**Y**	**$(10\bar{1}0)$**	Subsurface	Isolated	C2	Surface	Sinking	C2
	**$(11\bar{2}0)$**	Subsurface	Isolated	C2	Surface	Emerging	C2
**Ti**	**(0001)**	Subsurface	Isolated	C2	Surface	Sinking	C2

	**(10$\bar{1}$0)**	Surface	Isolated	C3	Subsurface	Emerging	C2
	**(11$\bar{2}$0)**	Subsurface	Isolated	C2	Surface	Sinking	C2
	**(0001)**	Subsurface	Isolated	C2	Surface	Sinking	C2
**Zr**	**(10$\bar{1}$0)**	Subsurface	Isolated	C2	Surface	Sinking	C2
	**(11$\bar{2}$0)**	Subsurface	Isolated	C2	Surface	Sinking	C2
	**(0001)**	Subsurface	Isolated	C2	Surface	Sinking	C2
**Hf**	**(10$\bar{1}$0)**	Surface	Isolated	C3	Subsurface	Emerging	C2
	**(11$\bar{2}$0)**	Subsurface	Isolated	C2	Surface	Sinking	C2
	**(0001)**	Surface	C Cohesion	C3	Surface	Emerging	C2
**Tc**	**(10$\bar{1}$0)**	Surface	Isolated	C3	Surface	Emerging	C1
	**(11$\bar{2}$0)**	Surface	C Cohesion	C3	Subsurface	Emerging	C2
	**(0001)**	Surface	C Cohesion	C3	Surface	Emerging	C2
**Re**	**(10$\bar{1}$0)**	Surface	C Cohesion	C3	Subsurface	Emerging	C2
	**(11$\bar{2}$0)**	Surface	C Cohesion	C3	Subsurface	Emerging	C2
	**(0001)**	Surface	C Cohesion	C3	Subsurface	Emerging	C2
**Ru**	**(10$\bar{1}$0)**	Surface	C Cohesion	C3	Subsurface	Emerging	C3
	**(11$\bar{2}$0)**	Surface	C Cohesion	C3	Subsurface	Emerging	C3
	**(0001)**	Surface	C Cohesion	C3	Subsurface	Emerging	C3
**Os**	**(10$\bar{1}$0)**	Surface	C Cohesion	C3	Surface	Emerging	C2
	**(11$\bar{2}$0)**	Surface	C Cohesion	C3	Surface	Emerging	C1
	**(0001)**	Surface	C Cohesion	C3	Surface	Sinking	C2
**Co**	**(10$\bar{1}$0)**	Surface	C Cohesion	C3	Subsurface	Emerging	C1
	**(11$\bar{2}$0)**	Surface	C Cohesion	C3	Subsurface	Emerging	C3
	**(0001)**	Subsurface	C Cohesion	C1	Subsurface	Sinking	C2
**Zn**	**(10$\bar{1}$0)**	Subsurface	C Cohesion	C1	Subsurface	Emerging	C3
	**(11$\bar{2}$0)**	Subsurface	C Cohesion	C1	Subsurface	Sinking	C2
	**(0001)**	Subsurface	C Cohesion	C1	Surface	Sinking	C2
**Cd**	**(10$\bar{1}$0)**	Subsurface	C Cohesion	C1	Subsurface	Emerging	C2
	**(11$\bar{2}$0)**	Subsurface	C Cohesion	C1	Surface	Emerging	C2

References

(1) Pedregosa, F.; Varoquaux, G.; Gramfort, A.; Michel, V.; Thirion, B.; Grisel, O.; Blondel, M.; Prettenhofer, P.; Weiss, R.; Dubourg, V.; Vanderplas, J.; Passos, A.; Cournapeau, D.; Brucher, M.; Perrot, M.; Duchesnay, E. Scikit-learn: Machine Learning in Python. *J. Mach. Learn Res.* **2011**, *12*, 2825-2830.

(2) Duivesteijn, W.; Feelders, A. J.; Knobbe, A. Interactive Knowledge Discovery from Hidden Data through Sampling of Frequent Patterns. *Data Min. Knowl. Discovery* **2016**, *30*, 47.

(3) Goldsmith, B. R.; Boley, M.; Vreeken, J.; Scheffler, M.; Ghiringhelli, L. M. Uncovering Structure-Property Relationships of Materials by Subgroup Discovery. *New. J. Phys.* **2017**, *19*, 013031.

(4) Chaker, Z.; Chervy, P.; Boulard, Y.; Bressanelli, S.; Retailleau, P.; Paternostre, M.; Charpentier, T. Systematic Method for the Exploration, Representation, and Classification of the Diphenylalanine Solvatomorphic Space. *J. Phys. Chem. B* 2021, **125**, 9454-9466.

(5) He, K. Filter Feature Selection for Unsupervised Clustering of Designer Drugs Using DFT Simulated IR Spectra Data. *ACS Omega* **2021**, *6*, 32151-32165.
